# Supplementary material for: Extracellular vesicles from differentiated stem cells contain novel proangiogenic miRNAs and induce angiogenic responses at low doses
Source: Mol Ther. 2023 Dec 13;32(1):185–203. doi: 10.1016/j.ymthe.2023.11.023 (PMC10787168; doi:10.1016/j.ymthe.2023.11.023)
Supplement: Document S1. Figures S1–S2 and Tables S2–S6 [file mmc1.pdf]

YMTHE, Volume *32*

Supplemental Information

Extracellular vesicles from differentiated stem cells contain novel proangiogenic miRNAs and induce angiogenic responses at low doses

Despoina Kesidou, Matthew Bennett, João P. Monteiro, Ian R. McCracken, Eftychia Klimi, Julie Rodor, Alison Condie, Scott Cowan, Andrea Caporali, Jan B.M. Wit, Joanne C. Mountford, Mairi Brittan, Abdelaziz Beqqali, and Andrew H. Baker

Table S1. Small RNA sequencing data. Supplemented as Excel file

Table S2. Antibodies used for staining human embryonic stem cell-derived endothelial cell products (hESC-ECPs) for flow cytometric characterisation. Cells stained for endothelial (CD31 and CD144) (right panels), pluripotent markers (TRA-181 and SSEA-4) (left panels), and their corresponding isotype controls.

	Antibody Name
Pluripotent	PE Mouse anti-human TRA-1-81 Antigen, BD Biosciences, 560161
	PE Mouse IgM, κ Isotype Control, BD Biosciences, 555584
	Alexa Fluor 647 Mouse anti-SSEA-4, BD Biosciences, 560796
	APC, anti-human, SSEA-4 Antibody, REAfinity, Miltenyi, 130-123-815
	Alexa Fluor 647 Mouse IgG3, κ Isotype Control, BD Biosciences, 560803
	PE, anti-human, TRA-1-60 Antibody, REAfinity, Miltenyi, 130-122-921
	PE, human IgG1, REA Control Antibody (S), REAfinity, Miltenyi, 130-113-438
Endothelial	PE Mouse anti-Human CD144, BD Biosciences, 580410
	PE Mouse IgG1, κ Isotype Control, BD Biosciences, 555749
	APC Anti-Human CD31, eBioscience, 17-0319-42
	APC Mouse IgG1, κ Isotype Control, eBioscience, 17-4714-42

Table S3. Specifications of samples analysed by small RNA sequencing.

Sample Name	RNA Concentration	RNA Sample Volume (ul)	Total RNA Quantity	Number of Flasks used for media collection	Total volume of media collected (ml)	Number of EVs isolated from the conditioned media	Number of Cells in the flasks used for media collection
hESC-ECP #1	278 ng/ul	30	8.3 µg	n/a	n/a	n/a	n/a
hESC-ECP #2	533 ng/ul	30	16 µg	n/a	n/a	n/a	n/a
hESC-ECP #3	154 ng/ul	30	4.6 µg	n/a	n/a	n/a	n/a
hESC-eEV #1	91 pg/ul	30	2.7 ng	3*T75 + 2*T25	80	4*10^10	8.8*10^6
hESC-eEV #2	35 pg/ul	30	1.1 ng	4*T25	36	1.8*10^10	3.2*10^6
hESC-eEV #3	116 pg/ul	30	3.9 ng	2*T75	40	4.6*10^10	4.8*10^6
hESC-mEV #1	234 pg/ul	30	7 ng	2*T25	16	1.2*10^11	n/a
hESC-mEV #2	743 pg/ul	30	22.3 ng	1*T75+ 1*T25	58	6.8*10^11	n/a
hESC-mEV #3	826 pg/ul	30	24.8 ng	1*T75+ 1*T25	58	9.8*10^11	n/a
HUVEC Hypoxic EV #1	39 pg/ul	30	1.2 ng	1*T150	15	1.2*10^10	6*10^6
HUVEC Hypoxic EV #2	103 pg/ul	30	3.1 ng	1*T150	15	1.9*10^10	6.3*10^6
HUVEC Hypoxic EV #3	75 pg/ul	30	2.3ng	4*T150	60	10^11	3.4*10^7
RC11-eEV #1	170 pg/ul	30	5.1ng	3*T75	66	1.5*10^11	n/a
RC11-eEV #2	222 pg/ul	30	6.7ng	3*T75	66	6.5*10^11	n/a
RC11-eEV #3	211.5 pg/ul	30	6.4ng	3*T75	66	1.8*10^11	n/a

Table S4. **List of primary and secondary antibodies used for Western Blot.**

Primary Antibodies	Product ID/ Company	Host	Reactivity	Dilution
Calnexin	ab22595, Abcam	Rabbit	Human, Mouse, Rat	1:1000
CD63	sc-5275, Santa-Cruz Biotechnology	Mouse	Human, Mouse, Rat	1:500
CD81	sc-7637, Santa-Cruz Biotechnology	Mouse	Human, Mouse, Rat	1:500
CD9	sc-59140 , Santa-Cruz Biotechnology	Mouse	Human, Mouse, Rat	1:500
CD31	Ab76533, Abcam	Rabbit	Human, Mouse, Rat	1:5000
**Secondary Antibodies**	**Product ID/ Company**	**Host**	**Reactivity**	**Dilution**
IRDye® 800CW Goat anti-Mouse IgG(H+L)	926-32210, LICOR	Goat	Mouse	1:15,000
IRDye® 680RD Goat anti-Rabbit IgG (H+L) secondary antibody	926-68072, LICOR	Goat	Rabbit	1:15,000

Table S5. List of miRNA mimics used for miRNA overexpression.

Name	Target	Assay ID
miRIDIAN microRNA Mimic Negative Control #1	Negative Control (miRC)	CN-001000-01, Dharmacon
miRIDIAN microRNA Human hsa-miR-4496 - Mimic	miR-4496	C-302100-00, Dharmacon
miRIDIAN microRNA hsa-miR-4691-5p- Mimic	miR-4691-5p	C-302248-00, Dharmacon
miRIDIAN microRNA hsa-miR-126-5p - Mimic	miR-126-5p	C-300625-05, Dharmacon

Table S6. List of miRNA TaqMan probes used for qRT-PCR analysis.

miRNA ID	Assay ID
RNU48	001006, ThermoFisher Scientific
miR-4496	465234_mat, ThermoFisher Scientific
miR-4691-5p	463008_mat, ThermoFisher Scientific
miR-126-5p	002228, ThermoFisher Scientific

Table S7. ***In silico* analysis identifying novel hESC-eEV-miRNAs with a potential role in angiogenesis**. Supplemented as Excel file

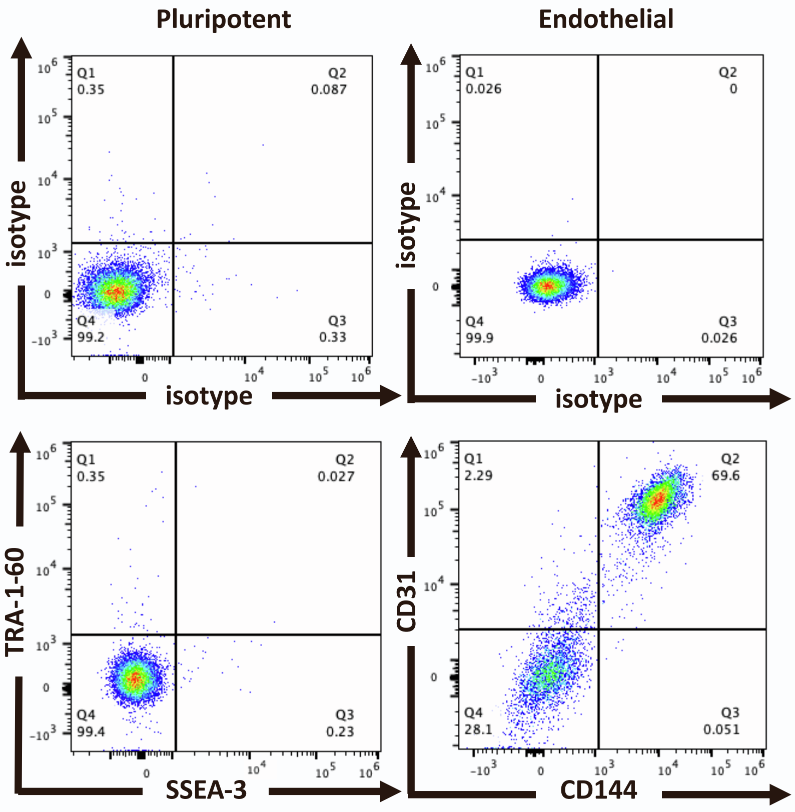

Figure S1. Representative flow cytometric analysis of human embryonic stem cell-derived endothelial cell products (hESC-ECPs). Cells were stained for endothelial (CD31 and CD144) (right panels), pluripotent markers (TRA-1-60 and SSEA-3) (left panels), and their corresponding isotype controls (top panels).

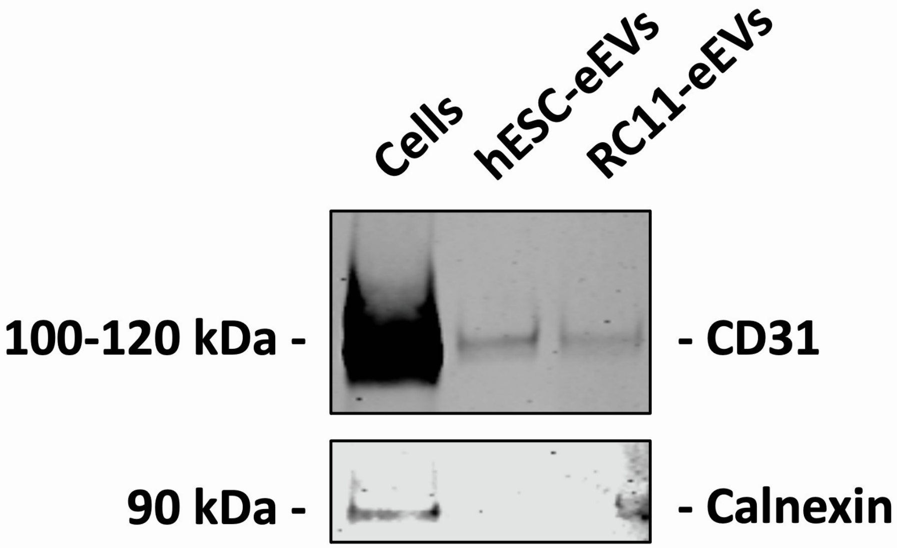

Figure S2. Western blot of Endothelial cell marker CD31 in hESC-eEVs from two independent cell lines H9 (hESC-eEVs) and RC11 (RC11-eEVs). Whole cell lysates of day 8 Endothelial cell product were used as a positive control. Calnexin was used as cellular protein control.

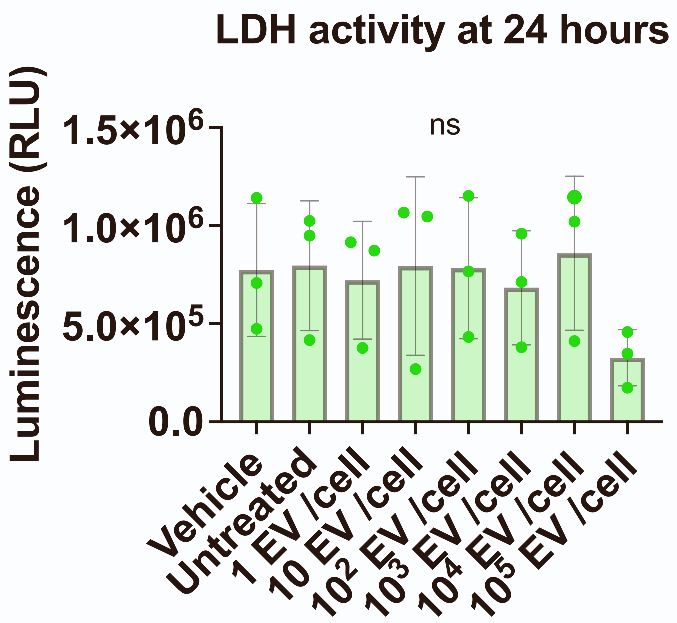

Figure S3. Lactate dehydrogenase activity (LDH) following treatment with extracellular vesicles from differentiated embryonic stem cells (hESC-eEVs). Plots represent LDH activity, quantified by luminescence in relative luminescence units (RLU) on the y-axis in response to treatment with increasing hESC-eEV doses (x-axis). LDH activity was measured at 24 hours (n=3). Statistical comparison was performed using one-way ANOVA with Dunnett's multiple comparisons test.

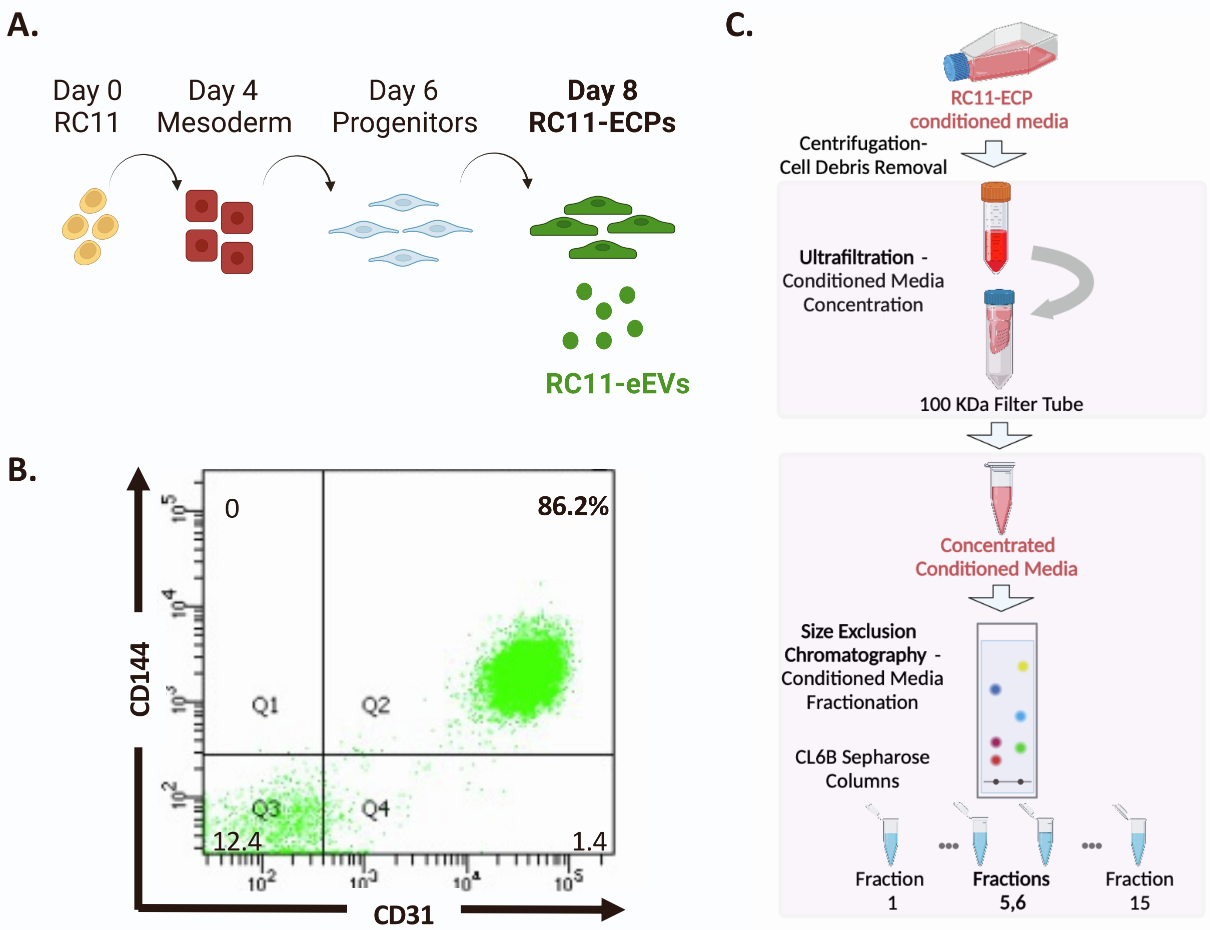

Figure S4. Isolation of extracellular vesicles (EVs) from RC11 human embryonic stem cell-derived endothelial cell product (RC11-ECP) conditioned media. A. Schematic representation of RC11-ECP differentiation and EV secretion from cells at the endothelial-enriched (day 8) stage. **B**. Representative flow cytometric analysis of RC11-ECP. Cells were stained for the endothelial markers CD31 and CD144. **C.** Workflow of EV isolation from RC11-ECP conditioned media by a combination of ultrafiltration with size exclusion chromatography (SEC). Created with BioRender.com

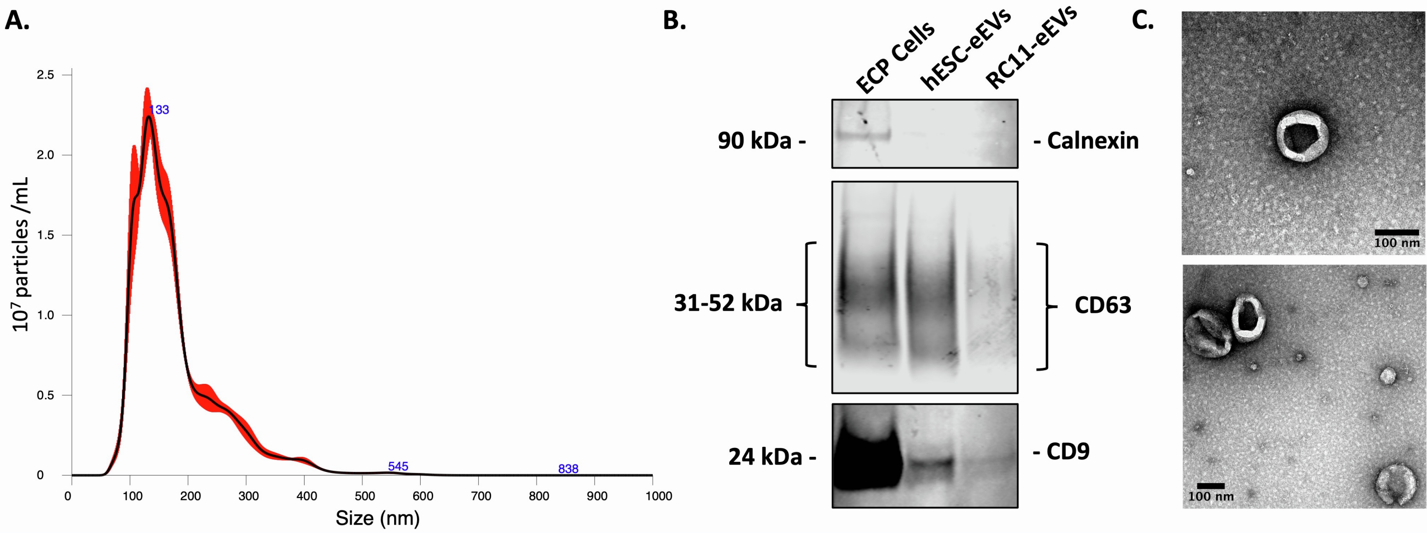

Figure S5. Characterisation of extracellular vesicles from RC11 human embryonic stem cell-derived endothelial cell products (RC11-eEVs).

A. Representative graph from nanoparticle tracking analysis (NTA) showing the size distribution and concentration of RC11-eEVs **B.** EV surface marker characterisation of RC11-eEVs by Western blot. Endothelial cell product lysates were used as a cellular protein control. **C.** Transmission electron microscopy (TEM) of extracellular vesicles from RC11 human embryonic stem cell-derived endothelial cell products (RC11-eEVs). Scale bar 100nm.

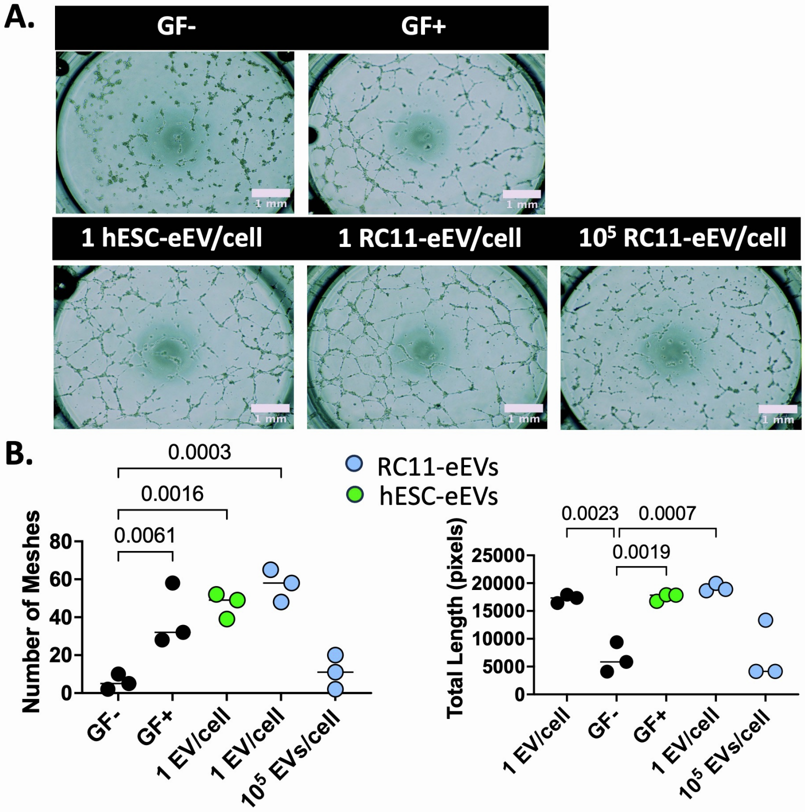

Figure S6. Extracellular vesicles from RC11 human embryonic stem cell-derived endothelial cell products (RC11-eEVs) induce endothelial cell (EC) tube formation at low concentrations. A. Tube Formation Assay on human cardiac microvascular endothelial cells (HCMECs) treated with two EV doses: 1 and 10^5 EVs/cell (n=3). Cells cultured in basal media and treated with sterile filtered PBS (vehicle control) served as negative control (GF-). Cells in fully supplemented media (GF+) or treated with extracellular vesicles from human embryonic stem cell-derived endothelial cell products (hESC-eEVs) served as positive controls. **B.** Quantification of the number of meshes, branches and the total length of the tubes formed at 4h. Images were analysed using the "Angiogenesis Analyzer" tool on ImageJ. The control samples (GF+ and GF-) are depicted as black circles, the hESC-eEV treated samples are depicted as green circles and the RC11-eEV-treated samples are depicted as blue circles. Statistical significance (indicated with p-values) was determined by one-way ANOVA with Dunnett's multiple comparisons test. Error bars represent the SD. Scale bar=1mm.

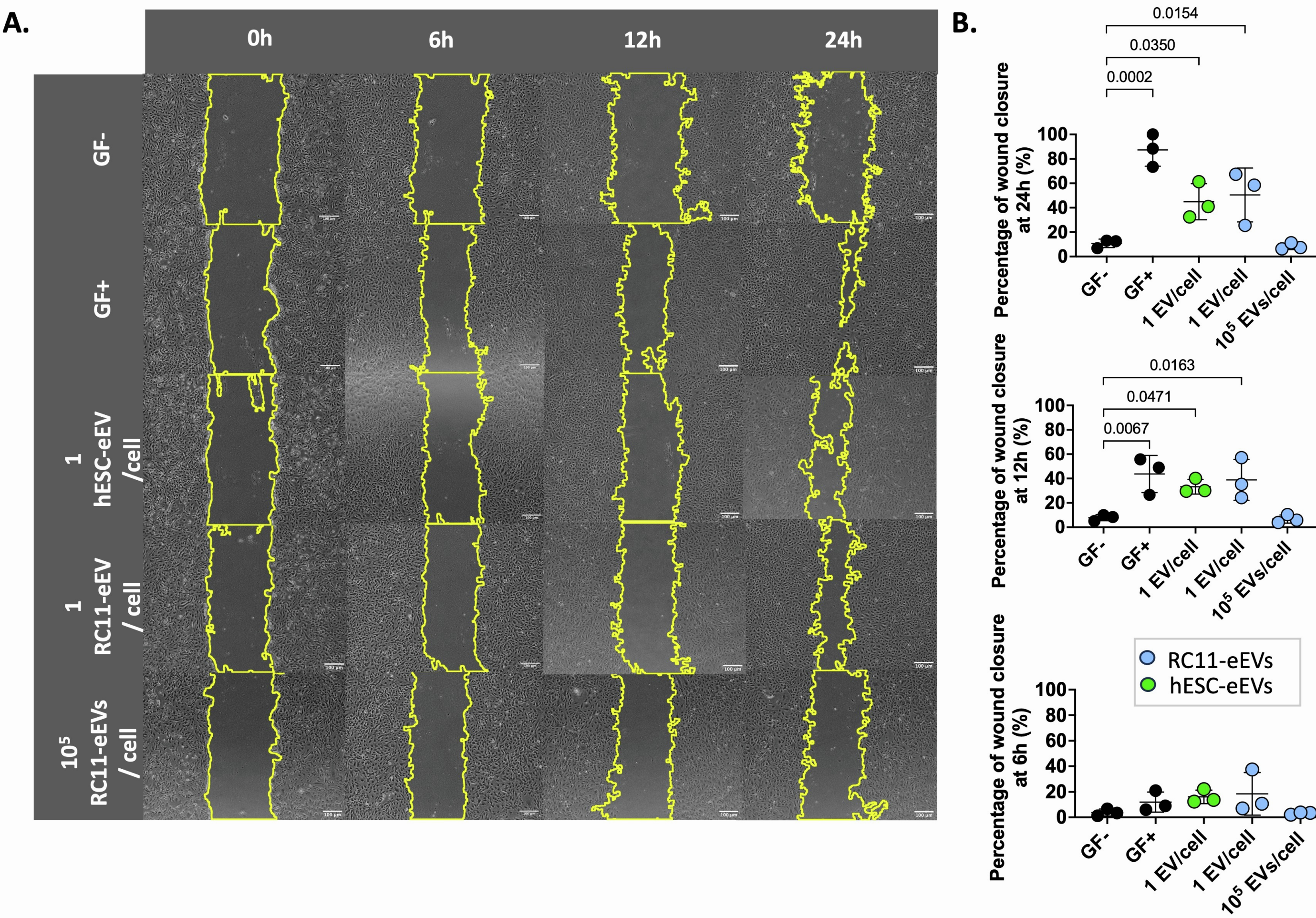

Figure S7. Extracellular vesicles from RC11 human embryonic stem cell-derived endothelial cell products (RC11-eEVs) promote endothelial cell (EC) wound healing at low concentrations. A) Wound Healing Assay on human cardiac microvascular endothelial cells (HCMECs) treated with EVs (n=3). Cells cultured in basal media and treated with PBS (vehicle control) served as negative control (GF-). Cells in fully supplemented media (GF+) or treated with extracellular vesicles from human embryonic stem cell-derived endothelial cell products (hESC-eEVs) served as positive controls. **B)** Quantification of the percentage of wound closure at 6h, 12h and 24h post-wound induction. The control samples (GF+ and GF-) are depicted as black circles, the hESC-eEV treated samples are depicted as green circles and the RC11-eEV-treated samples are depicted as blue circles. Statistical significance (indicated with p-values) was determined by one-way ANOVA with Dunnett's multiple comparisons test. Error bars represent the SD. Scale bar=100µm.

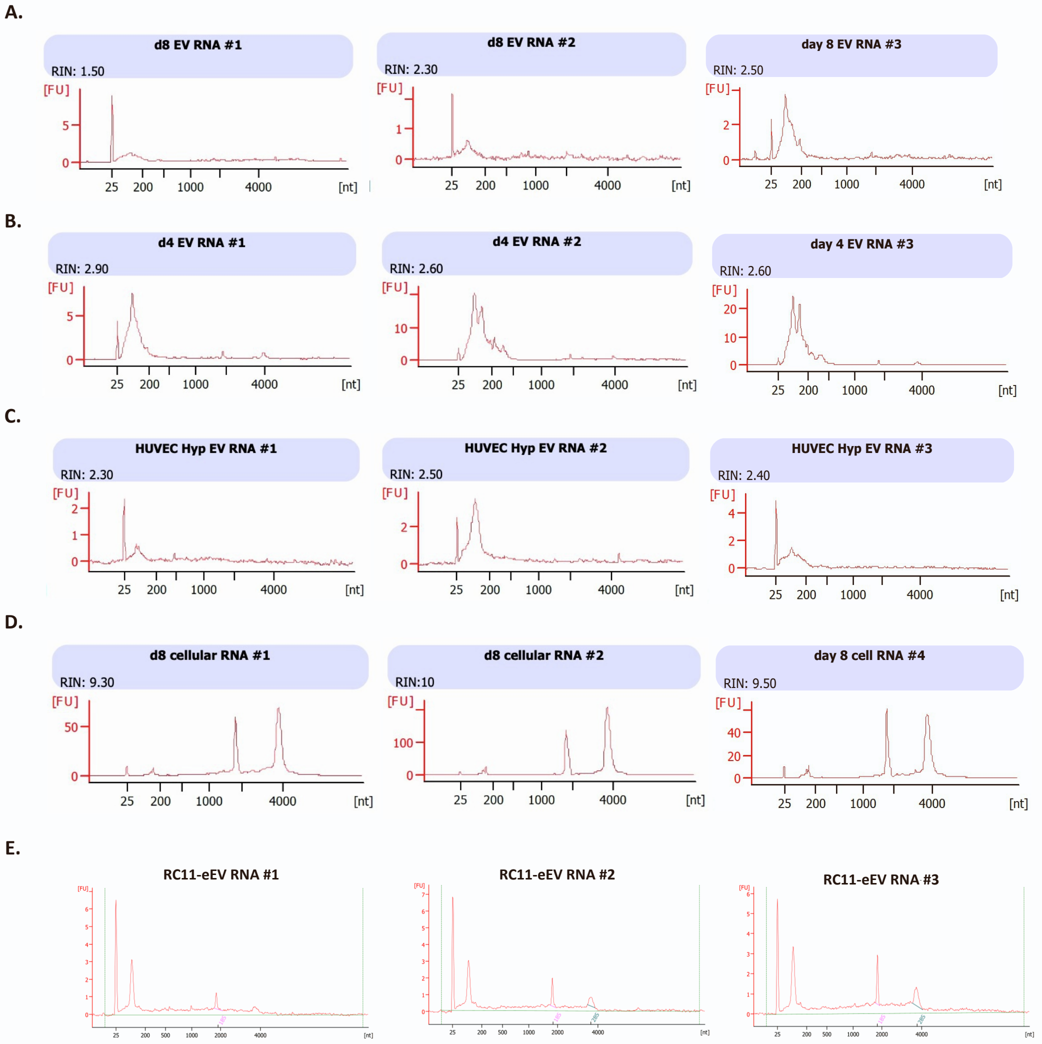

Figure S8. RNA profiling confirms the presence of small and the absence of ribosomal RNA peaks in the extracellular vesicle (EV) samples. A.RNA profiling of human embryonic stem cell-derived endothelial cell product EV (hESC-eEV) RNA samples (d8 EV RNA, n=3). **B.** RNA profiling of human embryonic stem cell-derived mesodermal cell product EV (hESC-mEV) RNA samples (d4 EV RNA, n=3). **C.** RNA profiling of human umbilical vein endothelial cell (HUVEC) hypoxic EV RNA samples (HUVEC Hyp EV RNA, n=3). **D.** RNA profiling of human embryonic stem cell-derived endothelial cell products (hESC-ECPs) RNA samples (d8 cellular RNA, n=3). **E.** RNA profiling of RC11 human embryonic stem cell-derived endothelial cell product EVs (RC11-eEVs , n=3)

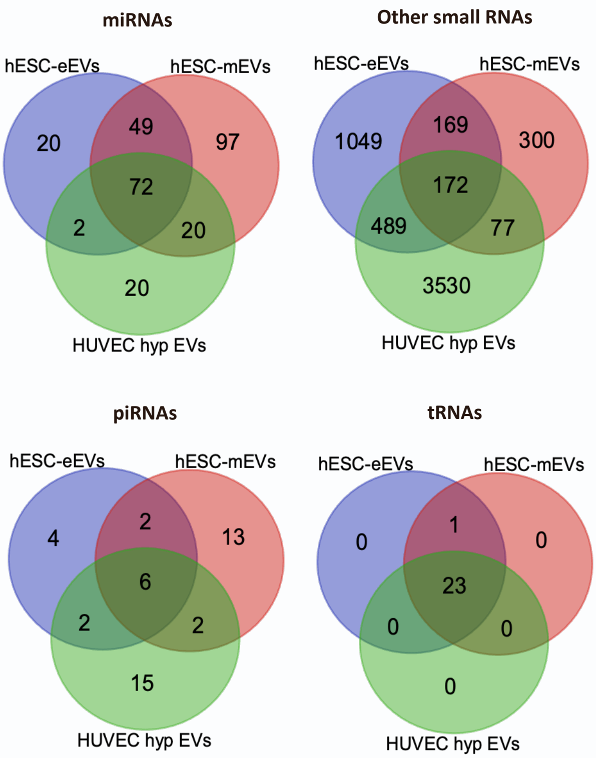

Figure S9. Small RNA sequencing showed a diverse composition of small RNA classes in the extracellular vesicle (EV) samples. Venn diagrams showing unique and common small RNA molecules present in the different EV samples. Molecules with average $RPMM_{total}$ <10 were filtered-out of the analysis.

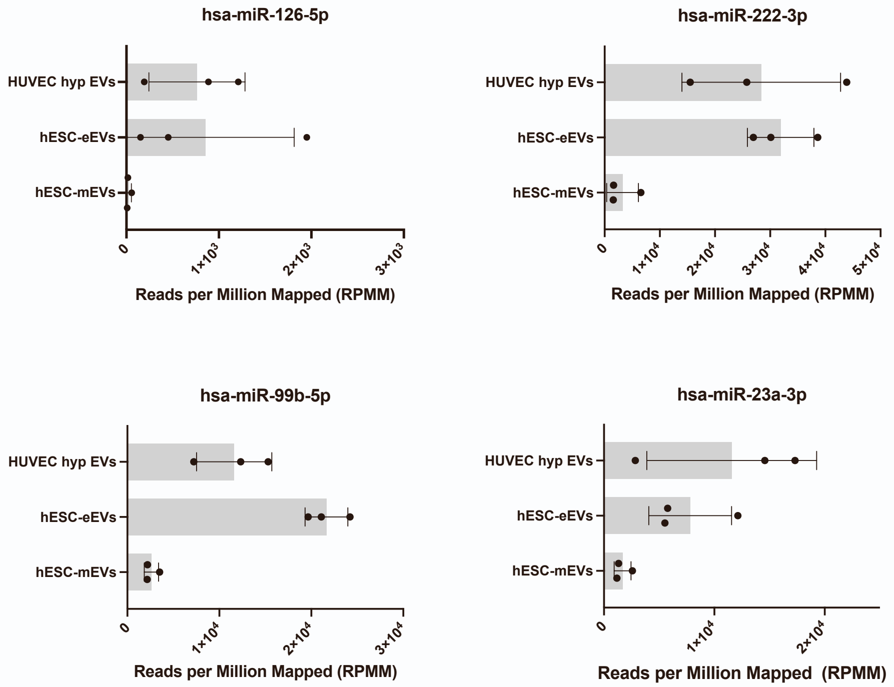

Figure S10. Extracellular vesicles from human embryonic stem cell-derived endothelial cell products (hESC-eEVs) and EVs from hypoxic human umbilical vein endothelial cells (HUVECs) express EC-enriched miRNAs. Plots representing RPMM of EC-enriched miRNAs (miR-126-5p, miR-222-3p, miR-99b-5p, miR-23a-3p) in the EV libraries.

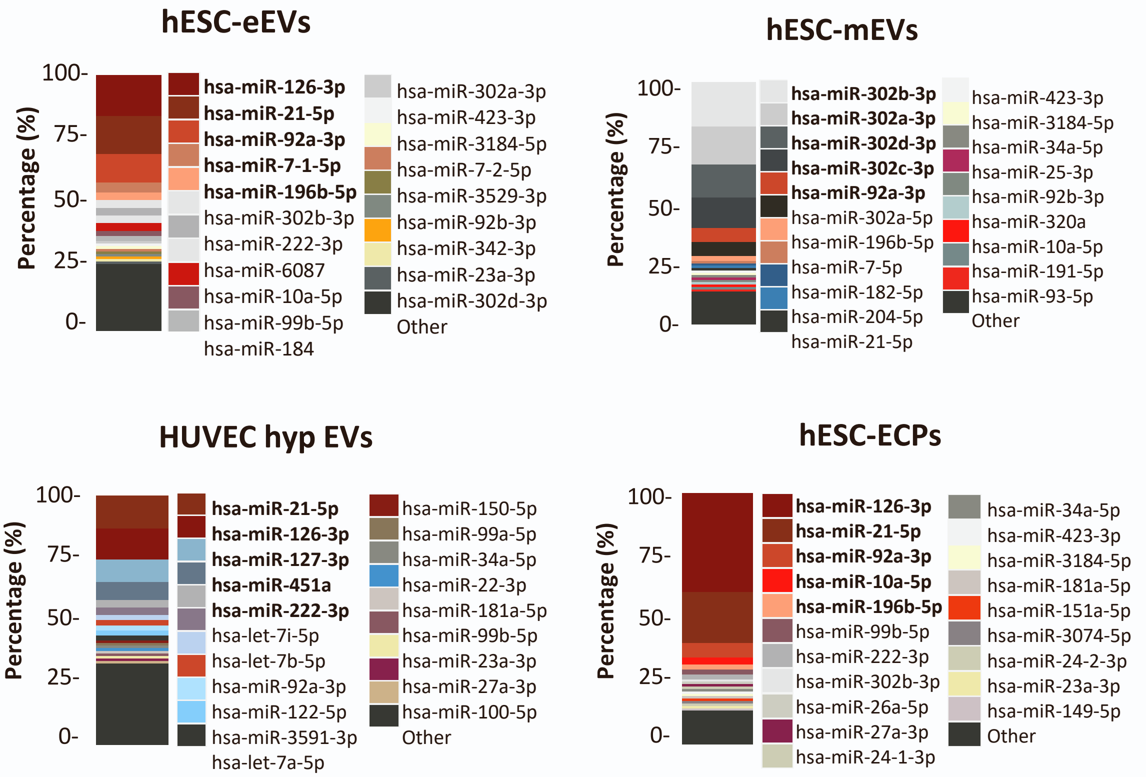

Figure S11. In each dataset the top 3-5 miRNAs occupy 50% of the total miRNA reads. Plots representing the percentage of the reads occupied by the top 20 miRNAs of each EV and cellular RNA sample. In all datasets the top 20 miRNAs correspond to 68-86% of the total miRNA reads. The top 5 miRNAs in each group are in bold. In the stacked bars each miRNA is represented by a different colour to understand the differences and similarities between the top 20 miRNA of each group.

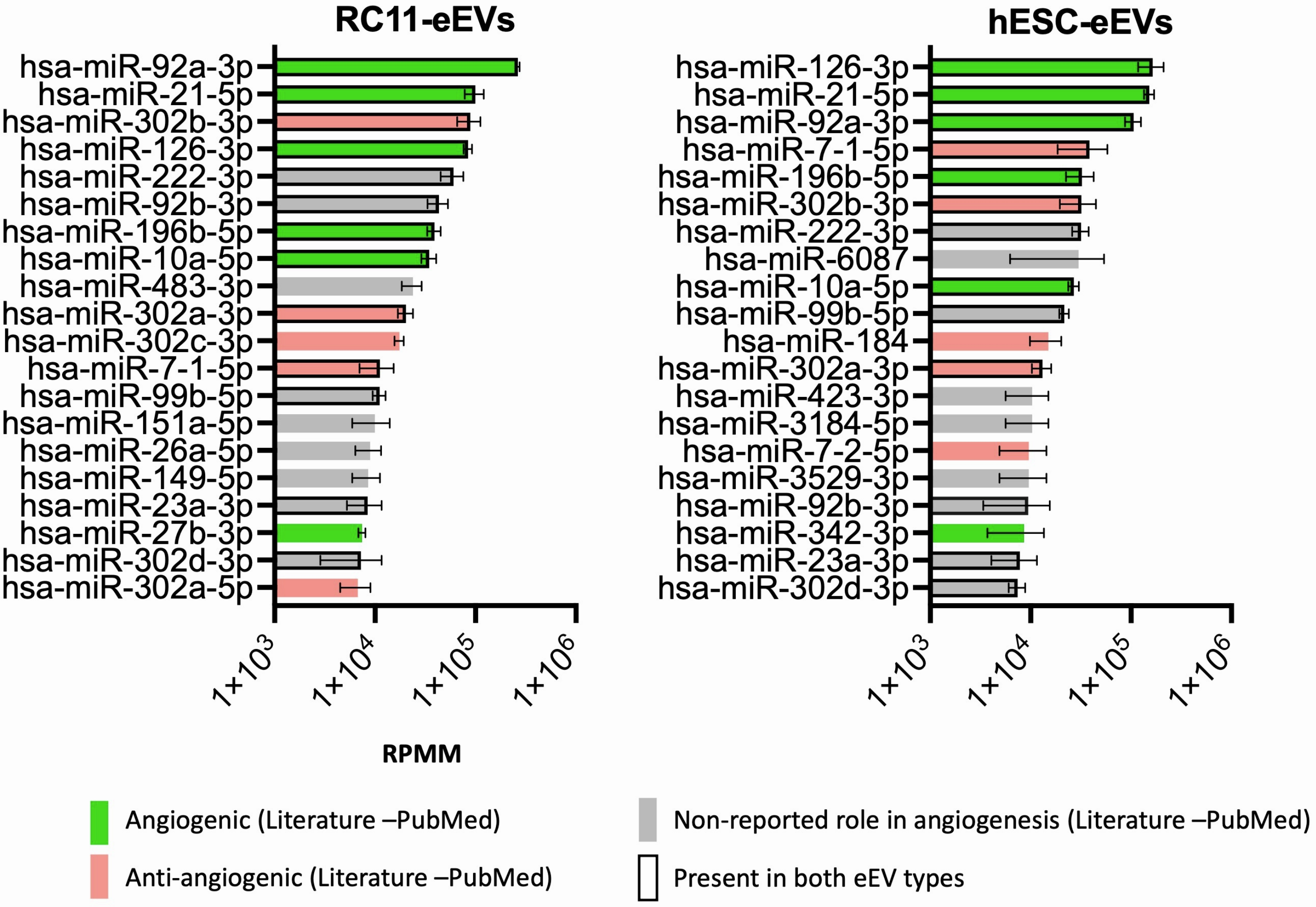

Figure S12. Top 20 microRNA cargo of RC11-eEVs and hESC-eEVs shows 65% overlap

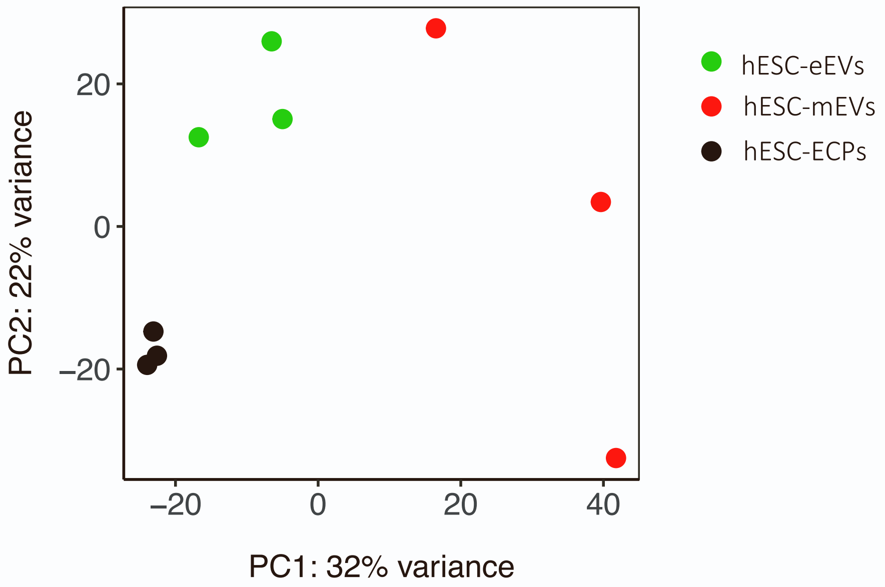

Figure S13. Principal Component Analysis (PCA) of the total reads of the Small RNA Sequencing datasets. Each dataset is depicted by a different colour. Extracellular vesicles from human embryonic stem cell-derived endothelial cell products (hESC-eEVs) are in green, extracellular vesicles from the mesodermal stage of the differentiation system (hESC-mEVs) are in red and human embryonic stem cell-derived endothelial cell products (hESC-EPCs) are in black.

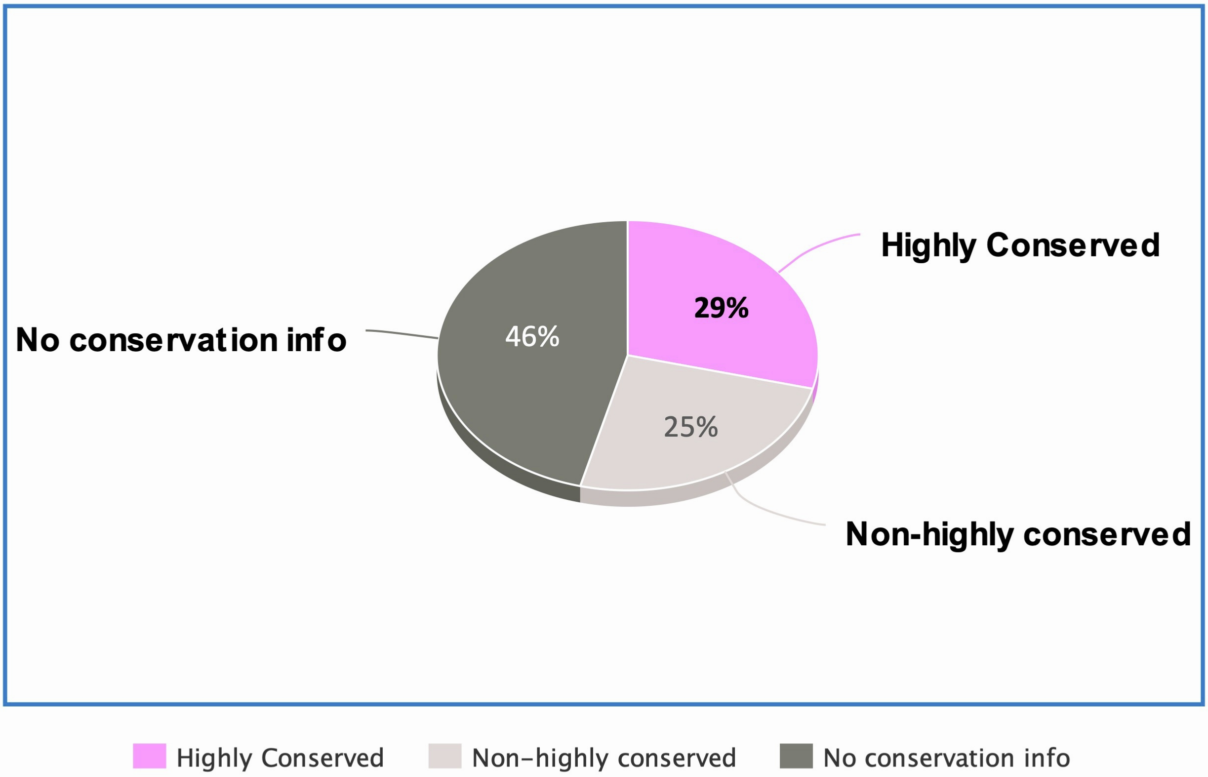

Figure S14. A small subset of human embryonic stem cell-derived extracellular vesicles miRNAs (hESC-eEV-miRNAs) is highly conserved between human and mouse/rat. To investigate the conservation of hESC-eEV-miRNAs between human and mouse/rat, we used the "microRNA viewer" tool. A cut-off of 95% was implemented to identify miRNAs that are highly conserved across the species examined. Dark grey: percentage of miRNAs with no conservation info with the informatic tools currently available. Pink: percentage of conserved miRNAs, Light grey: percentage of non-highly conserved miRNAs

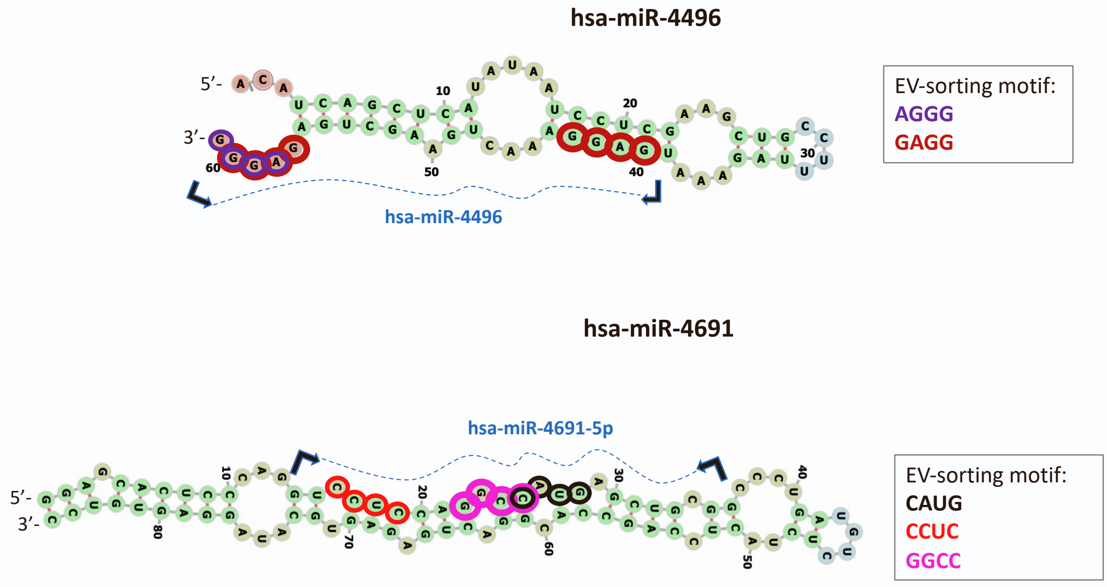

Figure S15. The human embryonic stem cell to endothelial cell extracellular vesicle (hESC-eEV)-enriched miRNAs miR-4496 and miR-4691-5p contain EV sorting motifs. Representation of the secondary structure of precursor miRNAs (pre-miRNAs) hsa-miR-4496 and hsa-miR-4691 generated using miRNAFold. Blue dotted lines represent the sequence of the mature miRNAs hsa-miR-4496 and miR-4691-5p. Nucleotides corresponding to different EV sorting motifs are in circles of different colours. Nucleotides corresponding to the AGGG motif are in dark purple circles. Nucleotides corresponding to the GAGG motif are in dark red circles. Nucleotides corresponding to the CAUG motif are in black. Nucleotides corresponding to the CCUC motif are in red. Nucleotides corresponding to the GGCC motif are in purple.

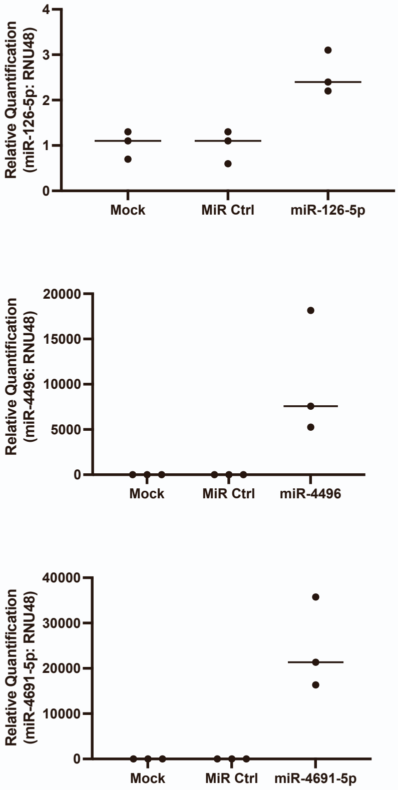

Figure S16. Endothelial cell transfection with miRNA mimics for miR-126-5p or miR-4496 or miR-4691-5p leads to miRNA overexpression. Results of qRT-PCR analysis showing significant upregulation of miR-126-5p or miR-4496 or miR-4691-5p expression in endothelial cells transfected with miRNA mimics compared to cells transfected with a miRNA control (miRC) or treated with lipofectamine RNAiMAX (mock) (n=3). RNU48 was used as a normalisation gene.

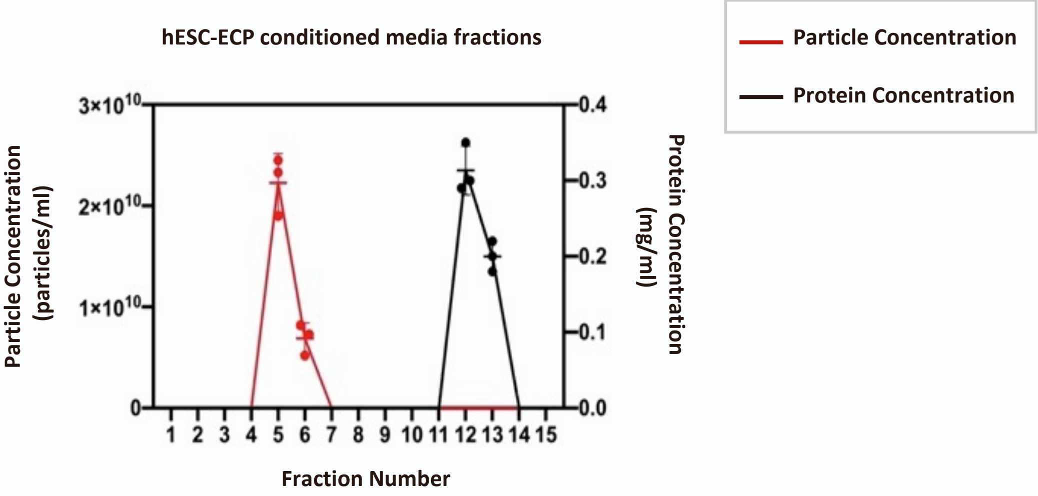

Figure S17. The combination of ultrafiltration with size exclusion chromatography (SEC) results in good separation of the particle and protein containing fractions. The graph depicts the particle and protein concentration of human embryonic stem cell-derived endothelial cell product (hESC-ECP) conditioned media fractions resulting after SEC, as determined by nanoparticle tracking analysis (NTA) and spectrophotometry respectively. Particles are eluted in fractions 5 and 6, while proteins are eluted in fractions 12 and 13.

A.

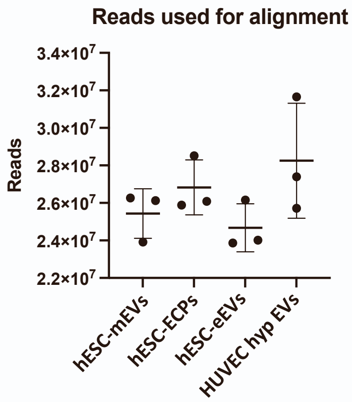

C.

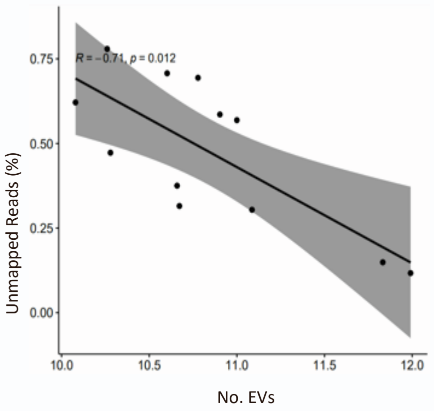

B.

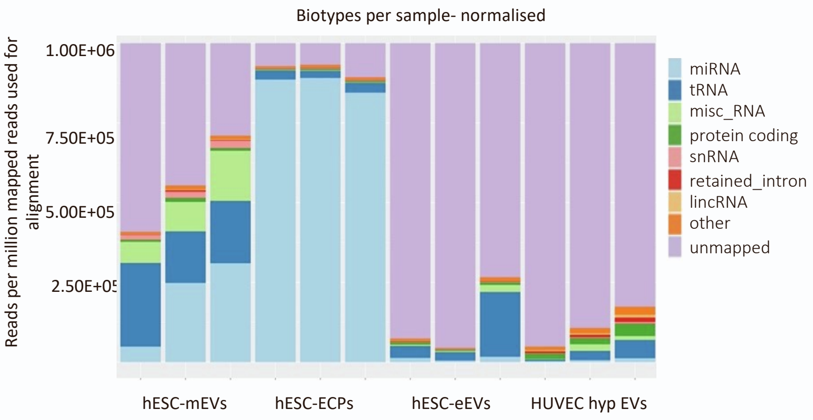

Figure S18. Small RNA sequencing read characteristics. A. RNA input reads used for alignment after filtering out UniVec/rRNA/low quality reads (n=3) **B.** Stacked bar plots representing read composition per sample. Reads are normalised to the total number of reads ($RPMM_{total}$) **C.** Correlation between the percentage of unmapped reads and the number of particles used in the small RNA sequencing of the extracellular vesicle (EV) samples.

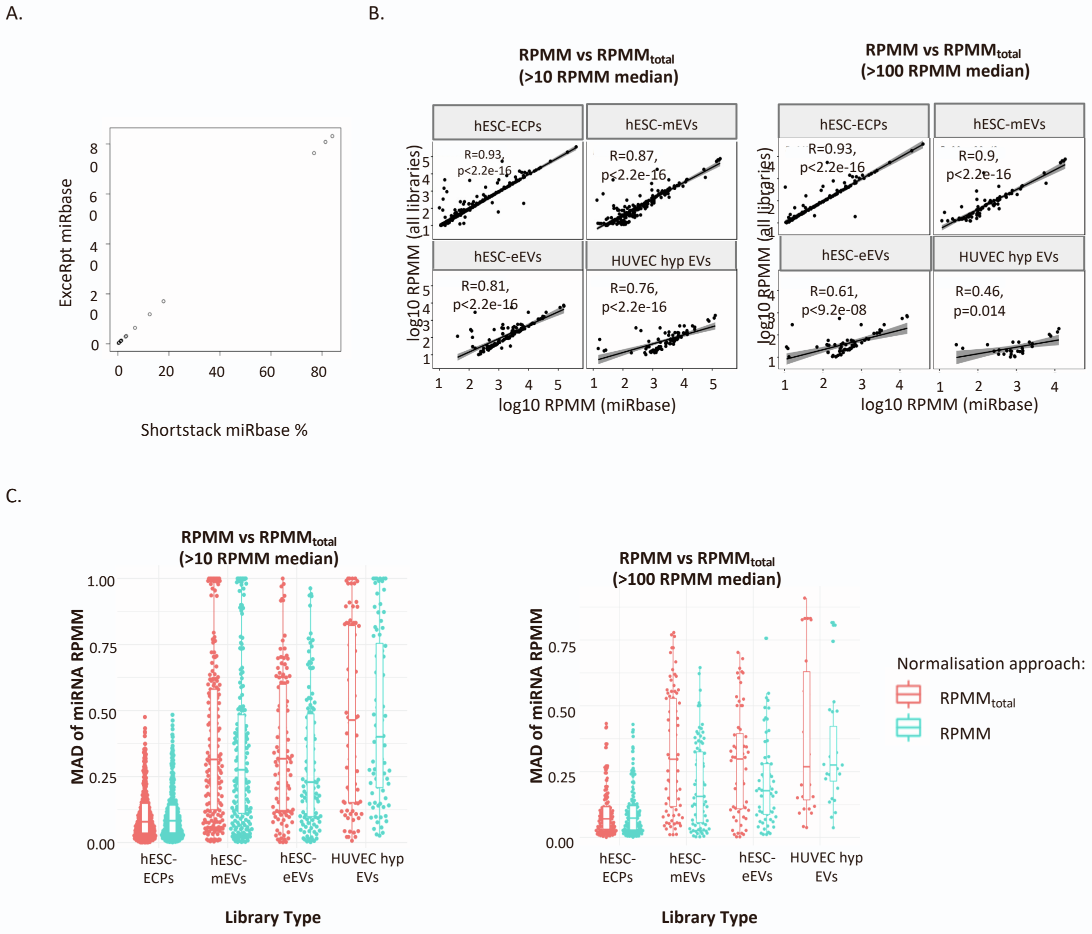

Figure S19. Normalisation to the total miRNA counts (RPMM method) results in lower variability to normalisation to normalisation to all small libraries (RPMM$_{total}$). A. Correlation between the results obtained by 2 normalisation approaches: 1) Normalisation of miRNA reads to the total small RNA reads (excerpt miRbase), 2) Normalisation of miRNA reads to the total RNA reads **B.** Graphs representing correlation between RPMM and RPMM$_{total}$ values in the different libraries **C.** Violin plots showing variability between replicates using RPMM and RPMM$_{total}$ normalisation approaches.

A.

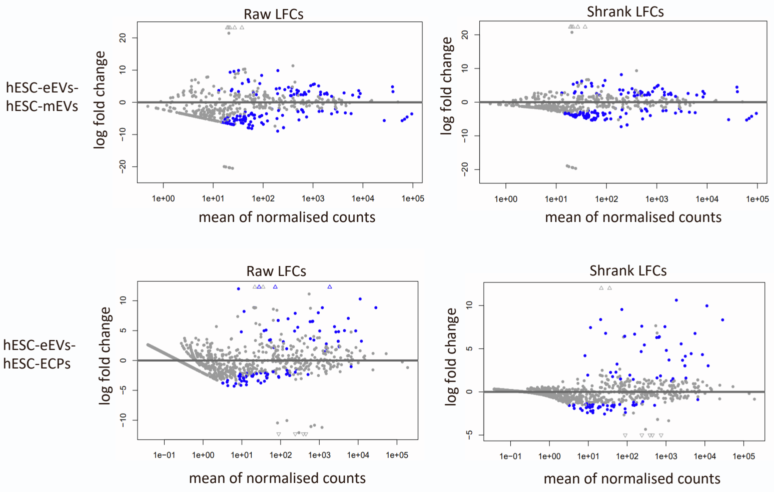

B.

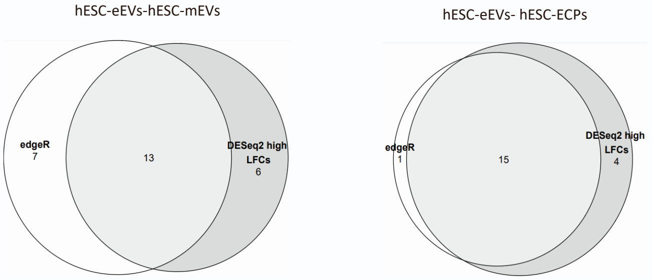

Figure S20. Differential expression analysis using the DESEq2 and edgeR tools yields similar results. A. Comparing log fold changes (LFCs) of miRNAs in the individual comparisons before (left panels) and after (right panels) applying fold change shrinkage via the ashr protocol in DESeq2. **B**. Comparison between DESeq2 and alternative normalisation and differential expression analysis via edgeR, high LFC refers to miRNAs with an LFC >5 or <-5 after shrinkage in DESeq2.

Movie S1. Visualisation of extracellular vesicles from human embryonic stem cell-derived endothelial cell products (hESC-eEVs) using Nanosight.
