# Supplementary material for: Extracellular vesicles from differentiated stem cells contain novel proangiogenic miRNAs and induce angiogenic responses at low doses
Source: Mol Ther. 2023 Dec 13;32(1):185–203. doi: 10.1016/j.ymthe.2023.11.023 (PMC10787168; doi:10.1016/j.ymthe.2023.11.023)
Supplement: Document S2. Article plus supplemental information [file mmc5.pdf]

Molecular Therapy
Original Article

Extracellular vesicles from differentiated stem cells contain novel proangiogenic miRNAs and induce angiogenic responses at low doses

Despoina Kesidou,[1] Matthew Bennett,[1] João P. Monteiro,[1,2] Ian R. McCracken,[1,3] Eftychia Klimi,[1] Julie Rodor,[1] Alison Condie,[4] Scott Cowan,[4] Andrea Caporali,[1] Jan B.M. Wit,[5] Joanne C. Mountford,[4] Mairi Brittan,[1] Abdelaziz Beqqali,[1,7] and Andrew H. Baker[1,6,7]

[1]Centre for Cardiovascular Science, University of Edinburgh, Edinburgh EH16 4TJ, UK; [2]Division of Cardiovascular Medicine, Stanford University School of Medicine, Stanford, CA 94305, USA; [3]Institute of Developmental and Regenerative Medicine, Department of Physiology, Anatomy, and Genetics, University of Oxford, Oxford OX3 7TY, UK; [4]Scottish National Blood Transfusion Service, Edinburgh EH14 4BE, UK; [5]Mirabilis Therapeutics BV, Maastricht, the Netherlands; [6]CARIM Institute, University of Maastricht, Maastricht 6229HX, the Netherlands

Extracellular vesicles (EVs) released from healthy endothelial cells (ECs) have shown potential for promoting angiogenesis, but their therapeutic efficacy remains poorly understood. We have previously shown that transplantation of a human embryonic stem cell–derived endothelial cell product (hESC-ECP), promotes new vessel formation in acute ischemic disease in mice, likely via paracrine mechanism(s). Here, we demonstrated that EVs from hESC-ECPs (hESC-eEVs) significantly increased EC tube formation and wound closure *in vitro* at ultralow doses, whereas higher doses were ineffective. More important, EVs isolated from the mesodermal stage of the differentiation (hESC-mEVs) had no effect. Small RNA sequencing revealed that hESC-eEVs have a unique transcriptomic profile and are enriched in known proangiogenic microRNAs (miRNAs, miRs). Moreover, an *in silico* analysis identified three novel hESC-eEV-miRNAs with potential proangiogenic function. Differential expression analysis suggested that two of those, miR-4496 and miR-4691-5p, are highly enriched in hESC-eEVs. Overexpression of miR-4496 or miR-4691-5p resulted in increased EC tube formation and wound closure *in vitro*, validating the novel proangiogenic function of these miRNAs. In summary, we demonstrated that hESC-eEVs are potent inducers of EC angiogenic response at ultralow doses and contain a unique EV-associated miRNA repertoire, including miR-4496 and miR-4691-5p, with novel proangiogenic function.

INTRODUCTION

Cardiovascular disease (CVD) remains the most common cause of mortality worldwide with the World Health Organization reporting that in 2017, 17.9 million people died of CVDs. Of those deaths, an estimated 7.4 million were attributed to coronary heart disease (CHD) alone.[1] CHD is characterized by the narrowing of the coronary arteries due to the gradual formation and subsequent rupture of plaque within the vessel walls. Blockage of these arteries results in oxygen and nutrient deprivation of the downstream tissue. Consequently, ischemic damage and cardiomyocyte death occur in the affected region of the heart, a phenomenon known as myocardial infarction (MI).[2] Therapeutic neovascularization has been proposed as a possible strategy to create new myocardial blood vessel networks and reduce the extent of cardiomyocyte damage. Paracrine cell communication plays a critical role in the control of this process.[3–5] Cell communication in a paracrine manner is regulated by several mechanisms, including extracellular vesicles (EVs). EVs carry and transfer various bioactive molecules, such as small noncoding RNAs, proteins, and lipids, that regulate signaling pathways in the recipient cells.[6]

Recently, EV microRNAs (EV-miRNAs) have gained immense interest due to their ability to control molecular processes in recipient cells, including angiogenesis.[7] miRNAs are small noncoding RNAs of approximately 22 nt and have been recognized as critical regulators of gene expression.[8] Nonetheless, there is great controversy regarding the relevance of miRNAs in EVs, including their endogenous function and possible therapeutic value. Several reports suggest that most extracellular miRNAs are protected from plasma ribonucleases by forming complexes with proteins such as AGO2 and that only a few copies are found in EVs.[9,10] Furthermore, numerous studies have demonstrated that, protected from

Received 16 May 2023; accepted 22 November 2023;
https://doi.org/10.1016/j.ymthe.2023.11.023.

[7]Senior author

Correspondence: Dr. Abdelaziz Beqqali, Centre for Cardiovascular Science, University of Edinburgh, Edinburgh EH16 4TJ, UK.
E-mail: a.beqqali@ed.ac.uk
Correspondence: Prof. Andrew H. Baker, Centre for Cardiovascular Science, University of Edinburgh, Edinburgh EH16 4TJ, UK.
E-mail: andy.baker@ed.ac.uk

This is an open access article under the CC BY license (http://creativecommons.org/licenses/by/4.0/).

plasma ribonucleases by their EV carriers, miRNAs can be delivered and internalized into recipient cells, acting as novel regulators of gene expression by inhibiting their targets.[11–14] Although nonselective secretion for some miRNAs has been proposed,[15] increasing evidence suggests that miRNAs are selectively sorted in EVs[16–20] due to the presence of specific motifs that facilitate their binding to RNA-binding proteins.[21–25] Despite this controversy, preclinical studies have demonstrated that EVs hold promise in the regulation of complex processes such as postischemic neovascularization.[11,26–33] However, the optimal source of such EVs, their respective cargo and function, as well as their translation to clinical opportunities remains in its relative infancy.

Endothelial cell (EC) injury and activation are important factors in the cellular release of EVs. In general, EC-EVs are present at lower concentrations under physiological conditions and, upon activation, are released at higher levels from ECs.[34–36] Circulating EVs released from ECs have been shown to play a role in activating vascular ECs.[37,38] Emerging evidence, however, suggests that EVs of endothelial origin may play a versatile role in neovascularization because their effect depends not only on the EV-donor cells but also on the dose or number of EVs to which the recipient cell is exposed.[39–44] Nonetheless, a potential role of the endothelial secretome in EC activation and neovascularization has been proposed by EC-transplantation studies that show improvements in capillary density and blood flow post-EC transplantation, despite limited retention to the ischemic tissues.[45,46] We recently reported a highly efficient human embryonic stem cell-to-EC differentiation protocol that consistently yields approximately 60%–70% $CD31^+$/$CD144^+$ ECs, with the remaining cells expressing pericyte and mesenchymal cell markers and exhibiting nonresidual pluripotency.[47,48] Transplantation of this ECP (hESC-ECP) following left femoral artery occlusion yielded a robust proangiogenic response across a range of mouse models.[47] Furthermore, we have shown improved cardiac function after hESC-ECP injection into the heart postmyocardial ischemia,[49] elucidating the broad relevance of these cells as a therapy. Despite their ability to increase capillary density, these cells showed poor retention,[47] suggesting that acute paracrine mechanisms of action may be involved. These findings were in line with previous cell transplantation studies, which have revealed that only a very few of the transplanted cells can engraft to the site of injury.[3–5,50–52]

Because the increased neovascularization rates post-hESC-ECP transplantation imply a paracrine mechanism of action, here, we sought to investigate the effect of EVs derived from hESC-ECP (hESC-eEVs) in angiogenesis. We demonstrated that hESC-eEVs at very low concentrations promote EC tube formation and wound closure *in vitro*. Transcriptomic profiling revealed that hESC-eEVs are enriched in known proangiogenic miRNAs, as well as miRNAs with a potentially novel angiogenic function (miR-4496, miR-4691-5p). The overexpression of miR-4496 or miR-4691-5p resulted in increased EC tube formation and wound closure *in vitro*. Overall, we demonstrated that hESC-eEVs induce angiogenic responses at low doses and identified the novel EV-associated miR-4496 and miR-4691-5p, which have novel proangiogenic function.

RESULTS

EVs isolated from hESC-ECP conditioned media express EV markers and show phenotypic characteristics of exosomes

To isolate hESC-eEVs, pluripotent hESCs underwent an established 8-day differentiation protocol via a mesodermal stage (Figure 1A), as previously described.[47–49] We confirmed that at day 8, approximately two-thirds (65.7%) of the cells expressed the EC surface markers CD31 and CD144, with fewer than 0.05% coexpressing the pluripotent markers SSEA-3 and TRA-1-60, as shown by flow cytometry (Figure S1). A combination of ultrafiltration and size exclusion chromatography (SEC) was used to isolate EVs from the hESC-ECP-conditioned media (Figure 1B). Enrichment of EVs was assessed by characterization of the EV size distribution, surface markers, and morphology. Nanoparticle tracking analysis (NTA) demonstrated that the size of the isolated particles ranged from 30 to 200 nm, with a mean value of 84 ± 7.3 nm (Figure 1C). Western blot analysis confirmed that the particles were positive for the EV markers CD63 and CD81 and negative for the endoplasmic reticulum marker calnexin (Figure 1D). Interestingly, the eEVs were also positive for the EC membrane protein CD31, suggesting that at least a subpopulation of the eEVs originate from the EC population at day 8 of differentiation (Figure S2). Using transmission electron microscopy (TEM), we identified structures of the characteristic size and shape of EVs. Quantification of the TEM images showed that the average EV size was 100.42 ± 38.36 nm and the roundness ratio was 0.96 ± 0.06 (Figure 1E). These high-purity EV preparations are henceforth referred to as hESC-eEVs.

hESC-eEVs promote tube formation and wound closure at low concentrations

To assess the angiogenic potency of hESC-eEVs we performed tube formation and wound closure assays using increasing concentrations of hESC-eEVs isolated from four independent differentiations (i.e., n = 4). Human cardiac microvascular ECs (HCMECs) cultured in media without growth factors and treated with equal volumes of sterile 0.1-μm filtered PBS (vehicle control) were used as negative controls, and HCMECs cultured in fully supplemented media were used as positive controls of angiogenic/migratory cells. Because hypoxia induces the release of proangiogenic EVs,[53] we also treated HCMECs with EVs obtained from hypoxic human umbilical vein ECs (HUVECs) as an EC-derived EV control. Performing dose-response experiments using HUVEC hypoxic EVs, we demonstrated that these EVs were ineffective at inducing EC tube formation at low doses (<100 EVs/cell) and showed the highest effect at high doses (10^5 EVs/cell) (Figure 2A). In contrast, treatment with approximately 1 hESC-eEV per cell resulted in significantly increased HCMEC tube formation compared to the negative control ($p < 0.0001$) (Figure 2B). We also showed that as opposed to HUVEC hypoxic EVs, treatment with a high hESC-eEV concentration (≥100 EVs/cell) failed to replicate this effect. Staining with calcein-acetoxymethylester (calcein-AM) confirmed cell viability in all of the conditions in the tube

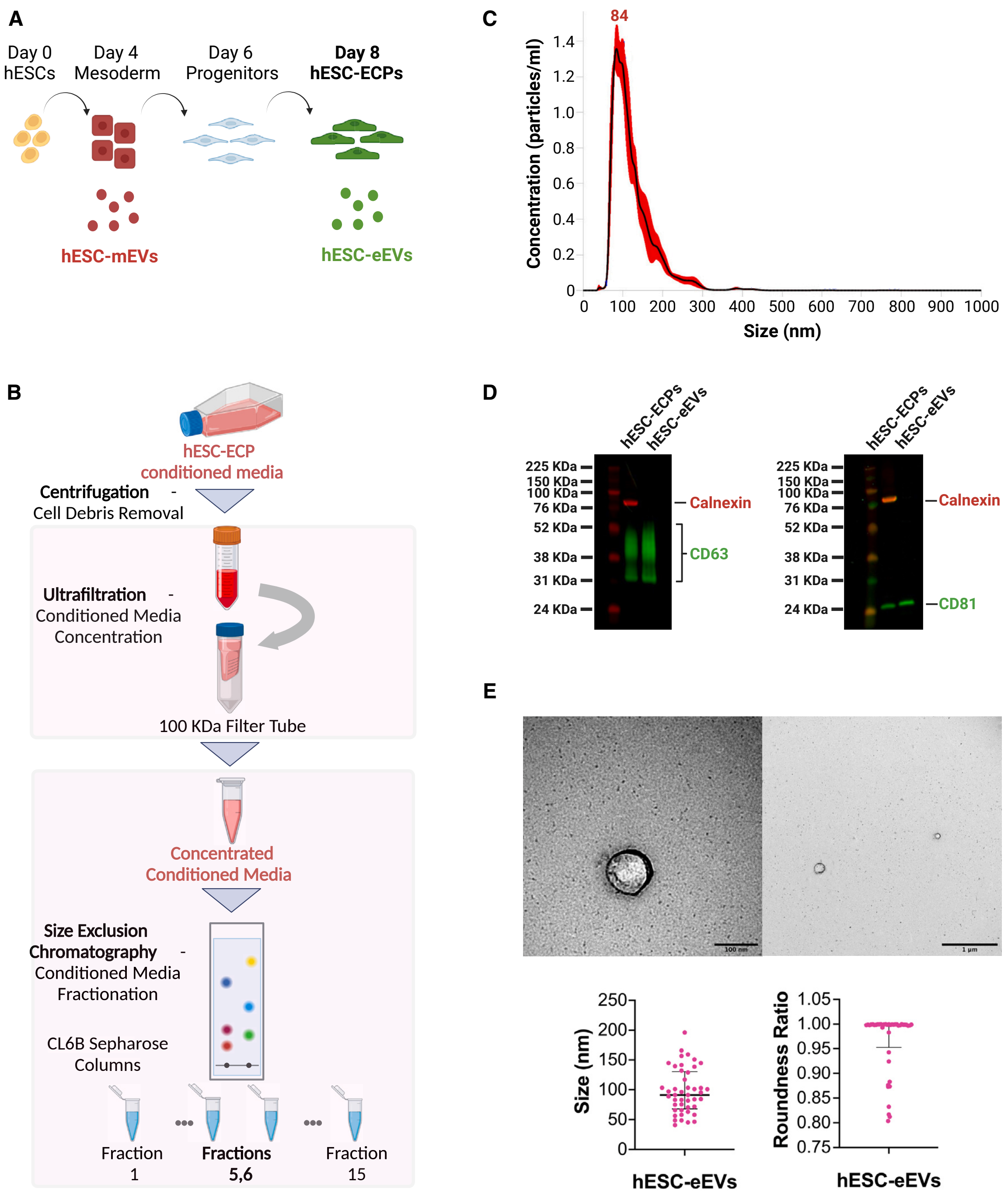

Figure 1. Isolation and characterisation of EVs from hESC-ECP-conditioned media

(A) Schematic representation of hESC-ECP differentiation and EV secretion from cells at the mesodermal (4) and the endothelial-enriched (d8) stage. (B) Workflow of particle isolation from hESC-ECP-conditioned media by a combination of ultrafiltration with SEC. Created with BioRender.com. (C) Representative graph from NTA showing the size distribution of EVs in the pooled fractions 5 and 6. (D) EV surface marker characterization of the pooled fractions 5 and 6 by western blot. Cell lysates were used as a positive control. Detection of calnexin emerges as a band at 90 kDa, detection of CD63 emerges at 30–65 kDa, and detection of CD81 emerges at 22–26 kDa. (E) TEM imaging of particles in the pooled fractions 5 and 6. The arrows indicate EVs. Scale bar, 100 nm (left) and scale bar, 1 μm (right). Scatterplots (below) represent the EV size distribution and roundness ratio (scale: 0–1) as determined by a total of 40 TEM images using the TEM Exosome Analyzer tool. Each point (in pink) corresponds to 1 EV. Error bars represent the median with interquartile range.

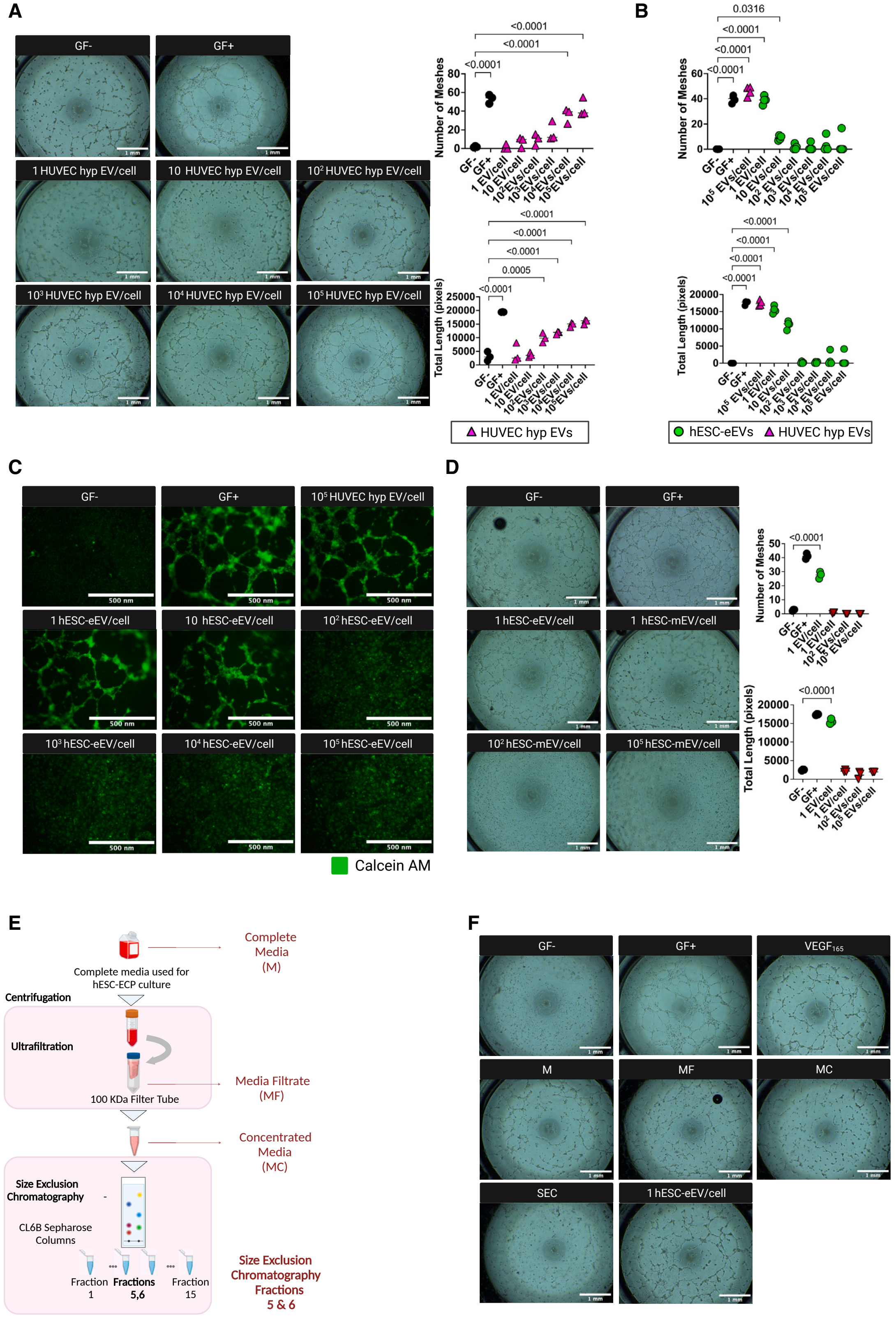

(legend on next page)

formation assay (Figure 2C). Furthermore, we have performed a lactate dehydrogenase assay to exclude that potential impurities or contaminants caused toxic effects at higher EV concentrations. At 24 h post-EV treatment we confirmed there is no evidence for cell toxicity (Figure S3).

To understand whether the effect on angiogenesis is specific for hESC-eEVs, we tested the angiogenic potential of EVs derived from the mesodermal stage of the differentiation protocol (hESC-mEVs). Our results showed that in contrast to treatment with one hESC-eEV/cell, hESC-mEVs did not induce tube formation in any dose tested (Figure 2D). To confirm that the proangiogenic effect observed post-hESC-eEV treatment is specific for the EVs and not for other soluble, cell culture media–associated factors, we subjected complete medium (unconditioned/not exposed to cells) to the EV purification process and collected samples at different stages of the purification (Figure 2E). These samples were tested for their ability to induce HCMEC tube formation (Figure 2F). Our results showed that treatment with complete medium subjected to the EV purification process does not improve HCMEC tube formation ability, whereas treatment with EVs isolated from conditioned media (1 hESC-eEV/cell) promoted HCMEC tube formation. Therefore, it is unlikely that soluble, cell culture media–associated factors contribute to the proangiogenic effect observed post-hESC-eEV treatment.

To assess the effect of hESC-eEVs on wound closure *in vitro*, we performed a wound healing assay using increasing concentrations of hESC-eEVs, with the maximum dose being 100 hESC-eEVs/cell (Figure 3). Our data demonstrated that the percentage of wound closure at 12–24h was significantly improved posttreatment with low hESC-eEV doses (<100 EVs/cell), whereas a higher hESC-eEV concentration (100 EVs/cell) or treatment with HUVEC hypoxic EVs was ineffective.

To evaluate whether the dose-response effect of the hESC-eEVs on tube formation and wound closure was specific to the H9 cell line, we performed EC differentiations in an independent, hESC line (RC11, which we used for prior cell therapy experiments[47]) and isolated eEVs (RC11-eEVs) at day 8 of differentiation (Figure S4). Furthermore, we have confirmed EV characteristics by means of NTA, TEM, and western blot. RC11-eEVs are approximately 130 nm, which aligns with previous findings in H9-derived eEVs (Figure S5A). Western blot analysis of RC11-eEVs confirms the presence of EV markers CD63 and CD9 whereas cellular marker calnexin was absent in EVs (Figure S5B). Morphological analysis of EVs by TEM also confirmed the typical phenotype of EVs displayed as spheric membrane-bound structures that are approximately 100 nm in size (Figure S5C). More important, RC11-eEVs showed a similar dose-response effect in tube formation as well as cell migration of HCMECs when compared to H9-derived eEVs (Figures S6 and S7, respectively).

Collectively, these results demonstrate the potency of hESC-eEVs to stimulate angiogenesis at low concentrations (<100 EVs/cell). Our data highlight that the proangiogenic potential is specific for EVs of the endothelial-enriched stage and not the mesodermal stage of the differentiation system.

hESC-eEVs are enriched in miRNAs with potentially novel proangiogenic function

Because the effect of EVs has been largely attributed to their small RNA cargo,[54] we aimed to identify RNA molecules responsible for the proangiogenic potential of hESC-eEVs. RNA was extracted from EVs from the following samples: hESC-eEVs, hESC-mEVs, and HUVEC hypoxic EVs (n = 3 independent differentiations) and we also obtained RNA from hESC-ECPs to identify miRNAs enriched in EVs over the cell donor cells. RNA size profiling showed the enrichment of small RNAs (25–200 nt) in EVs, and ribosomal and longer

Figure 2. EVs from hESC-eEVs induce EC tube formation at low concentrations

(A) Tube formation assay on HCMECs using increasing concentrations of HUVEC hypoxic EVs (dose range 1–10^5 EV/cell, n = 3). Cells cultured in basal media and treated with sterile filtered PBS (vehicle control) served as negative control (GF^-). Cells in fully supplemented media served as the positive control (GF^+). Graphs (right) represent the number of meshes and the total length of the tubes formed at 4 h. Images were analyzed using the angiogenesis analyzer tool on ImageJ. The control samples (GF^- and GF^+) are depicted as black circles, and the HUVEC hypoxic EV treated samples (HUVEC hyp EVs) are depicted as purple triangles. Statistical significance (indicated with p values) was determined by 1-way ANOVA with Dunnett's multiple comparisons test. Error bars represent the SD. Scale bar, 1mm. (B) Quantification of tube formation assay on HCMECs using increasing concentrations of hESC-eEVs (dose range 1–10^5 EV/cell) (n = 4). Cells cultured in basal media and treated with sterile 0.1 μm filtered PBS (vehicle control) served as negative control (GF^-). Cells in fully supplemented media (GF^+) or treated with 10^5 HUVEC hypoxic EVs/cell served as positive controls. Graphs represent the number of meshes and the total length of the tubes formed at 4 h. Data were analyzed using the "angiogenesis analyzer tool. The control samples (GF^- and GF^+) are depicted as black circles, the HUVEC hypoxic EV treated samples (HUVEC hyp EVs) are depicted as purple triangles, and the hESC-eEV-treated samples are depicted as green circles. Statistical significance (indicated with p values) was determined by 1-way ANOVA with Dunnett's multiple comparisons test. Error bars represent the SD. (C) Calcein-AM staining of tube formation assay on HCMECs using increasing concentrations of hESC-eEVs (dose range 1–10^5 EV/cell, n = 4). Cells cultured in basal media and treated with sterile 0.1 μm filtered PBS (vehicle control) served as negative control (GF^-). Cells in fully supplemented media (GF^+) or treated with 10^5 HUVEC hypoxic EVs/cell served as positive controls. Scale bar, 500 μm. (D) Tube formation assay on HCMECs treated with hESC-mEVs (dose range 1–10^5 EV/cell, n = 3). Cells cultured in basal media and treated with sterile filtered PBS (vehicle control) served as negative control (GF^-). Cells in fully supplemented media (GF^+) or treated with 1 hESC-eEV/cell served as the positive control. Images were analyzed using the angiogenesis analyzer tool on ImageJ. The control samples (GF^- and GF^+) are depicted as black circles, the hESC-eEV-treated samples are depicted as green circles, and the hESC-mEV-treated samples are presented as red triangles. Statistical significance (indicated with p values) was determined by 1-way ANOVA with Dunnett's multiple comparisons test. Graphs (right) represent the number of meshes and the total length of tubes formed at 4 h. Error bars represent the SD. Scale bar, 1mm. (E) Schematic representation of the samples collected at different stages of the particle isolation from hESC-EC cell culture media. Created with BioRender.com. (F) Tube formation assay using samples collected at different stages of the particle isolation from hESC-EC cell culture media. Cells cultured in basal media and treated with PBS (vehicle control) served as negative control (GF^-). Cells in fully supplemented media (GF^+) or treated with human recombinant $VEGFA_{165}$ or cultured with basal media and treated with 1 hESC-eEV/cell served as positive controls.

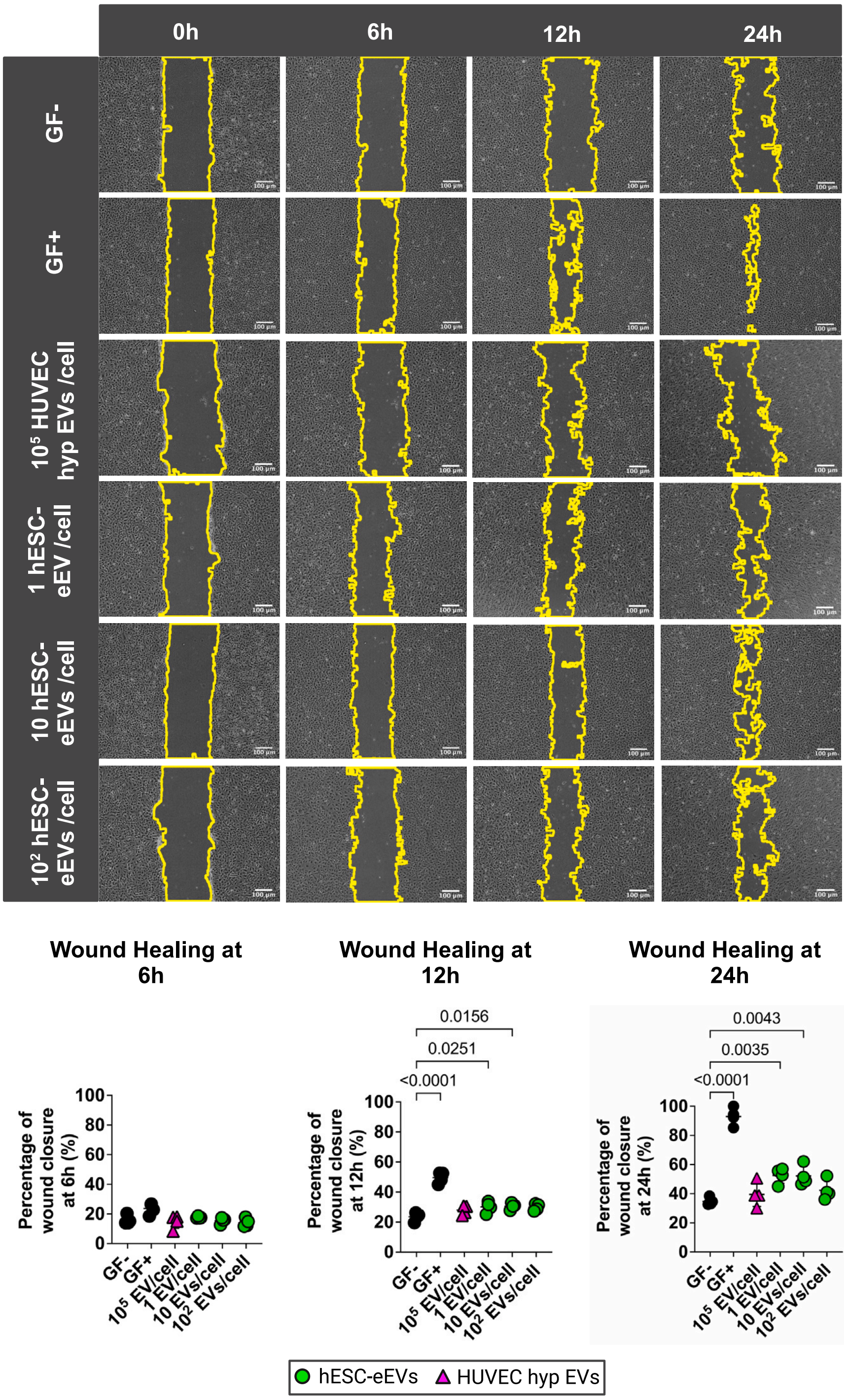

Figure 3. EVs from hESC-ECPs promote EC wound healing at low concentrations

Wound healing assay on HCMECs using increasing concentrations of EVs from hESC-eEVs (dose range 1–10^2 EV/cell) or treated with 10^5 HUVEC hypoxic EVs/cell (n = 4). Cells cultured in basal media and treated with sterile filtered PBS (vehicle control) served as negative control (GF$^-$). Cells in fully supplemented media (GF$^+$) served as positive controls. Graphs (below) represent the percentage of wound closure at 6, 12, and 24 h postwound induction. Data were analyzed using the MRI wound healing tool. The control samples (GF+ and GF−) are depicted as black circles, the HUVEC hypoxic EV treated samples (HUVEC hyp EVs) are depicted as purple triangles, and the hESC-eEV-treated samples are depicted as green circles. Statistical significance (indicated with p values) was determined by 1-way ANOVA with Dunnett's multiple comparisons test. Error bars represent the SD. Scale bar, 100 μm.

RNAs were virtually absent. Cellular RNA sample profiling showed two distinct ribosomal peaks corresponding to 18S and 28S for eukaryotic RNA (Figure S8). Small RNA sequencing (RNA-seq) revealed that the majority of cellular RNA sample reads mapped to miRNAs, whereas EV RNA samples showed a more diverse composition of small RNA classes, mapping mostly to tRNAs, miRNAs, miscellaneous RNAs, and protein-coding RNAs (Figure 4A). To understand whether EVs obtained from different conditions have distinct RNA molecules, we compared the list of unique miRNAs, Piwi interacting RNAs (piRNAs), tRNAs, and other RNAs between EV types (Figure S9). tRNAs and piRNAs were less variable, and miRNAs and other small RNAs were the most heterogeneous populations among the EV samples. Overall, the different EV samples shared a total of 72 miRNAs and 172 other small RNAs. In the present study, we focused on miRNAs for further investigation because EV-miRNAs have a well-established role in the regulation of angiogenesis[7] in CVDs.

We next investigated the presence of EC-enriched miRNAs (miR-126-5p, miR-222-3p, miR-99b-5p, miR-22a-3p)[55] in our datasets and confirmed high abundance in EVs of endothelial-enriched or endothelial origin (hESC-eEVs and HUVEC hypoxic EVs) and not in hESC-mEVs (Figure S10). In line with previous findings on cellular RNA composition reporting that for any human cell type the most abundant 3–5 miRNAs occupy more than 50% of the total miRNA pool,[55] we noted that the top 3 miRNAs in hESC-ECPs corresponded to more than 50% of the total miRNA reads. A similar profile was observed in the EV samples where the top 5 EV miRNAs corresponded to approximately 50% of the total miRNA reads (Figure S11). The top 5 miRNAs in hESC-eEVs contain molecules extensively studied for their roles in driving angiogenesis, whereas most of the top 5 miRNAs in hESC-mEVs have an antiangiogenic action.[12,56–65] A literature search of

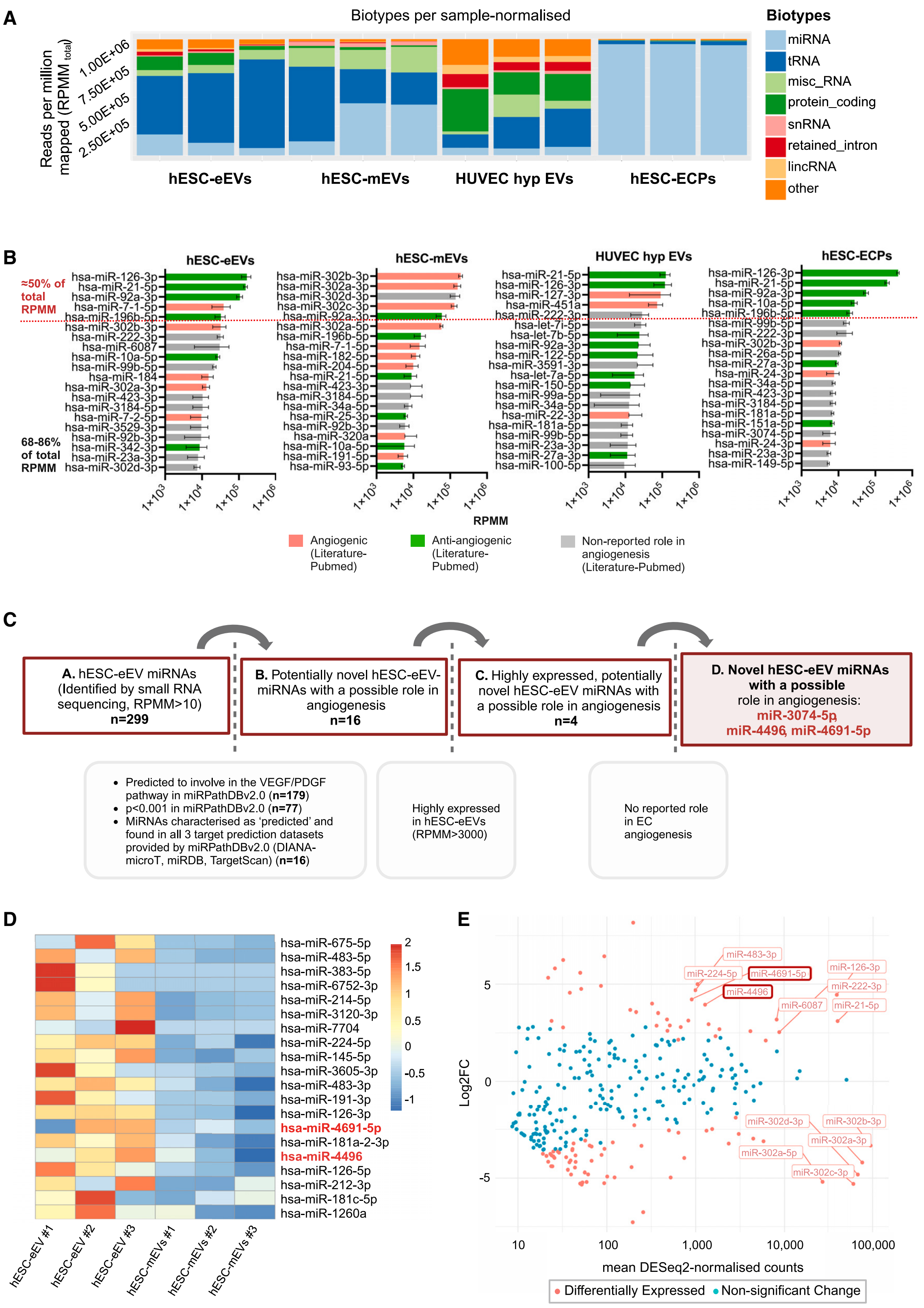

(legend on next page)

the 20 most abundant miRNAs of each sample (that corresponded to 68%–86% of the total reads) showed the presence of several miRNAs with nonreported roles in angiogenesis in hESC-eEVs (Figure 4B).[12,49–58]

To rule out that the EV miRNA cargo is cell line specific, we have also compared the miRNA cargo of RC11-eEVs with H9-derived eEVs (hESC-eEVs) by means of small RNA-seq (Table S1). From the top 20 miRNAs, 65% are shared between RC11-eEVs and hESC-eEVs and include well-described angiogenic miRNAs (Figure S12). Only 9.9% of the less-abundant RC11-eEV miRNAs are shared. Therefore, the top miRNAs in RC11-eEVs are largely concordant with those in hESC-eEVs, emphasizing the conserved nature of miRNA cargo across EVs from ECPs from different hESC sources.

Due to the limitations of the literature search in providing a comprehensive view of the angiogenic potential of all miRNAs, we further identified hESC-eV-miRNAs with potentially novel angiogenic functions by performing an unbiased *in silico* screen (Figure 4C). We found a total of 179 out of the 299 hESC-eEV-miRNAs (Table S7) being predicted or experimentally validated to involve in angiogenesis pathways. Of those, our analysis highlighted a total of 3 miRNAs, also belonging in the top 50 hESC-eEV miRNAs (miR-3074-5p, miR-4496, miR-4691-5p), as hESC-eEV-miRNAs with potentially novel angiogenic function. Thus, our small RNA-seq and *in silico* screen indicated that hESC-eEVs are enriched in proangiogenic, as well as miRNAs with potentially novel angiogenic function.

Aiming to identify miRNA molecules responsible for the strong proangiogenic potential of hESC-eEVs compared to hESC-mEVs, we performed a differential expression analysis using DESeq2.[66] Our analysis suggested that hESC-eEVs and hESC-mEVs have divergent transcriptomic profiles that may help explain the different angiogenic profiles (Figure S13). Overall, 145 miRNAs were found to be differentially expressed between hESC-eEVs and hESC-mEVs (Table S1). A total of 5 out of the 20 most differentially expressed miRNAs between hESC-eEVs and hESC-mEVs (Figure 4D) had particularly high expression (reads per million mapped [RPMM] >3,000) in hESC-eEVs (Table S1). Of those, the most abundant miRNA in hESC-eEVs was the EC-enriched[55] miRNA miR-126-3p, whose role in driving angiogenesis has been extensively studied.[56–59] This list also contained the novel hESC-eEV-miRNAs, with a potential role in angiogenesis miR-4496 and miR-4691-5p. Other molecules in this list were miRNAs with no reported roles in angiogenesis (miR-224-5p) and a miRNA with antiangiogenic function (miR-483-3p).[67] We also noted that compared to hESC-mEVs, hESC-eEVs were depleted of miRNAs of the miR-302 family that regulate the transition of naive to primed pluripotency[68] and are reported to inhibit angiogenesis (Figure 4E).[64,69] Interestingly, the two differentially expressed miRNAs (hsa-miR-4496 and hsa-mir-4691-5p) with potentially novel roles in angiogenesis are not conserved between humans and mice/rats. This observation aligns with our broader analysis, which revealed that only 29% of the total hESC-eEV miRNAs show confirmed conservation between humans and mice/rats (Figure S14).

Collectively, our analysis suggests that hESC-eEVs are enriched in miRNAs with well-established roles in driving angiogenesis and in miR-4496 and miR-4691-5p with potentially novel angiogenic function. We confirmed that the observed proangiogenic effects of eEVs at low concentrations as well as the eEV miRNA cargo are largely replicated in the RC11-hESC line.

miR-4496 and miR-4691-5p are novel EV-associated miRNAs with novel proangiogenic function

Next, we aimed to investigate whether hESC-eEVs carry novel EV-miRNAs—thus, miRNAs that have not been reported in previous EV small RNA-seq studies. To identify potentially novel EV-associated miRNAs in hESC-eEVs, we compared the list of miRNAs present in hESC-eEVs (RPMM >10) to the curated lists of previously identified EV-associated miRNAs reported in Vesiclepedia and ExoCarta databases (Figure 5A). This comparison yielded a total of 24 miRNAs that were absent in previously profiled EVs. Of the 24 miRNAs identified, miR-4496 and miR-4691-5p had a particularly high expression in hESC-eEVs (RPMM >3,000). This suggests that miR-4496 and miR-4691-5p may be enriched in hESC-eEVs.

Because EV-sorted molecules play key roles in intercellular communication and control important biological processes, including angiogenesis,[7] we performed a differential expression analysis between hESC-eEVs and the EV donor cells to identify potentially EV-targeted miRNAs. A total of 59 miRNAs were enriched in hESC-eEV relative to hESC-ECPs, suggesting that these may be actively/selectively imported in EVs (Table S1). miR-4496 was the most enriched miRNA in hESC-eEVs (Figure 5B) while also being the third most abundant in the list of the five miRNAs with the highest fold change between hESC-eEVs and hESC-ECPs (Figure 5C). miR-4691-5p was also

Figure 4. EVs from hESC-ECPs are enriched in proangiogenic and the novel miRNAs with a potential role in angiogenesis miR-4496 and miR-4691-5p
(A) Graph showing the RPMM of small RNA biotypes in the EV samples and hESC-ECPs (n = 3) normalized to the total number of small RNA reads ($RPMM_{total}$). (B) Plots representing the RPMM of the top 20 miRNAs in each group (corresponding to 68%–86% of total RPMM) and their role in angiogenesis. The top 5 miRNAs in each group are separated by a dotted vertical red line. Angiogenic miRNAs are highlighted in green and antiangiogenic miRNAs are highlighted in red. The literature search was conducted in PubMed in July 2022. (C) Workflow of *in silico* analysis identifying 3 novel hESC-eEV-miRNAs with a possible role in angiogenesis. (D) Heatmaps representing the top 20 most differentially expressed miRNAs among the angiogenic hESC-eEVs and nonangiogenic hESC-mEVs as *Z* score of log2(DESeq normalized counts + 1), LFC >1, $p < 0.005$. miRNAs are ranked from the highest to lowest fold change. miRNAs with a potentially novel angiogenic function are in red. (E) Plot representing for hESC-eEV-miRNAs: log2fold change versus expression in hESC-mEVs (y axis) and mean DESeq normalized counts (x axis). Significantly differentially expressed miRNAs are highlighted in orange ($p < 0.05$, $|log2FC| = 1$). miRNAs with normalized counts over 1,000 and a particularly high fold change are labeled. miRNAs belonging in the list of novel hESC-eEV-miRNAs with a potential role in angiogenesis are bold red shapes.

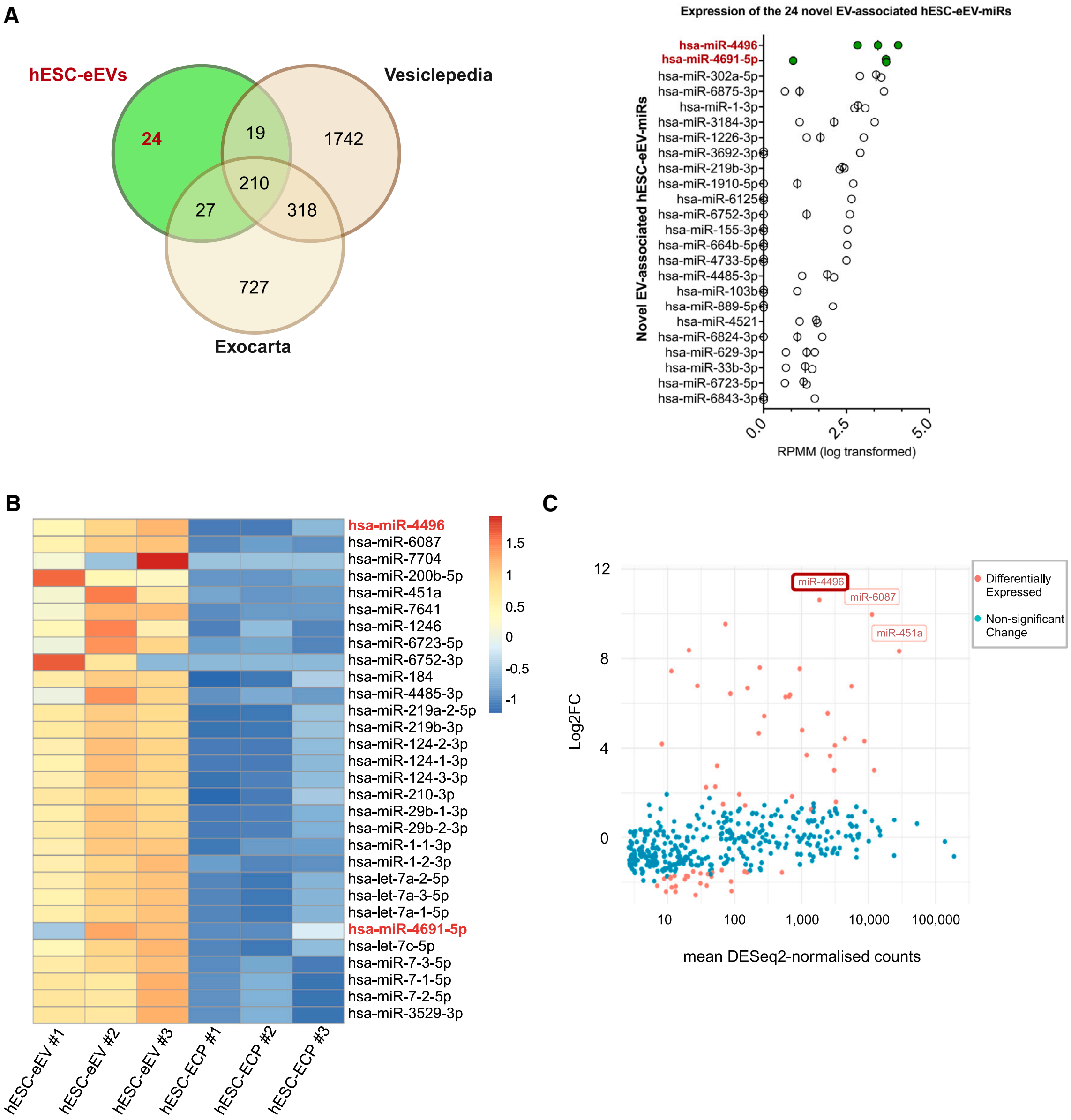

Figure 5. MiR-4496 and miR-4691-5p are novel EV-associated miRNAs and enriched in EVs from hESC-ECPs compared to the EV donor cells

(A) Diagram (left) comparing the miRNA cargo of EVs from hESC-eEVs with lists of all of the known EV-associated miRNAs reported in Vesiclepedia and Exocarta (search conducted in July 2022). Molecules with average RPMM <10 were filtered out of the analysis. Graph (right) representing the log(RPMM + 1) of the 24 novel EV-associated miRNAs present in the hESC-eEVs. miRNAs are ranked from the highest to lowest RPMM. miRNAs in the list of miRNAs with a potentially novel angiogenic function are in bold red. miRNAs with average RPMM >3,000 are represented by green points. (B) Heatmaps representing the top 30 most differentially expressed miRNAs among hESC-eEVs and hESC-ECPs as Z score of log2(DESeq normalized counts + 1), LFC >1, $p < 0.05$. miRNAs are ranked from the highest to lowest fold change. miRNAs in the list of miRNAs with a potentially novel angiogenic function are in red. (C) Plot representing for hESC-eEV-miRNAs: log2fold change versus expression in hESC-ECPs (y axis) and mean DESeq normalized counts (x axis). Significantly differentially expressed miRNAs are highlighted in orange ($p < 0.05$, |log2FC| = 1). miRNAs with normalized counts over 1,000 and a particularly high fold change are labeled. miRNAs belonging in the list of novel hESC-eEV miRNAs with a potential role in angiogenesis are in bold red.

significantly enriched in hESC-eEVs compared to hESC-ECPs. We noted that 17 of the 59 hESC-eEV miRNAs have low or no expression in hESC-ECPs (RPMM <10), suggesting a function specifically carried out via EVs (Table S1). Of those, the 2 most abundant genes and highly expressed in hESC-eEVs (RPMM >3,000) were miR-4496 and miR-4691-5p, highlighted already for their potential specificity to hESC-eEVs. miRNA sorting in EVs may be facilitated by the presence of specific motifs in the miRNA sequence.[21–25] Interestingly, hsa-miR-4496 contained the EV-sorting motif GAGG and miR-4691-5p contained the CAUG and AGGCC EV-sorting motifs previously

reported by Garcia-Martin et al.[25] (Figure S15). These data indicate that miR-4496 and miR-4691-5p are enriched in hESC-eEVs compared to the EV donor cells, possibly due to the presence of EV-sorting motifs in their sequence.

Our analysis highlighted miR-4496 and miR-4691-5p as novel EV-associated candidates with potentially novel angiogenic function, enriched in the proangiogenic hESC-eEVs compared to the nonangiogenic hESC-mEVs or the EV donor cells. Collectively, these data suggest important biological functions and a possible role in driving angiogenesis. Thus, to experimentally examine the potential proangiogenic role of miR-4496 and miR-4691-5p, we performed tube formation and wound healing assays on HCMECs overexpressing each miRNA (Figure 6). Because the role of miR-126-5p in angiogenesis is extensively studied,[56–59] cells overexpressing miR-126-5p were used as positive controls of angiogenic cells. Cells transfected with a miRNA control (miRC) were used as negative controls. miRNA overexpression was confirmed by qPCR (Figure S16). Using a tube formation assay, we showed that both miRNAs significantly increased the number of meshes and total length of the tubes formed both in basal conditions and when cells were cultured in fully supplemented media (Figure 6A). By wound healing assay, we demonstrated that 24 h after the induction of the wound, HCMECs transfected with either miR-4496 or miR-4691-5p had significantly improved wound closure both in basal conditions ($p < 0.001$) and in fully supplemented media ($p = 0.0079$ and $p = 0.0099$, respectively) (Figure 6B). Thus, our data validate miR-4496 and miR-4691-5p as miRNAs with novel proangiogenic function.

DISCUSSION

In this study, our aim was to investigate the effect of hESC-eEVs on angiogenesis, building upon our previous data suggesting the involvement of paracrine mechanisms in the observed improvements following hESC-ECP transplantation.[47] The present study provides several novel findings in the field. We established a reproducible protocol for the isolation of pure and biologically active EVs from hESC-ECP cultures. We demonstrated that compared to other EC-derived EVs, hESC-eEVs induce proangiogenic responses at low concentrations. Notably, EVs isolated from more immature stages of the differentiation protocol did not exhibit proangiogenic activity. We also demonstrated that hESC-eEVs are enriched in proangiogenic and miRNAs with potentially novel angiogenic function. Moreover, we report the presence of several novel EV-associated miRNAs in hESC-eEVs, which will further expand the list of miRNAs known to be transferred through EVs to date.

To gain further insight into the molecular composition of hESC-eEVs, we performed small RNA-seq. Despite some limitations, such as the low RNA input in the EV samples,[6] which we found to correlate to a high percentage of unmapped reads, our analysis provided sufficient coverage to profile the most highly abundant miRNAs, which are those most likely to induce angiogenic responses at the low concentrations required for hESC-eEVs. Our analysis revealed for the first time that in line with previous observations regarding the RNA composition of human cell types, where a few miRNAs make up the majority of the total miRNA pool,[55] the top 5 miRNAs of our EV datasets also accounted for approximately 50% of the total miRNA reads. The most abundant miRNAs in hESC-eEVs are well-established proangiogenic miRNAs, which indicates why a low dose of hESC-eEVs is sufficient to induce angiogenic responses. Moreover, we identified and experimentally validated two novel angiogenic and novel EV-associated, hESC-eEV-enriched miRNAs, miR-4496 and miR-4691-5p. These findings highlight the unique repertoire of miRNAs within hESC-eEVs and their potential contributions to the observed effect.[25]

Although small RNA-seq analysis highlighted the presence of proangiogenic miRNAs, our data suggest that achieving a precise stoichiometry is crucial for exerting the therapeutic function of hESC-eEVs. It is unclear whether the loss of the proangiogenic effect at high hESC-eEV concentrations can be explained by the hESC-eEV-miRNA cargo alone. Because antiangiogenic miRNAs were also identified in hESC-eEVs, the relative abundance and distinct dose-response curves of pro- and antiangiogenic miRNAs, as well as additional molecules within the hESC-eEVs cargo, such as other small RNAs or proteins, could explain the observed effects. In fact, similar dose-dependent effects have been observed with certain proteins, including transforming growth factor-β1 and p43.[70,71]

Although our study primarily focuses on harnessing the potent angiogenic effect at low doses, further comprehensive analysis of the cargo within hESC-eEVs may provide a deeper insight into the observed dose-dependent effects. In addition, because the use of *in vitro* assays to assess angiogenesis may not fully recapitulate the complex processes that occur *in vivo*, future studies should assess the efficacy of hESC-eEVs in promoting angiogenesis in preclinical models or clinical trials. Our findings indicate that only 29% of the eEV miRNA repertoire is conserved between humans, and the most commonly utilized *in vivo* model organisms, mice and rats. Given these circumstances, attempting to evaluate the performance of both the EVs and the miRNAs in an *in vivo* setting would be therefore suboptimal and difficult to interpret. For follow-up studies, however, it would be interesting to test eEVs in a human heart-on-a-chip model system or human cardiac explant tissues.[72]

From a translational perspective, EVs are advantageous systems for the delivery of angiogenic cargo due to their innate ability to be taken up by cells or tissues and their role as natural mediators of intercellular communication.[73] EVs derived from stem cells (SCs) or ECs have been demonstrated to promote cardiac angiogenesis and vascular regeneration in MI by transferring angiogenic miRNAs.[74] EV-based therapeutics, however, encounter challenges such as large-scale production, standardized isolation methods, and high cargo-loading efficiency.[75] Synthetic lipid-based delivery systems, such as lipid nanoparticles (LNPs), have shown promise in addressing some of these limitations. However, apart from immunogenicity issues and challenges concerning the stability and efficient delivery of LNPs to specific target sites within the body,[73] there is also the

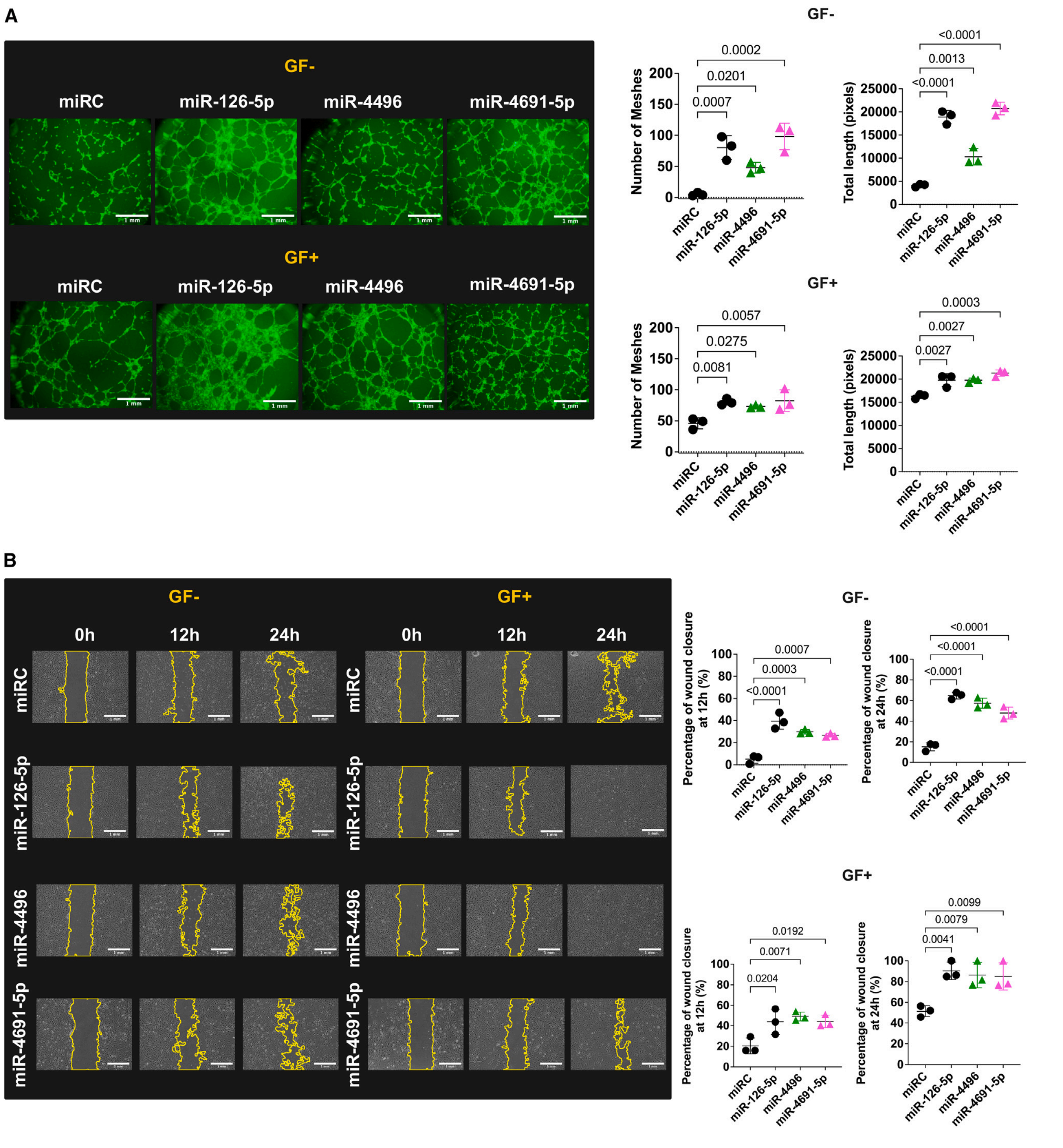

Figure 6. Overexpression of miR-4496 or miR-4691-5p results in increased EC tube formation and wound healing

(A) Tube formation assay on HCMECs transfected with miR-4496 or miR-4691-5p (n = 3). Cells transfected with miR-126-5p served as the positive control. Cells transfected with a miRNA control (miRC) served as the negative control. Staining with calcein-AM confirmed cell viability in all conditions. Experiments were performed both in basal conditions (GF^{-}) and in fully supplemented media (GF^{+}). Data were analyzed using the angiogenesis analyzer tool. Statistical significance (indicated with p values) was determined by 1-way ANOVA with Dunnett's multiple comparisons test. Error bars represent the SD. Scale bars, 100 μm. (B) Wound healing assay on HCMECs transfected with miR-4496 or miR-4691-5p (n = 3). Cells transfected with miR-126-5p served as the positive control. Cells transfected with an miRC served as the negative control. Experiments were performed both in basal conditions (GF^{-}) and in fully supplemented media (GF^{+}). Data were analyzed using the MRI wound healing tool. Statistical significance (indicated with p values) was determined by 1-way ANOVA with Dunnett's multiple comparisons test. Error bars represent the SD. Scale bars, 100 μm.

requirement of much higher doses for the delivery of functional RNA molecules compared to EVs.[76] Moreover, because it was recently shown that LNP-based delivery of vascularendothelialgrowthfactor A (VEGFA) is functionally extended by the target cells' own EVs, potential secondary paracrine effects of LNP-based therapeutics should also be considered.[77] Ongoing research aims to address the challenges of both EVs and LNPs for clinical applications.

One limitation of this study is that the exact mechanism underlying the proangiogenic effect of hESC-eEVs was not explored in depth. Future studies could investigate this mechanism to gain a better understanding of how hESC-eEVs promote angiogenesis and identify potential targets for therapeutic intervention. In addition, the use of *in vitro* assays to assess angiogenesis may not fully recapitulate the complex processes that occur *in vivo*. Therefore, future studies should also evaluate the efficacy of hESC-eEVs in promoting angiogenesis in animal models or clinical trials.

Overall, the present study is the first to investigate the effect of hESC-eEVs in angiogenesis and to provide a comprehensive insight into the small RNA content of hESC-eEVs. Our data highlight the following elements that will contribute to both the EV and the CVD field: (1) in contrast to other EC-derived EVs, hESC-eEVs induce angiogenic responses at low concentrations; (2) hESC-eEVs are enriched in proangiogenic miRNAs; (3) hESC-eEVs contain novel EV-associated miRNAs, with the most highly expressed being miR-4496 and miR-4691-5p; (4) miR-4496 and miR-4691-5p highly enriched in hESC-eEVs have novel proangiogenic function. All of these findings highlight the potential use of hESC-eEVs in regenerative medicine, as well as the ability to harness their proangiogenic potential to identify novel small RNA-based therapeutics.

MATERIALS AND METHODS

hESC-ECP differentiation

H9[78] and RC11[79] hESC lines were differentiated to endothelial progenitor cells using our previously reported protocol.[47] hESC lines were used in accordance with the UK Stem Cell Bank Steering Committee guidelines (Project Approvals SCS11-51 and SCSC17-26). Briefly, hESCs were plated on a fibronectin matrix at day 0 (d0). At d1, lateral mesoderm was induced with GSK3 inhibitor (CHIR99021) (7 μM) and BMP4 (25 ng/mL) added to N2B27/Neurobasal/DMEM: F12 media. This was followed by EC induction at d4 with forskolin (2 μM) and VEGF (200 ng/mL) in StemPro34 media. In the final step, cells were replated and cultured until d8. For EV isolation, media was replaced with EV-free media on d7, and conditioned media was collected after 24 h. To prepare EV-free media, human serum was ultrafiltrated using Amicon-Ultra 15 Centrifugal Filter Units (no. UFC9100, Merck Millipore) with a cutoff of 100 kDa after centrifugation at 3,000 × *g* for 55 min (4°C) and supplemented to the cell culture media.

Flow cytometry

On day 8 of the differentiation protocol, hESC-ECPs were stained using antibodies (given in Table S2) to determine the proportion of pluripotent (SSEA-4^+/TRA-181^+) and endothelial ($CD31^+$/$CD144^+$) cells within the population. Flow cytometry was conducted with the BD LDR Fortessa system (Becton Dickinson) or the Attune NxT system (Thermo Fisher Scientific) and data were analyzed using the FlowJo software (FlowJo, Ashland, OR).

EV isolation

To isolate EVs conditioned media was obtained from cells at 70%–90% confluency and centrifuged at 3,000 × *g* for 15 min (4°C) to remove cell debris. Supernatants were collected and concentrated to 1 mL using Amicon-Ultra 15 Centrifugal Filter Units (no. UFC9100, Merck Millipore) with a cutoff of 100 kDa by centrifugation at 4,000 × *g* for 15 min (4°C). Cell counting was performed post-media collection (each T75 contained 2.4 × 10^6 cells (n = 3; Table S3), and cell viability was measured by trypan blue staining. The concentrated samples were loaded onto 15-mL sepharose CL-6B (no. CL6B200, Sigma-Aldrich) SEC columns. Once the whole sample had entered the column matrix, 0.1 μm filtered Dulbecco's PBS (no. 11340972, Gibco) supplemented with 1% penicillin/streptomycin (no. 15140122, Gibco) was continuously added to ensure complete drainage of the sample. The eluate was collected by gravity in 15 sequential fractions of 1 mL; for each fraction, the amount of protein was determined by spectrophotometry (Absorbance 280 nm, Nanodrop, Thermo Fisher Scientific). To select fractions enriched for EVs and devoid of protein contamination, the protein and EV concentration of all of the fractions was measured by spectrophotometry and NTA, respectively. Fractions 5 and 6 containing considerably higher numbers of particles and low protein concentrations were pooled and used for downstream applications (Figure S17).

NTA

Particle concentration and size distribution were determined using NanoSight LM 10 instrument (Malvern Panalytical). Samples described above were diluted 10–40 times in 0.1 μm of filtered Dulbecco's PBS (no. 11340972, Gibco) supplemented with 1% penicillin/streptomycin (no. 15140122, Gibco) to obtain a concentration of 2 × 10^8–1 × 10^9 particles/mL. Camera screen gain was set to 2 and the camera level was set to 16. The settings were kept constant between samples, and each video was analyzed to give the mean, mode, median, and estimated concentration for each particle size. The analysis was carried out with the NTA software (version 3.3) using 60 s of video captures per sample (five replicates per sample; Video S1). For the video process, the screen gain was set to 14 and the detection threshold was set to 3.

Western blot

EV-enriched fractions were concentrated using Amicon-Ultra 15 Centrifugal Filter Units (no. UFC9100, Merck Millipore) with a cutoff of 100 kDa by centrifugation at 4,000 × *g* for 15 min (4°C). For the positive control, cells were lysed in a radioimmunoprecipitation assay lysis buffer (50 mM Tris HCl, pH 8, 1% NP-40, 0.2% sodium deoxycholate, 150 mM NaCl, 1% Triton X-100, 0.1% SDS) supplemented with protease inhibitors (Roche Diagnostics) and centrifuged at 12,000 × *g* for 10 min at 4°C. The supernatants were collected, and

the protein concentration was determined with the Pierce BCA Protein Assay Kit (no. 23227, Thermo Fisher Scientific). A total of 5×10^{10} particles and 15 mg proteins of cell lysates were separated by NuPAGE 4%–12% Bis-Tris polyacrylamide gels (no. NP0321BOX, Thermo Fisher Scientific) and electrophoretically transferred to nitrocellulose membranes (Invitrogen). The membranes were blocked with fish serum blocking buffer (no. 37527, Thermo Fisher Scientific) at room temperature for 1 h and incubated overnight at 4°C with primary antibodies. Membranes were then washed with Tris-buffered saline with 0.1% Tween 20 (0.5% Tween here) detergent (TBS-T), pH 7.5, 3 times for 5 min and incubated with secondary antibodies for 45 min. Details on the primary and secondary antibodies, including reference numbers, host species, and dilutions are provided in Table S4. Membranes were then washed again with TBS-T (0.5% Tween here), pH 7.5, 3 times for 5 min and stored in PBS at 4°C. Proteins were visualized by scanning the membrane on an Odyssey Infrared Imaging System (LI-COR Biosciences) with both 700- and 800-nm channels.

TEM

Isolated particles were diluted 100 times in 0.1 μm filtered Dulbecco's PBS (no. 11340972, Gibco) and mixed 1:1 with 4% methanol-free formaldehyde solution (no. 28906, Thermo Fisher Scientific). A drop of this solution was placed on a Petri dish, and then a Formvar-coated 200-mesh gold grid (Taab, Aldermaston, UK) floated on top for 20 min. The grid was moved to PBS for two 5-min washes. Then, the EVs were refixed on the grid using a 1% glutaraldehyde solution for 5 min and again washed twice in PBS. Finally, the grid was transferred to a drop of 0.5% uranyl acetate–2% 25-centipoise methyl cellulose (Sigma Aldrich). Following staining for 5 min, the excess fluid was removed, and the grid was allowed to air dry and was then examined on a JEOL JEM-1400 series 120kV transmission electron microscope. The TEM pictures were analyzed using the TEM exosome analyzer[80] plugin on ImageJ (NIH).[81]

EC culture

HCMECs and HUVECs were obtained from PromoCell. HCMECs were cultured at 37°C in a humidified atmosphere with 5% CO_2 and 95% O in MV2 media (no. C-22022, PromoCell) supplemented with 5% fetal calf serum (FCS), 5 ng/mL epidermal growth factor (recombinant human), 10 ng/mL basic fibroblast growth factor (recombinant human), 20 ng/mL insulin-like growth factor (Long R3 IGF), 0.5 ng/mL $VEGF_{165}$ (recombinant human), 1 μg/mL ascorbic acid, and 0.2 μg/mL hydrocortisone. HUVECs were cultured in EBM2 media (#00190860, Lonza) supplemented with 2% FCS, 5 ng/mL epidermal growth factor (recombinant human), 10 ng/mL basic fibroblast growth factor (recombinant human), 20 ng/mL IGF (Long R3 IGF), 0.5 ng/mL $VEGF_{165}$ (recombinant human), 1 μg/mL ascorbic acid, 22.5 μg/mL heparin, and 0.2 μg/mL hydrocortisone. To isolate EVs from hypoxic HUVECs, when cells reached confluency, media was replaced with EV-free media and transferred to a sealed chamber with 5% CO_2, 92% N_2, and 3% O_2. To prepare EV-free media, FBS was ultrafiltrated using Amicon-Ultra 15 centrifugal filter units (no. UFC9100, Merck Millipore) with a cutoff of 100 kDa after centrifugation at 3,000 × *g* for 55 min (4°C) and supplemented to the cell culture media.

miRNA overexpression

All of the miRNA overexpression experiments were conducted using miRIDIAN miRNA mimics (Horizon Discovery). An appropriate miRC (Horizon Discovery) was used in parallel with all of the miRNA overexpression experiments (Table S5). miRC is based on the cel-miR-67 mature sequence and has been confirmed to have minimal sequence identity with human, mouse, and rat miRNAs. Transient transfection was performed using Lipofectamine RNAiMAX transfection reagent (no. 13778100, Thermo Fisher Scientific), according to the manufacturer's instructions, supplemented with 25 nM of either miRNA precursor. At 24 h posttransfection, the transfection medium was removed, and the cell culture was maintained using fully supplemented media for up to 72 h.

miRNA reverse transcription and qRT-PCR

For miRNA analysis upon miRNA overexpression in HCMECs, cDNA was generated from total RNA (2 ng/reaction) using the TaqMan miRNA Reverse Transcription kit (no. 4366596, Thermo Fisher Scientific) and specific TaqMan miRNA reverse transcription probes (Table S6). Synthesis was performed by subjecting each sample to 30 min at 16°C for primer annealing, 30 min at 42°C to allow for reverse transcription, and 5 min at 85°C for RT inactivation. After synthesis, all of the samples were stored at −20°C until required. Note that, RNU48 was selected as an endogenous control miRNA to allow for the normalization of changes in miRNA expression.

miRNA qRT-PCR was performed using specific TaqMan miRNA RT-PCR probes (Table S6) and TaqMan Universal Master Mix II (no. 4440040, Thermo Fisher Scientific) following the manufacturer's protocol. Each reaction was conducted in triplicate using a QuantStudio 5 Real-Time PCR System (Thermo Fisher Scientific), subject to 10 min at 95°C followed by 40 cycles of 15 s at 95°C and 60 s at 60°C for primer annealing and extension. All qRT-PCR data obtained were analyzed using the $2^{-\Delta\Delta Ct}$ method, as described by Livak and Schmittgen.[82] The $2^{-\Delta\Delta Ct}$ values were used to calculate the fold change (described as relative quantification) in gene expression between the experimental and control groups.

Tube formation assay

The angiogenic effect of hESC-eEVs, hESC-mEVs, RC11-eEVs, and HUVEC hypoxic EVs on HCMECs was assessed by the tube formation assay. Cells were stained with 2 μM/mL calcein-AM (no. C1430, Thermo Fisher Scientific) after a 30-min incubation at 37°C. Growth factor-reduced ECM gel (no. E6909, Sigma-Aldrich) was thawed on ice at 4°C overnight, and 10 μL was plated per well onto an μ-plate angiogenesis 96-well (no. IB-89646, Thistle Scientific). After a 30-min gelation period at 37°C, 1.5×10^4 HCMECs were seeded onto each well. HCMECs were incubated either with fully supplemented or with basal media with Dulbecco's PBS + penicillin/streptomycin, or with basal media containing increasing concentrations of EVs ($1–10^5$ EVs/cell). Bright-field micrographs at 4× were

obtained using the EVOS XL Imaging System (Invitrogen). The number of meshes and branches and the total tube length was analyzed using the angiogenesis analyzer plug-in (Gilles Carpentier)[83] on ImageJ software.[81]

The angiogenic effect of miR-4496 and miR-4691-5p was also assessed by tube formation assay. Briefly, 48-h posttransfections with miR-4496 or miR-4691-5p, or miR-126-5p, or miRC cells were stained with 2 μM/mL calcein-AM (no. C1430, Thermo Fisher Scientific) after a 30-min incubation at 37°C. Tube formation assays were performed as described above. HCMECs overexpressing the miRNAs of interest or miRC were cultured both in basal and fully supplemented media to identify proangiogenic and antiangiogenic miRNAs, respectively.

Wound healing assay

The ability of hESC-eEVs and RC11-eEVs to promote wound healing *in vitro* was assessed by the wound healing assay. HCMECs were seeded in 6-well plates to create a confluent monolayer. Once they reached 70%–90% confluency, the cell monolayer was scratched with a sterile P200 pipette tip and media was replaced to fully supplemented, or with basal media with Dulbecco's PBS + penicillin/streptomycin, or with basal media containing increasing concentrations of hESC-eEVs ($1–10^2$ EVs/cell) or 10^5 HUVEC hypoxic EVs/cell for 24 h. Images were acquired using a bright-field microscope at 0-, 6-, 12-, and 24-h postscratch induction. The percentage of wound coverage, taking the value at 0 h as 0%, was calculated using the MRI Wound Healing Tool[84] (Montpellier Resources) on ImageJ software.[81]

We also tested the ability of miR-4496 and miR-4691-5p to promote wound healing *in vitro*. Experiments were performed 48 h posttransfections with miR-4496 or miR-4691-5p, or miR-126-5p, or miRC cells. HCMECs overexpressing the miRNAs of interest or miRC were cultured both in basal and fully supplemented media to identify proangiogenic and antiangiogenic miRNAs, respectively. Images were acquired using a bright-field microscope at 0-, 12-, and 24-h postscratch induction.

RNA extraction

Total cellular RNA was extracted using a miRNeasy Mini Kit (no. 217084, Qiagen) according to the manufacturer's instructions. The total exosome RNA and protein isolation kit (no. 4478545, Thermo Fisher Scientific) was used for the recovery of RNA from the EV samples according to the manufacturer's instructions. RNA quantity and quality were further analyzed by the Agilent 2100 Bioanalyzer system (Agilent Technologies) using the total RNA Pico Series II chip (no. 5067-1513, Agilent Technologies).

Small RNA-seq

EV isolation by a combination of ultrafiltration with SEC results in high-purity EV preparations but with a lower yield than other methods.[6] To counter the resulting lower input for our libraries, we provided the maximum possible input for sequencing each EV library with the minimum total RNA quantity being 1 ng (Table S3) (n = 3). Small RNAs were selected by 15% urea-PAGE and gel extraction between 18 and 30 nt. After gel purification, ligation of the adenylated 3′ adapter to the small RNA fragments was performed. Reverse transcription primers with barcodes were used to anneal the 3′ adenylated adapter to combine the redundant unligated 3′ adenylated adapter. Then, ligation of the 5′ adapter and reverse transcription reactions were performed. After cDNA first-strand synthesis, the product was amplified by 15 cycles. A second size selection of 103- to 115-bp fragments from the gel was carried out. This step was conducted to purify the PCR product and remove any nonspecific products. After gel purification, the PCR yield was quantified by Qubit (no. Q33216, Thermo Fisher Scientific). Samples were pooled together to make a single-strand DNA (ssDNA) circle, which produced the final small RNA library. DNA nanoballs (DNBs) were generated with the ssDNA circle by rolling circle replication to enlarge the fluorescent signals at the sequencing process. The DNBs were loaded into the patterned nanoarrays, and single-end reads of 50 bp were read through on the BGISEQ-500 platform for the following data analysis study (sequencing depth ≥ 20 million reads per sample).

Sequencing read mapping and small RNA annotation

The raw data from the BGI-SEQ500 were in fastq format. We used two mapping and quantification approaches to ensure a robust analysis of EV miRNA profiles, the first of which used the excerpt small RNA-seq processing pipeline.[85] Files were exported to the Genboree Workbench's excerpt small RNA-seq pipeline (version 4.6.2) for quality control as well as read mapping to the human genome and default small RNA libraries: miRNAs, tRNAs, piRNAs, and other small RNAs (including miscellaneous RNA, small nuclear RNA, yRNA, retained introns, protein-coding molecules, and long intergenicnoncoding RNA). This allowed for a single-mismatched base down to 18 nt. Reads unsuccessfully clipped (0%), failing quality filter (<1.2%), UniVec contaminants (<0.6%), and rRNA (5–51.4%) were filtered out of the analysis. After these initial filters, the average reads used for alignment for each group ranged from 24.7 to 28.3 million reads (Figure S18A). A high percentage of reads could not be aligned to the genome or small RNA libraries and was likely a result of background nonspecific amplification in lower input libraries (particularly in hESC-eEVs and HUVEC hypoxic EVs, where 30.4%–78% of the reads used for alignment could not be mapped) (Figure S18B). We found a negative correlation (rho −0.7, $p < 0.05$, Spearman's rank) between the percentage of unmapped reads and the number of particles used for RNA extraction (as measured by NTA and Agilent Pico Chip Bioanalyzer, respectively), suggesting that low initial input for particular samples may have led to higher background amplification (Figure S18C). We view this low input into sequencing libraries as an acceptable tradeoff for the high purity of our EV preparations. Unmapped reads were discarded from all further analyses. Principal-component analysis (PCA) of the remaining reads of each sample suggested distinct small RNA profiles (Figure S13).

In our second approach, quality control of the raw single-end RNA-seq reads was performed using the FastQC tool to filter out reads with

a quality score lower than 30 on the Phred scale.[86] Alignment and quantification of the reads onto the latest release of the reference human genome (GRCh38) was performed with the Shortstack alignment tool[85,87] mapping solely to miRbase. ShortStack offered the ability to provide counts for the same miRNA across multiple genomic loci. Both approaches provided nearly identical read percentages mapping to miRbase version 21 (Figure S19A). We then compared the normalization of miRNA counts relative to the total counts assigned to miRNAs via Shortstack (RPMM) to the default provided in the exceRpt pipeline, which normalizes miRNA counts to the total number of reads mapped to all small RNA libraries or the genome ($RPMM_{total}$ – the exceRpt default). These measures, therefore, quantify individual miRNAs relative to the total pool of miRNAs or the total pool of small RNAs present within EVs, respectively. We saw an overall strong positive correlation between RPMM and $RPMM_{total}$ values (rho 0.76–0.93, $p < 0.0001$, Spearman's rank) (Figure S19B). However, variability between replicates appeared higher for $RPMM_{total}$, especially among the highest expressed miRNAs (RPMM >100) (Figure S19C). Therefore, unless otherwhere specified, RPMM values were used for our analysis. miRNAs enriched in hESC-eEVs relative to other conditions were determined using ShortStack-derived counts processed by DESeq2 (version 1.34.0).[66] We also attenuated overestimates of logarithmic fold changes (LFCs) due to noise and high variability among miRNA counts by using the ashr package[88] within DESeq2. Comparisons to hESC-mEVs were handled separately from other comparisons due to larger variability between replicates seen via PCA (Figure S13) that may unduly influence normalization for differential expression analysis. We additionally validated our differential expression analyses using the alternative approach to normalize for RNA composition changes provided by the EdgeR tool[89] in the classic approach, again separating hESC-mEV comparisons out from others (Figure S20).

In silico analysis

An *in silico* analysis was performed to identify novel hESC-eEV-miRNAs. Of the 299 hESC-eEV-miRNAs identified by small RNA sequencing (Table S1) using miRPathDBv2.0,[90] we found that 179 hESC-eEV-miRNAs were predicted or experimentally validated to play a role in the VEGF and/or platelet-derived growth factor angiogenesis pathways (Table S7). Of those, we selected the ones emerging in all of the miRNA target prediction datasets provided on miRPathDBv2.0 (Diana-microT, miRDB, and TargetScan) ($p < 0.001$). Because hESC-eEVs induce angiogenesis at very low doses, to identify novel miRNAs that may contribute to this effect, we selected the ones that were highly expressed in hESC-eEVs (RPMM >3,000, also belonging in the list of top 50 hESC-eEV-miRNAs). The role of 3 of those miRNAs (miR-3074-5p, miR-4496, miR-4691-5p) was not reported in previous angiogenesis studies; thus, we marked these as novel hESC-eEV-miRNAs with a potential role in angiogenesis.

EV miRNA database search

The identified hESC-eEV-miRNAs were searched for in online databases, such as Vesiclepedia (http://microvesicles.org/) ExoCarta (http://exocarta.org/). These curated databases provide a compendium of miRNAs identified in several EVs/exosome preparations and from other sample types. Information from all of the above-mentioned exosome/EV miRNA databases was accessed in May 2021. Venn diagrams were created using the online tool at http://bioinformatics.psb.ugent.be/webtools/Venn/ to compare lists of miRNAs and identify the unique hESC-eEV miRNA biomarkers reported in this study.

miRNA secondary structure prediction

To predict the secondary structure of miRNAs, we used the online tool miRNAFold (https://tanuki.ibisc.univ-evry.fr/evryrna/mirnafold/mirnafold_form). The sequences of miRNAs of interest (hsa-miR-4496, hsa-miR-4691-5p) were obtained from miRBase (https://mirbase.org/). We selected the "RNAfold" option available on the miRNAFold website to generate the secondary structure prediction.

The input data consisted of the miRNA sequences in FASTA format, which were uploaded to the website. The prediction parameters were set to default values, with a temperature of 37°C and a minimum free energy (MFE) threshold of −20 kcal/mol. The output generated by the tool included the predicted secondary structures of the miRNAs, along with the corresponding MFE values. The secondary structures were visualized and analyzed using the RNAfold web server (http://rna.tbi.univie.ac.at/cgi-bin/RNAWebSuite/RNAfold.cgi).

miRNA conservation analysis

To investigate the conservation of hESC-eEV-miRNAs between humans and mice/rats, we used the MiRviewer tool.[91] A cutoff of 95% conservation score was implemented to identify miRNAs that are highly conserved across the species examined.

To assess the conservation of individual miRNAs of interest, namely miR-4496 and miR-4691-5p, we also used the Compara tool[92] from the Ensembl database.[93]

Statistical analysis

All of the biological replicates using human primary cells correspond to independent experiments from distinct expansions and passage numbers. All of the graphs (except proportion) are shown as data points, including mean ± SD on biological or technical replicates, as detailed in the figure legends. qRT-PCR data in graphs are shown as relative expressions as described by Livak and Schmittgen.[82] Statistical analysis was performed using GraphPad Prism 9.0.0 (GraphPad Software). Tests used to assess significance are detailed in each figure legend and precise p values of significant changes are indicated on the graph. A value of $p < 0.05$ was the level of nominal significance. For *in vitro* experiments, because each experimental dataset is an average of a large number of cultured cells, we assumed the data were normally distributed based on the central limit theorem. One-way ANOVA with Dunnett's multiple comparisons test was used to determine significant differences between the samples in our *in vitro* experiments (>2 groups).

DATA AND CODE AVAILABILITY

The authors confirm that the data supporting the findings of this study are available within the supplemental information. Raw data are available from the corresponding authors A.B. and A.H.B. upon request.

SUPPLEMENTAL INFORMATION

Supplemental information can be found online at https://doi.org/10.1016/j.ymthe.2023.11.023.

ACKNOWLEDGMENTS

D.K. is funded by a doctoral studentship from Medical Research Scotland (grant no. PhD-1293-2018). A.B. is supported by European Union Horizon 2020 CardioReGenix (grant no. 825670). A.H.B. is supported by the British Heart Foundation (grant no. CH/11/2/28733) and the Centre for Regenerative Medicine (grant no. CRMR/21/290009). M.B. is supported by the British Heart Foundation (grant no. FS/16/4/31831). J.R. is supported by the British Heart Foundation (grant no. RG/20/5/34796).

We acknowledge Stephen Mitchell at the School of Biological Sciences' electron microscopy unit for assistance with EM. We acknowledge the support of the Wellcome MultiUser Equipment Grant (WT104915MA). Flow cytometry data were generated with support from the QMRI Flow Cytometry and cell sorting facility, University of Edinburgh. NTA data were generated with support from the QMRI Nanosight facility, University of Edinburgh. Sequencing data were produced with assistance from BGI. This work has made use of the resources provided by the Edinburgh Compute and Data Facility (http://www.ecdf.ed.ac.uk/).

AUTHOR CONTRIBUTIONS

D.K. led the experimental design, execution, and statistical analysis, with feedback and input on the interpretation of the results provided by Andrea Caporali, M.B., A.B., and A.H.B. Bioinformatics analysis was performed by D.K. and M.B., with feedback and input on the interpretation of the results provided by J.R., M.B., A.B., and A.H.B. J.P.M. provided critical technical expertise and supervision on the miRNA mimic work. I.R.M., Alison Condie, and S.C. performed and supervised embryonic stem cell differentiations required for subsequent hESC-eEV isolation. D.K. wrote the manuscript. E.K. contributed valuable insights and feedback throughout the entire project. All of the authors reviewed and edited the manuscript. All of the authors listed have made a substantial, direct and intellectual contribution to the work, and approved it for publication.

DECLARATION OF INTERESTS

The authors declare the following financial interests/personal relationships which may be considered potential competing interests: D.K., J.C.M., M.B., A.B., and A.H.B. are named inventors on two patent application related to this work. The first patent was filed by the University Court of the University of Edinburgh (no. EP22386089.1) "Extracellular Vesicles That Promote Angiogenesis or Neovascularisation." The second patent was filed by Mirabilis Therapeutics BV (no. EP22386090.9) "ANGIOGENIC MIRNAS."

REFERENCES

1. (2017). WHO-Cardiovascular diseases (CVDs). http://www.who.int/en/news-room/fact-sheets/detail/cardiovascular-diseases-(cvds.
2. ESC Scientific Document Group, Alpert, J.S., Jaffe, A.S., Chaitman, B.R., Bax, J.J., Morrow, D.A., White, H.D., Thygesen, K., Alpert, J.S., Jaffe, A.S., et al. (2019). Fourth universal definition of myocardial infarction (2018). Eur. Heart J. *40*, 237–269.
3. Gnecchi, M., Zhang, Z., Ni, A., and Dzau, V.J. (2008). Paracrine mechanisms in adult stem cell signaling and therapy. Circ. Res. *103*, 1204–1219.
4. Mirotsou, M., Jayawardena, T.M., Schmeckpeper, J., Gnecchi, M., and Dzau, V.J. (2011). Paracrine mechanisms of stem cell reparative and regenerative actions in the heart. J. Mol. Cell. Cardiol. *50*, 280–289.
5. Hodgkinson, C.P., Gomez, J.A., Bareja, A., and Dzau, V.J. (2016). Role of paracrine mechanisms. Stem Cell Gene Therapy Cardiovascular Disease (Elsevier), pp. 39–48.
6. Théry, C., Witwer, K.W., Aikawa, E., Alcaraz, M.J., Anderson, J.D., Andriantsitohaina, R., Antoniou, A., Arab, T., Archer, F., Atkin-Smith, G.K., et al. (2018). Minimal information for studies of extracellular vesicles 2018 (MISEV2018): a position statement of the International Society for Extracellular Vesicles and update of the MISEV2014 guidelines. J. Extracell Vesicles *7*, 1535750.
7. Kesidou, D., da Costa Martins, P.A., de Windt, L.J., Brittan, M., Beqqali, A., and Baker, A.H. (2020). Extracellular Vesicle miRNAs in the Promotion of Cardiac Neovascularisation. Front. Physiol. *11*, 579892.
8. Yoshida, T., Asano, Y., and Ui-Tei, K. (2021). Modulation of MicroRNA Processing by Dicer via Its Associated dsRNA Binding Proteins. Noncoding. RNA *7*, 57.
9. Arroyo, J.D., Chevillet, J.R., Kroh, E.M., Ruf, I.K., Pritchard, C.C., Gibson, D.F., Mitchell, P.S., Bennett, C.F., Pogosova-Agadjanyan, E.L., Stirewalt, D.L., et al. (2011). Argonaute2 complexes carry a population of circulating microRNAs independent of vesicles in human plasma. Proc. Natl. Acad. Sci. USA *108*, 5003–5008.
10. Chevillet, J.R., Kang, Q., Ruf, I.K., Briggs, H.A., Vojtech, L.N., Hughes, S.M., Cheng, H.H., Arroyo, J.D., Meredith, E.K., Gallichotte, E.N., et al. (2014). Quantitative and stoichiometric analysis of the microRNA content of exosomes. Proc. Natl. Acad. Sci. USA *111*, 14888–14893.
11. Johnson, T.K., Zhao, L., Zhu, D., Wang, Y., Xiao, Y., Oguljahan, B., Zhao, X., Kirlin, W.G., Yin, L., Chilian, W.M., and Liu, D. (2019). Exosomes derived from induced vascular progenitor cells promote angiogenesis *in vitro* and in an *in vivo* rat hindlimb ischemia model. Am. J. Physiol. Heart Circ. Physiol. *317*, H765–H776.
12. Qiao, L., Hu, S., Liu, S., Zhang, H., Ma, H., Huang, K., Li, Z., Su, T., Vandergriff, A., Tang, J., et al. (2019). microRNA-21-5p dysregulation in exosomes derived from heart failure patients impairs regenerative potential. J. Clin. Invest. *129*, 2237–2250.
13. Cavallari, C., Figliolini, F., Tapparo, M., Cedrino, M., Trevisan, A., Positello, L., Rispoli, P., Solini, A., Migliaretti, G., Camussi, G., and Brizzi, M.F. (2020). miR-130a and Tgfβ Content in Extracellular Vesicles Derived from the Serum of Subjects at High Cardiovascular Risk Predicts their In-Vivo Angiogenic Potential. Sci. Rep. *10*, 706.
14. Valadi, H., Ekström, K., Bossios, A., Sjöstrand, M., Lee, J.J., and Lötvall, J.O. (2007). Exosome-mediated transfer of mRNAs and microRNAs is a novel mechanism of genetic exchange between cells. Nat. Cell Biol. *9*, 654–659.
15. Tosar, J.P., Gámbaro, F., Sanguinetti, J., Bonilla, B., Witwer, K.W., and Cayota, A. (2015). Assessment of small RNA sorting into different extracellular fractions revealed by high-throughput sequencing of breast cell lines. Nucleic Acids Res. *43*, 5601–5616.
16. Pegtel, D.M., Cosmopoulos, K., Thorley-Lawson, D.A., van Eijndhoven, M.A.J., Hopmans, E.S., Lindenberg, J.L., de Gruijl, T.D., Würdinger, T., and Middeldorp, J.M. (2010). Functional delivery of viral miRNAs via exosomes. Proc. Natl. Acad. Sci. USA *107*, 6328–6333.
17. Montecalvo, A., Larregina, A.T., Shufesky, W.J., Stolz, D.B., Sullivan, M.L.G., Karlsson, J.M., Baty, C.J., Gibson, G.A., Erdos, G., Wang, Z., et al. (2012). Mechanism of transfer of functional microRNAs between mouse dendritic cells via exosomes. Blood *119*, 756–766.

18. Squadrito, M.L., Baer, C., Burdet, F., Maderna, C., Gilfillan, G.D., Lyle, R., Ibberson, M., and de Palma, M. (2014). Endogenous RNAs Modulate MicroRNA Sorting to Exosomes and Transfer to Acceptor Cells. Cell Rep. *8*, 1432–1446.

19. Jeppesen, D.K., Fenix, A.M., Franklin, J.L., Higginbotham, J.N., Zhang, Q., Zimmerman, L.J., Liebler, D.C., Ping, J., Liu, Q., Evans, R., et al. (2019). Reassessment of Exosome Composition In Brief Article Reassessment of Exosome Composition. Cell *177*, 428–445.e18.

20. Bolukbasi, M.F., Mizrak, A., Ozdener, G.B., Madlener, S., Ströbel, T., Erkan, E.P., Fan, J.B., Breakefield, X.O., and Saydam, O. (2012). miR-1289 and 'Zipcode'-like Sequence Enrich mRNAs in Microvesicles. Mol. Ther. Nucleic Acids *1*, e10.

21. Villarroya-Beltri, C., Gutiérrez-Vázquez, C., Sánchez-Cabo, F., Pérez-Hernández, D., Vázquez, J., Martin-Cofreces, N., Martinez-Herrera, D.J., Pascual-Montano, A., Mittelbrunn, M., and Sánchez-Madrid, F. (2013). Sumoylated hnRNPA2B1 controls the sorting of miRNAs into exosomes through binding to specific motifs. Nat. Commun. *4*, 2980.

22. Koppers-Lalic, D., Hackenberg, M., Bijnsdorp, I.V., van Eijndhoven, M.A.J., Sadek, P., Sie, D., Zini, N., Middeldorp, J.M., Ylstra, B., de Menezes, R.X., et al. (2014). Nontemplated nucleotide additions distinguish the small RNA composition in cells from exosomes. Cell Rep. *8*, 1649–1658.

23. Santangelo, L., Giurato, G., Cicchini, C., Montaldo, C., Mancone, C., Tarallo, R., Battistelli, C., Alonzi, T., Weisz, A., and Tripodi, M. (2016). The RNA-Binding Protein SYNCRIP Is a Component of the Hepatocyte Exosomal Machinery Controlling MicroRNA Sorting. Cell Rep. *17*, 799–808.

24. Temoche-Diaz, M.M., Shurtleff, M.J., Nottingham, R.M., Yao, J., Fadadu, R.P., Lambowitz, A.M., and Schekman, R. (2019). Distinct mechanisms of microRNA sorting into cancer cell-derived extracellular vesicle subtypes. Elife *8*, e47544.

25. Garcia-Martin, R., Wang, G., Brandão, B.B., Zanotto, T.M., Shah, S., Kumar Patel, S., Schilling, B., and Kahn, C.R. (2021). MicroRNA sequence codes for small extracellular vesicle release and cellular retention. Nature *601*, 446–451.

26. Ranghino, A., Cantaluppi, V., Grange, C., Vitillo, L., Fop, F., Biancone, L., Deregibus, M.C., Tetta, C., Segoloni, G.P., and Camussi, G. (2012). Endothelial Progenitor Cell-Derived Microvesicles Improve Neovascularization in a Murine Model of Hindlimb Ischemia. Int. J. Immunopathol. Pharmacol. *25*, 75–85.

27. Barile, L., Lionetti, V., Cervio, E., Matteucci, M., Gherghiceanu, M., Popescu, L.M., Torre, T., Siclari, F., Moccetti, T., and Vassalli, G. (2014). Extracellular vesicles from human cardiac progenitor cells inhibit cardiomyocyte apoptosis and improve cardiac function after myocardial infarction. Cardiovasc. Res. *103*, 530–541.

28. Beltrami, C., Besnier, M., Shantikumar, S., Shearn, A.I.U., Rajakaruna, C., Laftah, A., Sessa, F., Spinetti, G., Petretto, E., Angelini, G.D., and Emanueli, C. (2017). Human Pericardial Fluid Contains Exosomes Enriched with Cardiovascular-Expressed MicroRNAs and Promotes Therapeutic Angiogenesis. Mol. Ther. *25*, 679–693.

29. Mathiyalagan, P., Liang, Y., Kim, D., Misener, S., Thorne, T., Kamide, C.E., Klyachko, E., Losordo, D.W., Hajjar, R.J., and Sahoo, S. (2017). Angiogenic Mechanisms of Human CD34+ Stem Cell Exosomes in the Repair of Ischemic Hindlimb. Circ. Res. *120*, 1466–1476.

30. Ribeiro-Rodrigues, T.M., Laundos, T.L., Pereira-Carvalho, R., Batista-Almeida, D., Pereira, R., Coelho-Santos, V., Silva, A.P., Fernandes, R., Zuzarte, M., Enguita, F.J., et al. (2017). Exosomes secreted by cardiomyocytes subjected to ischaemia promote cardiac angiogenesis. Cardiovasc. Res. *113*, 1338–1350.

31. Zhu, J., Lu, K., Zhang, N., Zhao, Y., Ma, Q., Shen, J., Lin, Y., Xiang, P., Tang, Y., Hu, X., et al. (2018). Myocardial reparative functions of exosomes from mesenchymal stem cells are enhanced by hypoxia treatment of the cells via transferring microRNA-210 in an nSMase2-dependent way. Artif. Cells Nanomed. Biotechnol. *46*, 1659–1670.

32. Li, H., Liao, Y., Gao, L., Zhuang, T., Huang, Z., Zhu, H., and Ge, J. (2018). Coronary Serum Exosomes Derived from Patients with Myocardial Ischemia Regulate Angiogenesis through the miR-939-mediated Nitric Oxide Signaling Pathway. Theranostics *8*, 2079–2093.

33. Adamiak, M., Cheng, G., Bobis-Wozowicz, S., Zhao, L., Kedracka-Krok, S., Samanta, A., Karnas, E., Xuan, Y.-T., Skupien-Rabian, B., Chen, X., et al. (2018). Induced Pluripotent Stem Cell (iPSC)-Derived Extracellular Vesicles Are Safer and More Effective for Cardiac Repair than iPSCs. Circ. Res. *122*, 296–309.

34. Koga, H., Sugiyama, S., Kugiyama, K., Watanabe, K., Fukushima, H., Tanaka, T., Sakamoto, T., Yoshimura, M., Jinnouchi, H., and Ogawa, H. (2005). Elevated Levels of VE-Cadherin-Positive Endothelial Microparticles in Patients With Type 2 Diabetes Mellitus and Coronary Artery Disease. J. Am. Coll. Cardiol. *45*, 1622–1630.

35. Werner, N., Wassmann, S., Ahlers, P., Kosiol, S., and Nickenig, G. (2006). Circulating CD31 $^{+}$/Annexin V $^{+}$ Apoptotic Microparticles Correlate With Coronary Endothelial Function in Patients With Coronary Artery Disease. Arterioscler. Thromb. Vasc. Biol. *26*, 112–116.

36. Nozaki, T., Sugiyama, S., Koga, H., Sugamura, K., Ohba, K., Matsuzawa, Y., Sumida, H., Matsui, K., Jinnouchi, H., and Ogawa, H. (2009). Significance of a Multiple Biomarkers Strategy Including Endothelial Dysfunction to Improve Risk Stratification for Cardiovascular Events in Patients at High Risk for Coronary Heart Disease. J. Am. Coll. Cardiol. *54*, 601–608.

37. Leroyer, A.S., Rautou, P.E., Silvestre, J.S., Castier, Y., Lesèche, G., Devue, C., Duriez, M., Brandes, R.P., Lutgens, E., Tedgui, A., and Boulanger, C.M. (2008). CD40 ligand+ microparticles from human atherosclerotic plaques stimulate endothelial proliferation and angiogenesis a potential mechanism for intraplaque neovascularization. J. Am. Coll. Cardiol. *52*, 1302–1311.

38. Ridger, V.C., Boulanger, C.M., Angelillo-Scherrer, A., Badimon, L., Blanc-Brude, O., Bochaton-Piallat, M.L., Boilard, E., Buzas, E.I., Caporali, A., Dignat-George, F., et al. (2017). Microvesicles in vascular homeostasis and diseases position paper of the european society of cardiology (ESC) working group on atherosclerosis and vascular biology. Thromb. Haemost. *117*, 1296–1316.

39. Lacroix, R., Sabatier, F., Mialhe, A., Basire, A., Pannell, R., Borghi, H., Robert, S., Lamy, E., Plawinski, L., Camoin-Jau, L., et al. (2007). Activation of plasminogen into plasmin at the surface of endothelial microparticles: A mechanism that modulates angiogenic properties of endothelial progenitor cells *in vitro*. Blood *110*, 2432–2439.

40. Ma, J., Tao, X., and Huang, Y. (2020). Silencing microRNA-210 in Hypoxia-Induced HUVEC-Derived Extracellular Vesicles Inhibits Hemangioma. Cerebrovasc. Dis. *49*, 462–473.

41. Liang, H.Z., Li, S.F., Zhang, F., Wu, M.Y., Li, C.L., Song, J.X., Lee, C., and Chen, H. (2018). Effect of Endothelial Microparticles Induced by Hypoxia on Migration and Angiogenesis of Human Umbilical Vein Endothelial Cells by Delivering MicroRNA-19b. Chin. Med. J. *131*, 2726–2733.

42. van Balkom, B.W.M., de Jong, O.G., Smits, M., Brummelman, J., den Ouden, K., de Bree, P.M., van Eijndhoven, M.A.J., Pegtel, D.M., Stoorvogel, W., Würdinger, T., and Verhaar, M.C. (2013). Endothelial cells require miR-214 to secrete exosomes that suppress senescence and induce angiogenesis in human and mouse endothelial cells. Blood *121*, 3997–4006. S1-S15.

43. Chen, C.W., Wang, L.L., Zaman, S., Gordon, J., Arisi, M.F., Venkataraman, C.M., Chung, J.J., Hung, G., Gaffey, A.C., Spruce, L.A., et al. (2018). Sustained release of endothelial progenitor cell-derived extracellular vesicles from shear-thinning hydrogels improves angiogenesis and promotes function after myocardial infarction. Cardiovasc. Res. *114*, 1029–1040.

44. Ou, Z.-J., Chang, F.-J., Luo, D., Liao, X.-L., Wang, Z.-P., Zhang, X., Xu, Y.-Q., and Ou, J.-S. (2011). Endothelium-derived microparticles inhibit angiogenesis in the heart and enhance the inhibitory effects of hypercholesterolemia on angiogenesis. Am. J. Physiol. Endocrinol. Metab. *300*, E661–E668.

45. Kim, E.J., Li, R.K., Weisel, R.D., Mickle, D.A., Jia, Z.Q., Tomita, S., Sakai, T., and Yau, T.M. (2001). Angiogenesis by endothelial cell transplantation. J. Thorac. Cardiovasc. Surg. *122*, 963–971.

46. Chekanov, V., Akhtar, M., Tchekanov, G., Dangas, G., Shehzad, M.Z., Tio, F., Adamian, M., Colombo, A., Roubin, G., Leon, M.B., et al. (2003). Transplantation of autologous endothelial cells induces angiogenesis. Pacing Clin. Electrophysiol. *26*, 496–499.

47. MacAskill, M.G., Saif, J., Condie, A., Jansen, M.A., MacGillivray, T.J., Tavares, A.A.S., Fleisinger, L., Spencer, H.L., Besnier, M., Martin, E., et al. (2018). Robust Revascularization in Models of Limb Ischemia Using a Clinically Translatable Human Stem Cell-Derived Endothelial Cell Product. Mol. Ther. *26*, 1669–1684.

48. McCracken, I.R., Taylor, R.S., Kok, F.O., de la Cuesta, F., Dobie, R., Henderson, B.E.P., Mountford, J.C., Caudrillier, A., Henderson, N.C., Ponting, C.P., and Baker, A.H. (2020). Transcriptional dynamics of pluripotent stem cell-derived endothelial cell differentiation revealed by single-cell RNA sequencing. Eur. Heart J. *41*, 1024–1036.

49. Spiroski, A.M., McCracken, I.R., Thomson, A., Magalhaes-Pinto, M., Lalwani, M.K., Newton, K.J., Miller, E., Bénézech, C., Hadoke, P., Brittan, M., et al. (2022). Human embryonic stem cell-derived endothelial cell product injection attenuates cardiac remodeling in myocardial infarction. Front. Cardiovasc. Med. *9*, 953211.

50. Lai, W.H., Ho, J.C.Y., Chan, Y.C., Ng, J.H.L., Au, K.W., Wong, L.Y., Siu, C.W., and Tse, H.F. (2013). Attenuation of Hind-Limb Ischemia in Mice with Endothelial-Like Cells Derived from Different Sources of Human Stem Cells. PLoS One *8*, e57876.

51. Tompkins, B.A., Balkan, W., Winkler, J., Gyöngyösi, M., Goliasch, G., Fernández-Avilés, F., and Hare, J.M. (2018). IMPACT: Preclinical studies of cell therapy for human disease. Circ. Res. *122*, 1006–1020.

52. Johnston, P.V., Sasano, T., Mills, K., Evers, R., Lee, S.T., Smith, R.R., Lardo, A.C., Lai, S., Steenbergen, C., Gerstenblith, G., et al. (2009). Engraftment, Differentiation and Functional Benefits of Autologous Cardiosphere-Derived Cells in Porcine Ischemic Cardiomyopathy. Circulation *120*, 1075–1083.

53. Bister, N., Pistono, C., Huremagic, B., Jolkkonen, J., Giugno, R., and Malm, T. (2020). Hypoxia and extracellular vesicles: A review on methods, vesicular cargo and functions. J. Extracell. Vesicles *10*, e12002.

54. O'Brien, K., Breyne, K., Ughetto, S., Laurent, L.C., and Breakefield, X.O. (2020). RNA delivery by extracellular vesicles in mammalian cells and its applications. Nat. Rev. Mol. Cell Biol. *21*, 585–606.

55. de Rie, D., Abugessaisa, I., Alam, T., Arner, E., Arner, P., Ashoor, H., Åström, G., Babina, M., Bertin, N., Burroughs, A.M., et al. (2017). An integrated expression atlas of miRNAs and their promoters in human and mouse. Nat. Biotechnol. *35*, 872–878.

56. Qu, M., Pan, J., Wang, L., Zhou, P., Song, Y., Wang, S., Jiang, L., Geng, J., Zhang, Z., Wang, Y., et al. (2019). MicroRNA-126 Regulates Angiogenesis and Neurogenesis in a Mouse Model of Focal Cerebral Ischemia. Mol. Ther. Nucleic Acids *16*, 15–25.

57. Wang, S., Aurora, A.B., Johnson, B.A., Qi, X., McAnally, J., Hill, J.A., Richardson, J.A., Bassel-Duby, R., and Olson, E.N. (2008). The Endothelial-Specific MicroRNA miR-126 Governs Vascular Integrity and Angiogenesis. Dev. Cell *15*, 261–271.

58. Fish, J.E., Santoro, M.M., Morton, S.U., Yu, S., Yeh, R.F., Wythe, J.D., Ivey, K.N., Bruneau, B.G., Stainier, D.Y.R., and Srivastava, D. (2008). miR-126 Regulates Angiogenic Signaling and Vascular Integrity. Dev. Cell *15*, 272–284.

59. Jakob, P., Doerries, C., Briand, S., Mocharla, P., Kränkel, N., Besler, C., Mueller, M., Manes, C., Templin, C., Baltes, C., et al. (2012). Loss of angiomir-126 and 130a in angiogenic early outgrowth cells from patients with chronic heart failure: Role for impaired *in vivo* neovascularization and cardiac repair capacity. Circulation *126*, 2962–2975.

60. Liu, Y., Li, Q., Hosen, M.R., Zietzer, A., Flender, A., Levermann, P., Schmitz, T., Frühwald, D., Goody, P., Nickenig, G., et al. (2019). Atherosclerotic conditions promote the packaging of functional MicroRNA-92a-3p into endothelial microvesicles. Circ. Res. *124*, 575–587.

61. Li, Y.Z., Wen, L., Wei, X., Wang, Q.R., Xu, L.W., Zhang, H.M., and Liu, W.C. (2016). Inhibition of miR-7 promotes angiogenesis in human umbilical vein endothelial cells by upregulating VEGF via KLF4. Oncol. Rep. *36*, 1569–1575.

62. Wang, Q.-Z., Zhao, Z.-L., Liu, C., and Zheng, J.-W. (2021). Exosome-derived miR-196b-5p facilitates intercellular interaction in infantile hemangioma via down-regulating CDKN1B. Ann. Transl. Med. *9*, 394.

63. Guo, B., Zhao, Z., Wang, Z., Li, Q., Wang, X., Wang, W., Song, T., and Huang, C. (2017). MicroRNA-302b-3p Suppresses Cell Proliferation Through AKT Pathway by Targeting IGF-1R in Human Gastric Cancer. Cell. Physiol. Biochem. *42*, 1701–1711.

64. Cao, J., Li, L., Han, X., Cheng, H., Chen, W., Qi, K., Chen, C., Wu, Q., Niu, M., Zeng, L., and Xu, K. (2019). miR-302 cluster inhibits angiogenesis and growth of K562 leukemia cells by targeting VEGFA. Onco. Targets Ther. *12*, 433–441.

65. Jansen, F., Yang, X., Hoelscher, M., Cattelan, A., Schmitz, T., Proebsting, S., Wenzel, D., Vosen, S., Franklin, B.S., Fleischmann, B.K., et al. (2013). Endothelial microparticle-mediated transfer of microRNA-126 promotes vascular endothelial cell repair via spred1 and is abrogated in glucose-damaged endothelial microparticles. Circulation *128*, 2026–2038.

66. Love, M.I., Huber, W., and Anders, S. (2014). Moderated estimation of fold change and dispersion for RNA-seq data with DESeq2. Genome Biol. *15*, 550.

67. Kuschnerus, K., Straessler, E.T., Müller, M.F., Lüscher, T.F., Landmesser, U., and Kränkel, N. (2019). Increased Expression of miR-483-3p Impairs the Vascular Response to Injury in Type 2 Diabetes. Diabetes *68*, 349–360.

68. Gu, K.L., Zhang, Q., Yan, Y., Li, T.T., Duan, F.F., Hao, J., Wang, X.W., Shi, M., Wu, D.R., Guo, W.T., and Wang, Y. (2016). Pluripotency-associated miR-290/302 family of microRNAs promote the dismantling of naive pluripotency. Cell Res. *26*, 350–366.

69. Cao, Y.P., Pan, M., Song, Y.L., Zhang, H.L., Sui, H.T., Shan, B.C., and Piao, H.X. (2019). MiR-302 a/b/c suppresses tumor angiogenesis in hepatocellular carcinoma by targeting MACC1. Eur. Rev. Med. Pharmacol. Sci. *23*, 7863–7873.

70. Montesano, R., Carrozzino, F., and Soulié, P. (2007). Low concentrations of transforming growth factor-beta-1 induce tubulogenesis in cultured mammary epithelial cells. BMC Dev. Biol. *7*, 7.

71. Park, S.G., Kang, Y.S., Ahn, Y.H., Lee, S.H., Kim, K.R., Kim, K.W., Koh, G.Y., Ko, Y.G., and Kim, S. (2002). Dose-dependent biphasic activity of tRNA synthetase-associating factor, p43, in angiogenesis. J. Biol. Chem. *277*, 45243–45248.

72. Mourad, O., Yee, R., Li, M., and Nunes, S.S. (2023). Modeling Heart Diseases on a Chip: Advantages and Future Opportunities. Circ. Res. *132*, 483–497.

73. Witwer, K.W., and Wolfram, J. (2021). Extracellular vesicles versus synthetic nanoparticles for drug delivery. Nat. Rev. Mater. *6*, 103–106.

74. Moghiman, T., Barghchi, B., Esmaeili, S.A., Shabestari, M.M., Tabaee, S.S., and Momtazi-Borojeni, A.A. (2021). Therapeutic angiogenesis with exosomal microRNAs: an effectual approach for the treatment of myocardial ischemia. Heart Fail. Rev. *26*, 205–213.

75. Li, G., Chen, T., Dahlman, J., Eniola-Adefeso, L., Ghiran, I.C., Kurre, P., Lam, W.A., Lang, J.K., Marbán, E., Martín, P., et al. (2023). Current challenges and future directions for engineering extracellular vesicles for heart, lung, blood and sleep diseases. J. Extracell. Vesicles *12*, 12305.

76. Reshke, R., Taylor, J.A., Savard, A., Guo, H., Rhym, L.H., Kowalski, P.S., Trung, M.T., Campbell, C., Little, W., Anderson, D.G., and Gibbings, D. (2020). Reduction of the therapeutic dose of silencing RNA by packaging it in extracellular vesicles via a pre-microRNA backbone. Nat. Biomed. Eng. *4*, 52–68.

77. Nawaz, M., Heydarkhan-Hagvall, S., Tangruksa, B., González-King Garibotti, H., Jing, Y., Maugeri, M., Kohl, F., Hultin, L., Reyahi, A., Camponeschi, A., et al. (2023). Lipid Nanoparticles Deliver the Therapeutic VEGFA mRNA In Vitro and In Vivo and Transform Extracellular Vesicles for Their Functional Extensions. Adv. Sci. *10*, e2206187.

78. Thomson, J.A., Itskovitz-Eldor, J., Shapiro, S.S., Waknitz, M.A., Swiergiel, J.J., Marshall, V.S., and Jones, J.M. (1998). Embryonic stem cell lines derived from human blastocysts. Science *282*, 1145–1147.

79. De Sousa, P.A., Tye, B.J., Bruce, K., Dand, P., Russell, G., Collins, D.M., Greenshields, A., McDonald, K., Bradburn, H., Laurie, A., et al. (2016). Derivation of the clinical grade human embryonic stem cell line RCe015-A (RC-11). Stem Cell Res. *17*, 42–48.

80. Kotrbová, A., Štěpka, K., Maška, M., Pálenik, J.J., Ilkovics, L., Klemová, D., Kravec, M., Hubatka, F., Dave, Z., Hampl, A., et al. (2019). TEM ExosomeAnalyzer: a computer-assisted software tool for quantitative evaluation of extracellular vesicles in transmission electron microscopy images. J. Extracell. Vesicles *8*, 1560808.

81. Abramoff, M.D., Magalhaes, P.J., and Ram, S.J. (2004). Image Processing with ImageJ. Biophotonics Int. *11*, 36–42.

82. Livak, K.J., and Schmittgen, T.D. (2001). Analysis of relative gene expression data using real-time quantitative PCR and the 2(-Delta Delta C(T)) Method. Methods *25*, 402–408.

83. Carpentier, G., Berndt, S., Ferratge, S., Rasband, W., Cuendet, M., Uzan, G., and Albanese, P. (2020). Angiogenesis Analyzer for ImageJ — A comparative morphometric analysis of "Endothelial Tube Formation Assay" and "Fibrin Bead Assay". Sci. Rep. *10*, 11568.

84. Suarez-Arnedo, A., Torres Figueroa, F., Clavijo, C., Arbeláez, P., Cruz, J.C., and Muñoz-Camargo, C. (2020). An image J plugin for the high throughput image analysis of *in vitro* scratch wound healing assays. PLoS One *15*, e0232565.

85. Rozowsky, J., Kitchen, R.R., Park, J.J., Subramanian, S.L., and Milosavljevic, A. (2019). exceRpt: A Comprehensive Analytic Platform for Extracellular RNA Profiling. Cell Syst *8*, 352–357.e3.

86. Andrews, S. (2010). FASTQC. A Quality Control Tool for High Throughput Sequence Data (BibSonomy).

87. Axtell, M.J. (2013). ShortStack: Comprehensive annotation and quantification of small RNA genes. RNA *19*, 740–751.

88. Stephens, M. (2017). False discovery rates: a new deal. Biostatistics *18*, 275–294.

89. Robinson, M.D., McCarthy, D.J., and Smyth, G.K. (2010). edgeR: a Bioconductor package for differential expression analysis of digital gene expression data. Bioinformatics *26*, 139–140.

90. Kehl, T., Kern, F., Backes, C., Fehlmann, T., Stöckel, D., Meese, E., Lenhof, H.P., and Keller, A. (2020). miRPathDB 2.0: a novel release of the miRNA Pathway Dictionary Database. Nucleic Acids Res. *48*, D142–D147.

91. Kiezun, A., Artzi, S., Modai, S., Volk, N., Isakov, O., and Shomron, N. (2012). MiRviewer: A multispecies microRNA homologous viewer. BMC Res. Notes *5*, 92.

92. Herrero, J., Muffato, M., Beal, K., Fitzgerald, S., Gordon, L., Pignatelli, M., Vilella, A.J., Searle, S.M.J., Amode, R., Brent, S., et al. (2016). Ensembl comparative genomics resources. Database *96*.

93. Birney, E., Andrews, T.D., Bevan, P., Caccamo, M., Chen, Y., Clarke, L., Coates, G., Cuff, J., Curwen, V., Cutts, T., et al. (2004). An Overview of Ensembl. Genome Res. *14*, 925–928.

YMTHE, Volume *32*

Supplemental Information

Extracellular vesicles from differentiated stem cells contain novel proangiogenic miRNAs and induce angiogenic responses at low doses

Despoina Kesidou, Matthew Bennett, João P. Monteiro, Ian R. McCracken, Eftychia Klimi, Julie Rodor, Alison Condie, Scott Cowan, Andrea Caporali, Jan B.M. Wit, Joanne C. Mountford, Mairi Brittan, Abdelaziz Beqqali, and Andrew H. Baker

Table S1. **Small RNA sequencing data.** Supplemented as Excel file

Table S2. **Antibodies used for staining human embryonic stem cell-derived endothelial cell products (hESC-ECPs) for flow cytometric characterisation.** Cells stained for endothelial (CD31 and CD144) (right panels), pluripotent markers (TRA-181 and SSEA-4) (left panels), and their corresponding isotype controls**.**

	Antibody Name
Pluripotent	PE Mouse anti-human TRA-1-81 Antigen, BD Biosciences, 560161
	PE Mouse IgM, κ Isotype Control, BD Biosciences, 555584
	Alexa Fluor 647 Mouse anti-SSEA-4, BD Biosciences, 560796
	APC, anti-human, SSEA-4 Antibody, REAfinity, Miltenyi, 130-123-815
	Alexa Fluor 647 Mouse IgG3, κ Isotype Control, BD Biosciences, 560803
	PE, anti-human, TRA-1-60 Antibody, REAfinity, Miltenyi, 130-122-921
	PE, human IgG1, REA Control Antibody (S), REAfinity, Miltenyi, 130-113-438
Endothelial	PE Mouse anti-Human CD144, BD Biosciences, 580410
	PE Mouse IgG1, κ Isotype Control, BD Biosciences, 555749
	APC Anti-Human CD31, eBioscience, 17-0319-42
	APC Mouse IgG1, κ Isotype Control, eBioscience, 17-4714-42

Table S3. Specifications of samples analysed by small RNA sequencing.

Sample Name	RNA Concentration	RNA Sample Volume (ul)	Total RNA Quantity	Number of Flasks used for media collection	Total volume of media collected (ml)	Number of EVs isolated from the conditioned media	Number of Cells in the flasks used for media collection
hESC-ECP #1	278 ng/ul	30	8.3 µg	n/a	n/a	n/a	n/a
hESC-ECP #2	533 ng/ul	30	16 µg	n/a	n/a	n/a	n/a
hESC-ECP #3	154 ng/ul	30	4.6 µg	n/a	n/a	n/a	n/a
hESC-eEV #1	91 pg/ul	30	2.7 ng	3*T75 + 2*T25	80	4*10^10	8.8*10^6
hESC-eEV #2	35 pg/ul	30	1.1 ng	4*T25	36	1.8*10^10	3.2*10^6
hESC-eEV #3	116 pg/ul	30	3.9 ng	2*T75	40	4.6*10^10	4.8*10^6
hESC-mEV #1	234 pg/ul	30	7 ng	2*T25	16	1.2*10^11	n/a
hESC-mEV #2	743 pg/ul	30	22.3 ng	1*T75+ 1*T25	58	6.8*10^11	n/a
hESC-mEV #3	826 pg/ul	30	24.8 ng	1*T75+ 1*T25	58	9.8*10^11	n/a
HUVEC Hypoxic EV #1	39 pg/ul	30	1.2 ng	1*T150	15	1.2*10^10	6*10^6
HUVEC Hypoxic EV #2	103 pg/ul	30	3.1 ng	1*T150	15	1.9*10^10	6.3*10^6
HUVEC Hypoxic EV #3	75 pg/ul	30	2.3ng	4*T150	60	10^11	3.4*10^7
RC11-eEV #1	170 pg/ul	30	5.1ng	3*T75	66	1.5*10^11	n/a
RC11-eEV #2	222 pg/ul	30	6.7ng	3*T75	66	6.5*10^11	n/a
RC11-eEV #3	211.5 pg/ul	30	6.4ng	3*T75	66	1.8*10^11	n/a

Table S4. **List of primary and secondary antibodies used for Western Blot.**

Primary Antibodies	Product ID/ Company	Host	Reactivity	Dilution
Calnexin	ab22595, Abcam	Rabbit	Human, Mouse, Rat	1:1000
CD63	sc-5275, Santa-Cruz Biotechnology	Mouse	Human, Mouse, Rat	1:500
CD81	sc-7637, Santa-Cruz Biotechnology	Mouse	Human, Mouse, Rat	1:500
CD9	sc-59140 , Santa-Cruz Biotechnology	Mouse	Human, Mouse, Rat	1:500
CD31	Ab76533, Abcam	Rabbit	Human, Mouse, Rat	1:5000
**Secondary Antibodies**	**Product ID/ Company**	**Host**	**Reactivity**	**Dilution**
IRDye® 800CW Goat anti-Mouse IgG(H+L)	926-32210, LICOR	Goat	Mouse	1:15,000
IRDye® 680RD Goat anti-Rabbit IgG (H+L) secondary antibody	926-68072, LICOR	Goat	Rabbit	1:15,000

Table S5. List of miRNA mimics used for miRNA overexpression.

Name	Target	Assay ID
miRIDIAN microRNA Mimic Negative Control #1	Negative Control (miRC)	CN-001000-01, Dharmacon
miRIDIAN microRNA Human hsa-miR-4496 - Mimic	miR-4496	C-302100-00, Dharmacon
miRIDIAN microRNA hsa-miR-4691-5p- Mimic	miR-4691-5p	C-302248-00, Dharmacon
miRIDIAN microRNA hsa-miR-126-5p - Mimic	miR-126-5p	C-300625-05, Dharmacon

Table S6. List of miRNA TaqMan probes used for qRT-PCR analysis.

miRNA ID	Assay ID
RNU48	001006, ThermoFisher Scientific
miR-4496	465234_mat, ThermoFisher Scientific
miR-4691-5p	463008_mat, ThermoFisher Scientific
miR-126-5p	002228, ThermoFisher Scientific

Table S7. ***In silico* analysis identifying novel hESC-eEV-miRNAs with a potential role in angiogenesis**. Supplemented as Excel file

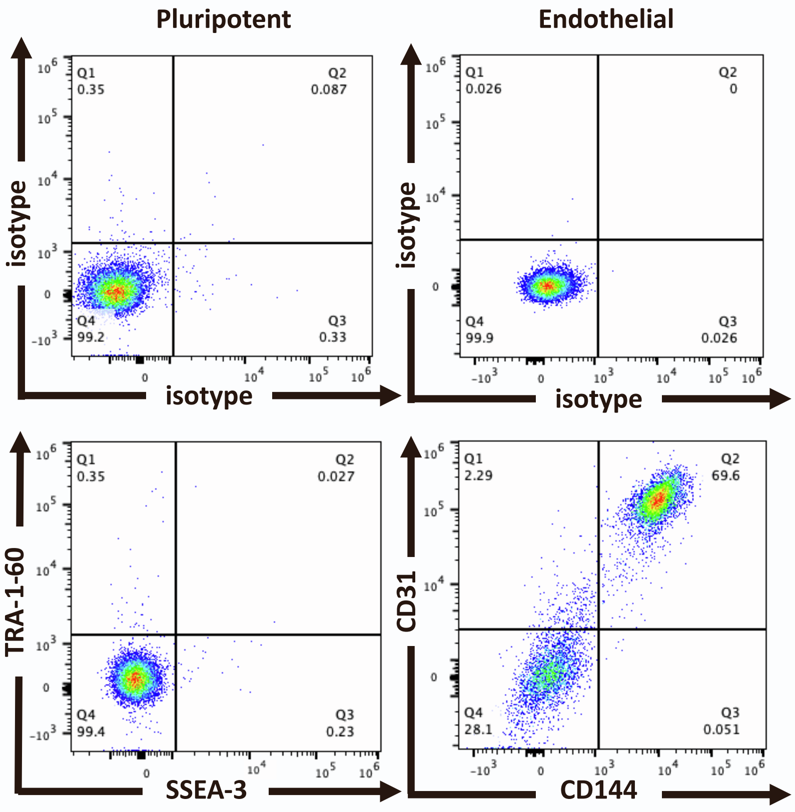

Figure S1. Representative flow cytometric analysis of human embryonic stem cell-derived endothelial cell products (hESC-ECPs). Cells were stained for endothelial (CD31 and CD144) (right panels), pluripotent markers (TRA-1-60 and SSEA-3) (left panels), and their corresponding isotype controls (top panels).

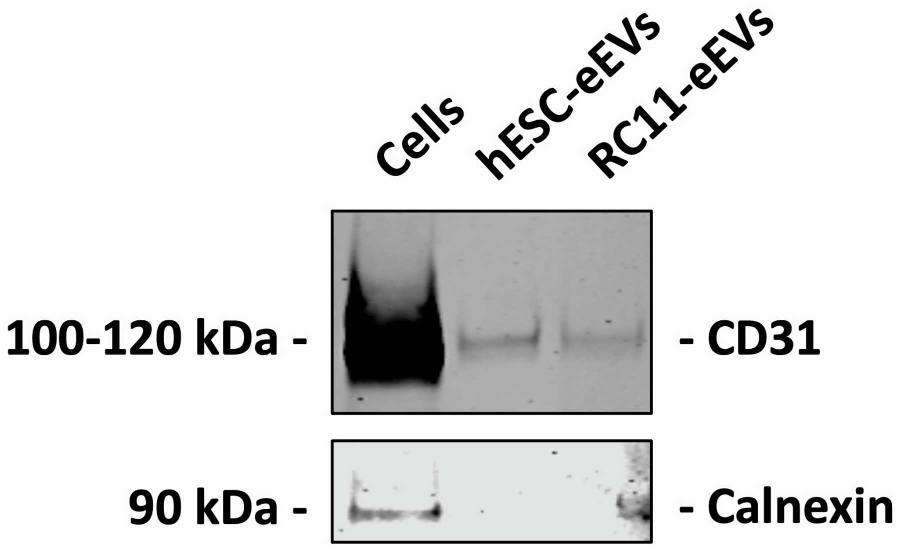

Figure S2. Western blot of Endothelial cell marker CD31 in hESC-eEVs from two independent cell lines H9 (hESC-eEVs) and RC11 (RC11-eEVs). Whole cell lysates of day 8 Endothelial cell product were used as a positive control. Calnexin was used as cellular protein control.

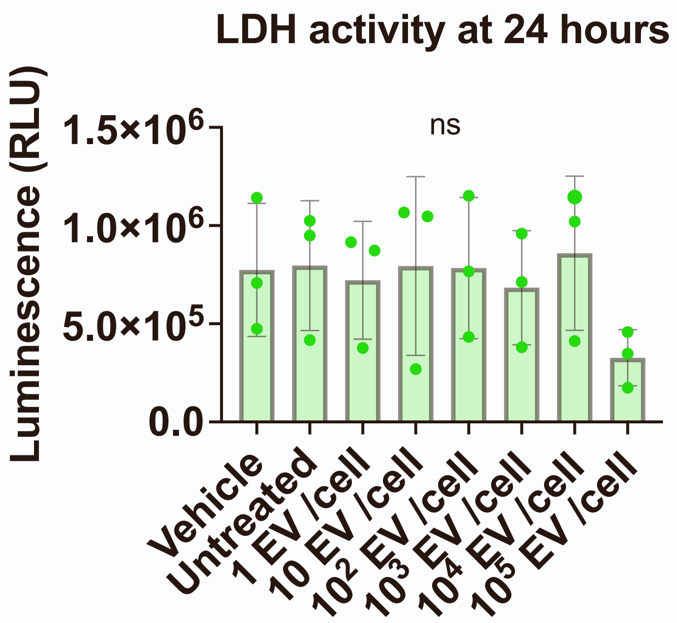

Figure S3. Lactate dehydrogenase activity (LDH) following treatment with extracellular vesicles from differentiated embryonic stem cells (hESC-eEVs). Plots represent LDH activity, quantified by luminescence in relative luminescence units (RLU) on the y-axis in response to treatment with increasing hESC-eEV doses (x-axis). LDH activity was measured at 24 hours (n=3). Statistical comparison was performed using one-way ANOVA with Dunnett's multiple comparisons test.

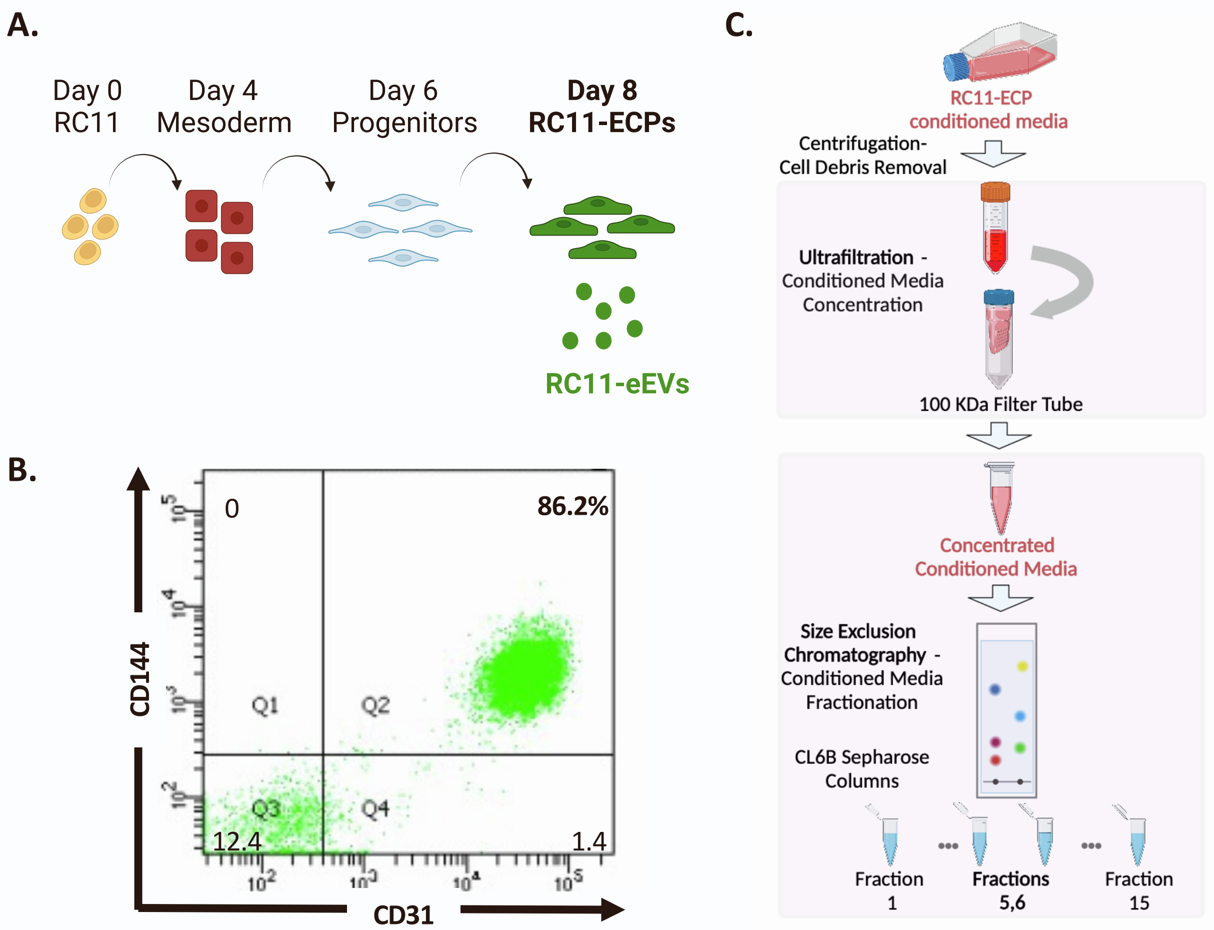

Figure S4. Isolation of extracellular vesicles (EVs) from RC11 human embryonic stem cell-derived endothelial cell product (RC11-ECP) conditioned media. A. Schematic representation of RC11-ECP differentiation and EV secretion from cells at the endothelial-enriched (day 8) stage. **B**. Representative flow cytometric analysis of RC11-ECP. Cells were stained for the endothelial markers CD31 and CD144. **C.** Workflow of EV isolation from RC11-ECP conditioned media by a combination of ultrafiltration with size exclusion chromatography (SEC). Created with BioRender.com

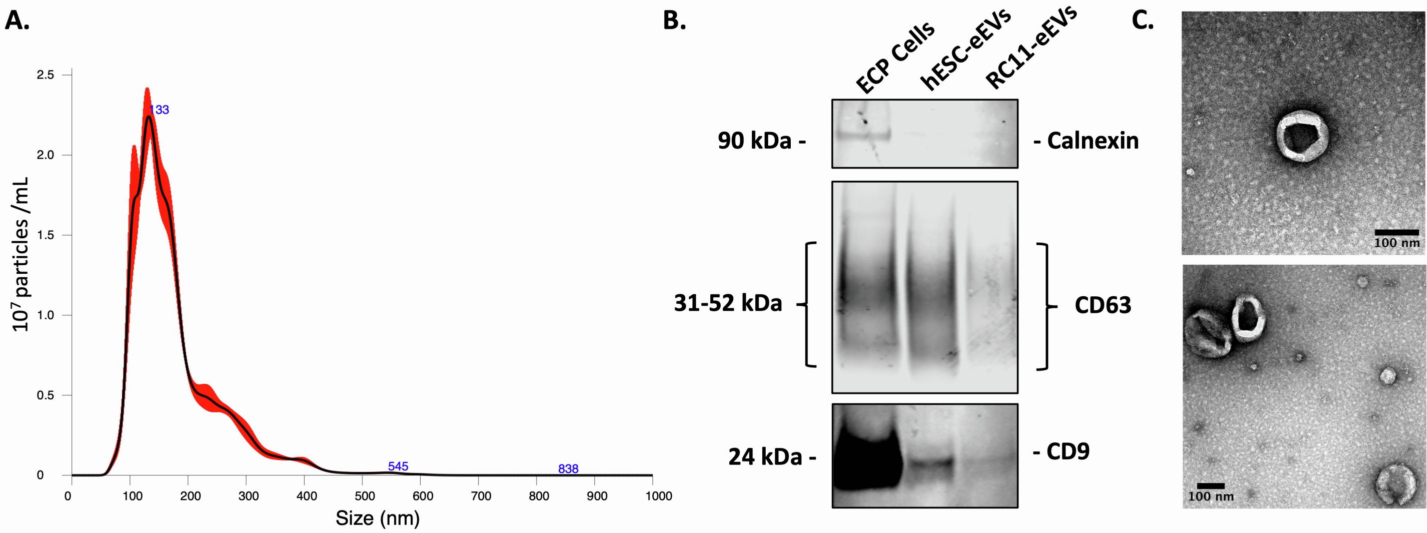

Figure S5. Characterisation of extracellular vesicles from RC11 human embryonic stem cell-derived endothelial cell products (RC11-eEVs).
A. Representative graph from nanoparticle tracking analysis (NTA) showing the size distribution and concentration of RC11-eEVs **B.** EV surface marker characterisation of RC11-eEVs by Western blot. Endothelial cell product lysates were used as a cellular protein control. **C.** Transmission electron microscopy (TEM) of extracellular vesicles from RC11 human embryonic stem cell-derived endothelial cell products (RC11-eEVs). Scale bar 100nm.

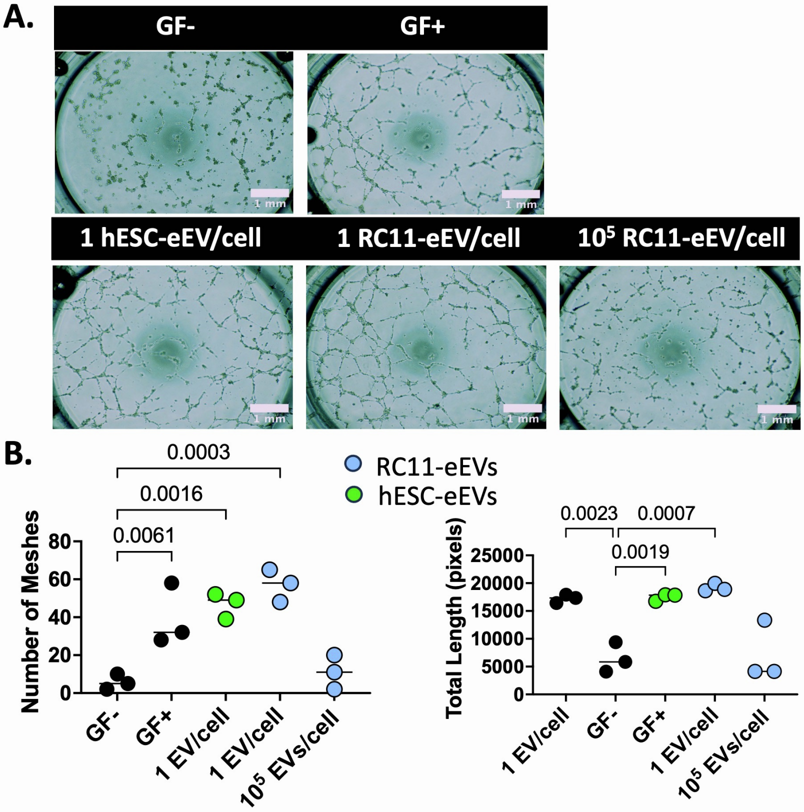

Figure S6. Extracellular vesicles from RC11 human embryonic stem cell-derived endothelial cell products (RC11-eEVs) induce endothelial cell (EC) tube formation at low concentrations. A. Tube Formation Assay on human cardiac microvascular endothelial cells (HCMECs) treated with two EV doses: 1 and 10^5 EVs/cell (n=3). Cells cultured in basal media and treated with sterile filtered PBS (vehicle control) served as negative control (GF-). Cells in fully supplemented media (GF+) or treated with extracellular vesicles from human embryonic stem cell-derived endothelial cell products (hESC-eEVs) served as positive controls. **B.** Quantification of the number of meshes, branches and the total length of the tubes formed at 4h. Images were analysed using the "Angiogenesis Analyzer" tool on ImageJ. The control samples (GF+ and GF-) are depicted as black circles, the hESC-eEV treated samples are depicted as green circles and the RC11-eEV-treated samples are depicted as blue circles. Statistical significance (indicated with p-values) was determined by one-way ANOVA with Dunnett's multiple comparisons test. Error bars represent the SD. Scale bar=1mm.

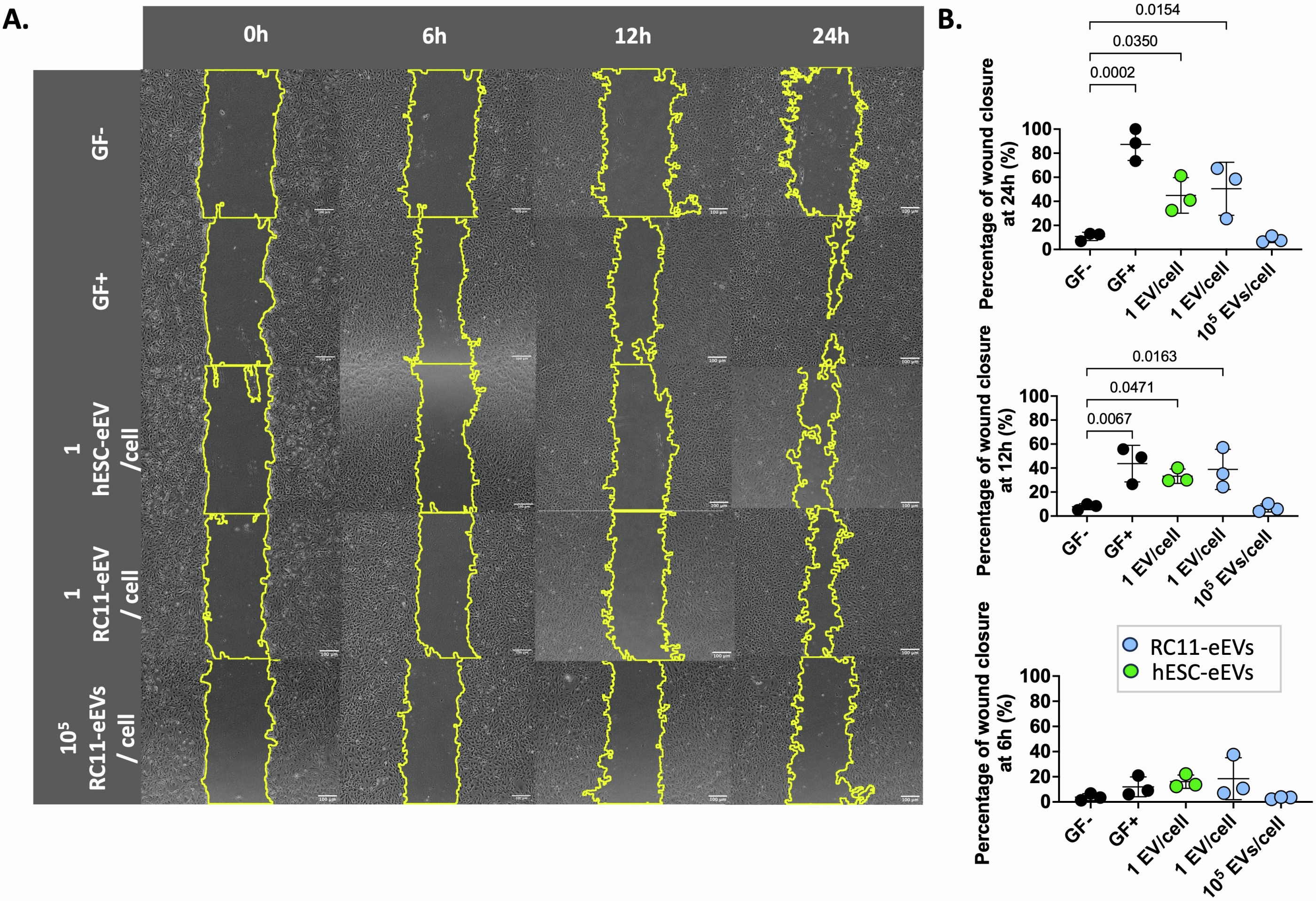

Figure S7. Extracellular vesicles from RC11 human embryonic stem cell-derived endothelial cell products (RC11-eEVs) promote endothelial cell (EC) wound healing at low concentrations. A) Wound Healing Assay on human cardiac microvascular endothelial cells (HCMECs) treated with EVs (n=3). Cells cultured in basal media and treated with PBS (vehicle control) served as negative control (GF-). Cells in fully supplemented media (GF+) or treated with extracellular vesicles from human embryonic stem cell-derived endothelial cell products (hESC-eEVs) served as positive controls. **B)** Quantification of the percentage of wound closure at 6h, 12h and 24h post-wound induction. The control samples (GF+ and GF-) are depicted as black circles, the hESC-eEV treated samples are depicted as green circles and the RC11-eEV-treated samples are depicted as blue circles. Statistical significance (indicated with p-values) was determined by one-way ANOVA with Dunnett's multiple comparisons test. Error bars represent the SD. Scale bar=100µm.

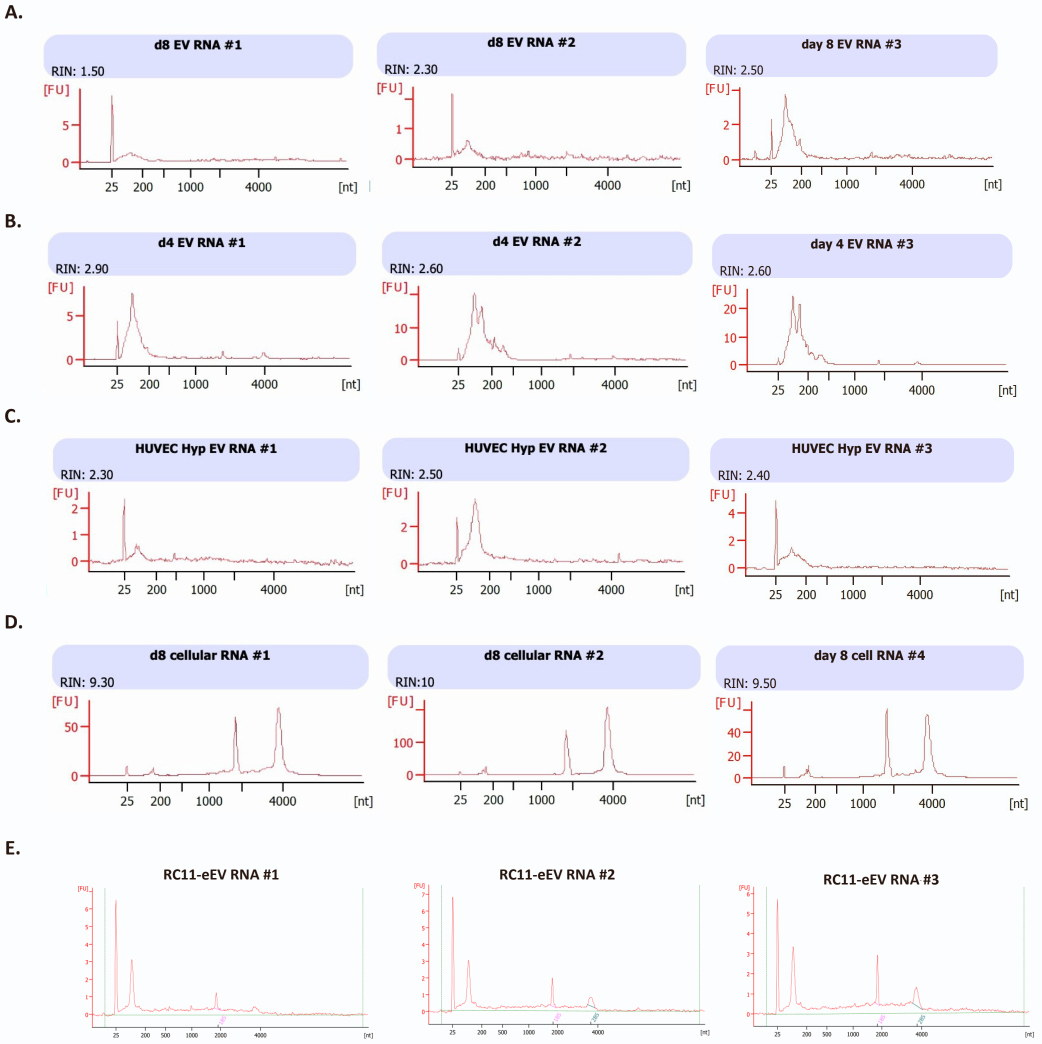

Figure S8. RNA profiling confirms the presence of small and the absence of ribosomal RNA peaks in the extracellular vesicle (EV) samples. A.RNA profiling of human embryonic stem cell-derived endothelial cell product EV (hESC-eEV) RNA samples (d8 EV RNA, n=3). **B.** RNA profiling of human embryonic stem cell-derived mesodermal cell product EV (hESC-mEV) RNA samples (d4 EV RNA, n=3). **C.** RNA profiling of human umbilical vein endothelial cell (HUVEC) hypoxic EV RNA samples (HUVEC Hyp EV RNA, n=3). **D.** RNA profiling of human embryonic stem cell-derived endothelial cell products (hESC-ECPs) RNA samples (d8 cellular RNA, n=3). **E.** RNA profiling of RC11 human embryonic stem cell-derived endothelial cell product EVs (RC11-eEVs , n=3)

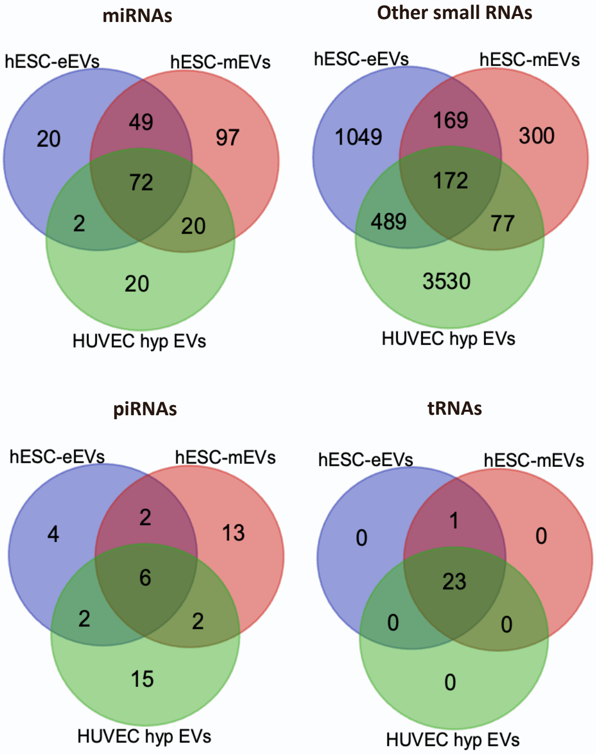

Figure S9. Small RNA sequencing showed a diverse composition of small RNA classes in the extracellular vesicle (EV) samples. Venn diagrams showing unique and common small RNA molecules present in the different EV samples. Molecules with average $RPMM_{total}$ <10 were filtered-out of the analysis.

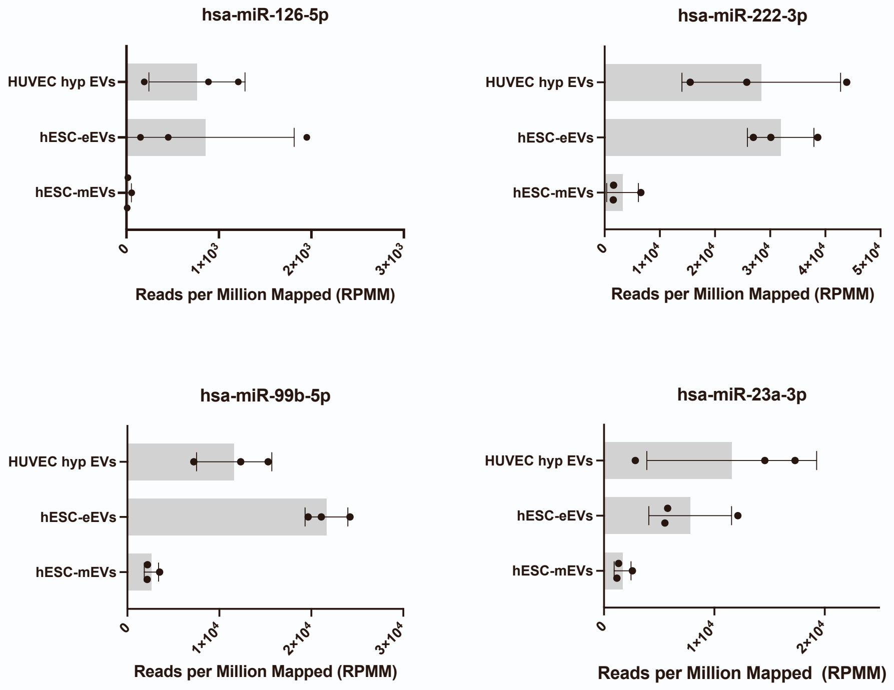

Figure S10. Extracellular vesicles from human embryonic stem cell-derived endothelial cell products (hESC-eEVs) and EVs from hypoxic human umbilical vein endothelial cells (HUVECs) express EC-enriched miRNAs. Plots representing RPMM of EC-enriched miRNAs (miR-126-5p, miR-222-3p, miR-99b-5p, miR-23a-3p) in the EV libraries.

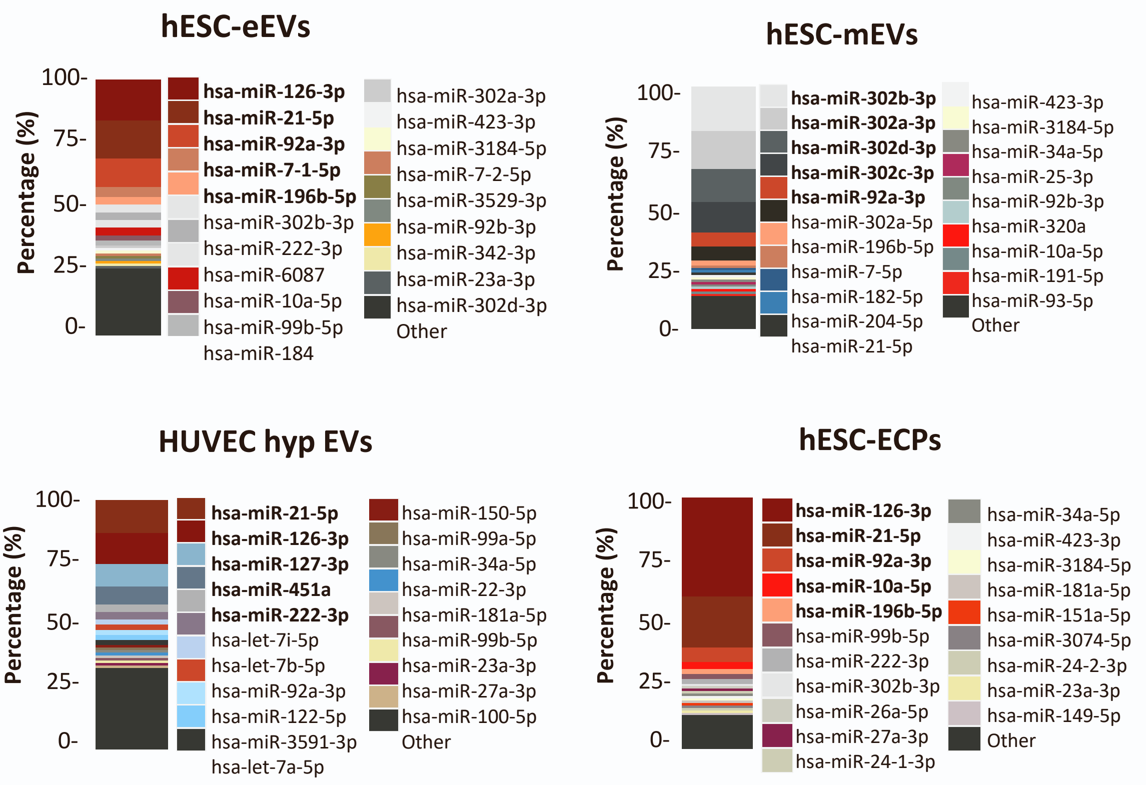

Figure S11. In each dataset the top 3-5 miRNAs occupy 50% of the total miRNA reads. Plots representing the percentage of the reads occupied by the top 20 miRNAs of each EV and cellular RNA sample. In all datasets the top 20 miRNAs correspond to 68-86% of the total miRNA reads. The top 5 miRNAs in each group are in bold. In the stacked bars each miRNA is represented by a different colour to understand the differences and similarities between the top 20 miRNA of each group.

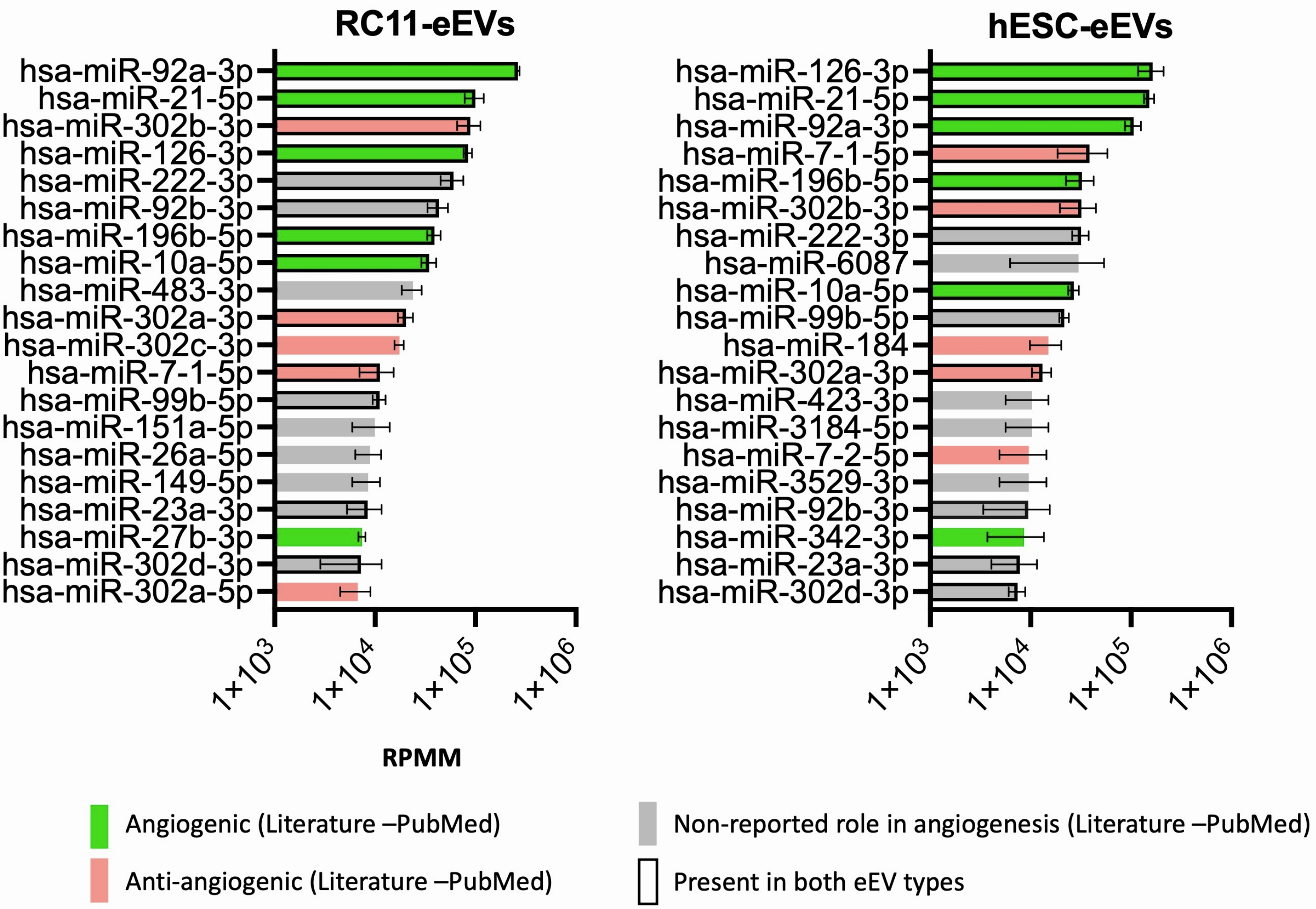

Figure S12. Top 20 microRNA cargo of RC11-eEVs and hESC-eEVs shows 65% overlap

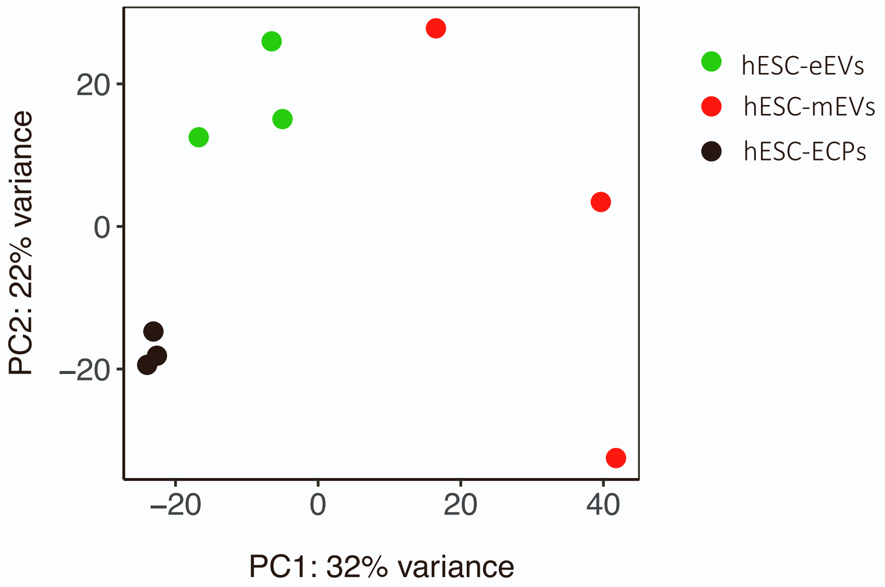

Figure S13. Principal Component Analysis (PCA) of the total reads of the Small RNA Sequencing datasets. Each dataset is depicted by a different colour. Extracellular vesicles from human embryonic stem cell-derived endothelial cell products (hESC-eEVs) are in green, extracellular vesicles from the mesodermal stage of the differentiation system (hESC-mEVs) are in red and human embryonic stem cell-derived endothelial cell products (hESC-EPCs) are in black.

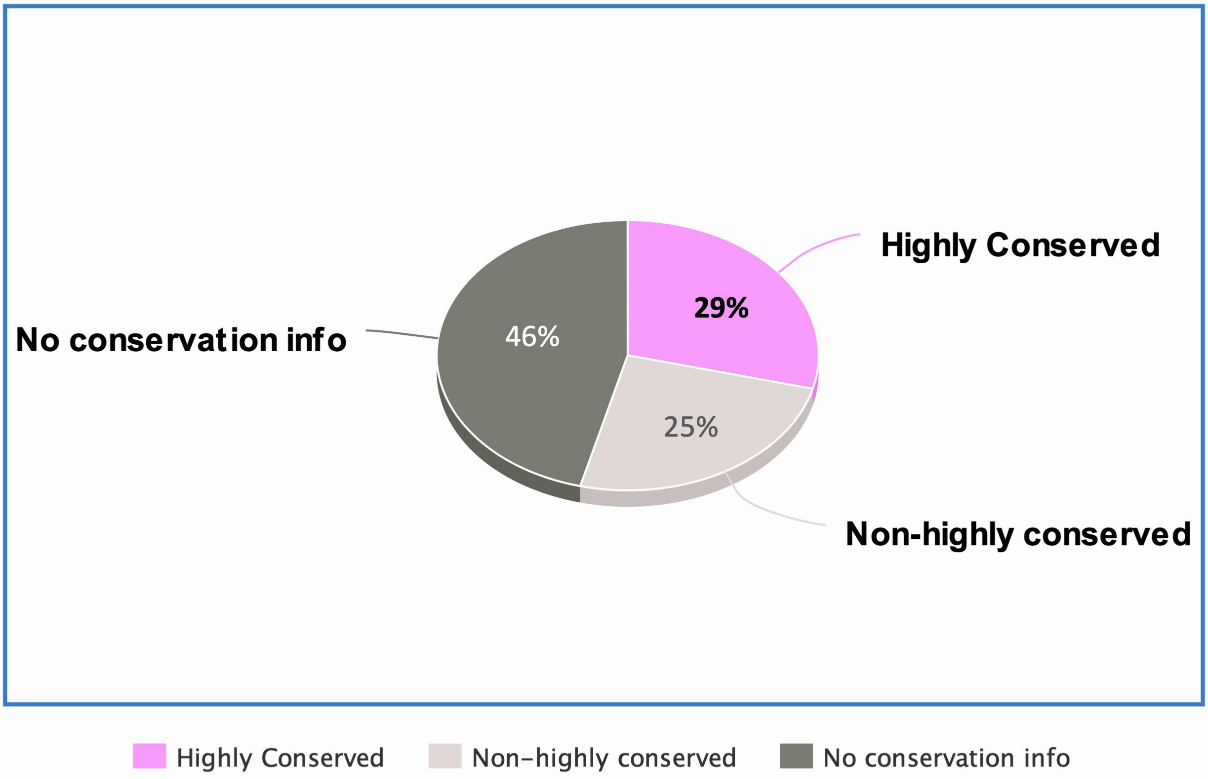

Figure S14. A small subset of human embryonic stem cell-derived extracellular vesicles miRNAs (hESC-eEV-miRNAs) is highly conserved between human and mouse/rat. To investigate the conservation of hESC-eEV-miRNAs between human and mouse/rat, we used the "microRNA viewer" tool. A cut-off of 95% was implemented to identify miRNAs that are highly conserved across the species examined. Dark grey: percentage of miRNAs with no conservation info with the informatic tools currently available. Pink: percentage of conserved miRNAs, Light grey: percentage of non-highly conserved miRNAs

hsa-miR-4496

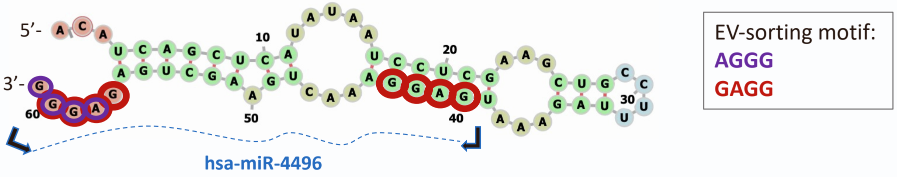

hsa-miR-4691

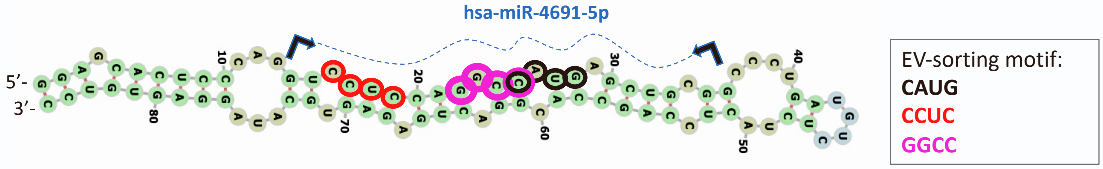

Figure S15. The human embryonic stem cell to endothelial cell extracellular vesicle (hESC-eEV)-enriched miRNAs miR-4496 and miR-4691-5p contain EV sorting motifs. Representation of the secondary structure of precursor miRNAs (pre-miRNAs) hsa-miR-4496 and hsa-miR-4691 generated using miRNAFold. Blue dotted lines represent the sequence of the mature miRNAs hsa-miR-4496 and miR-4691-5p. Nucleotides corresponding to different EV sorting motifs are in circles of different colours. Nucleotides corresponding to the AGGG motif are in dark purple circles. Nucleotides corresponding to the GAGG motif are in dark red circles. Nucleotides corresponding to the CAUG motif are in black. Nucleotides corresponding to the CCUC motif are in red. Nucleotides corresponding to the GGCC motif are in purple.

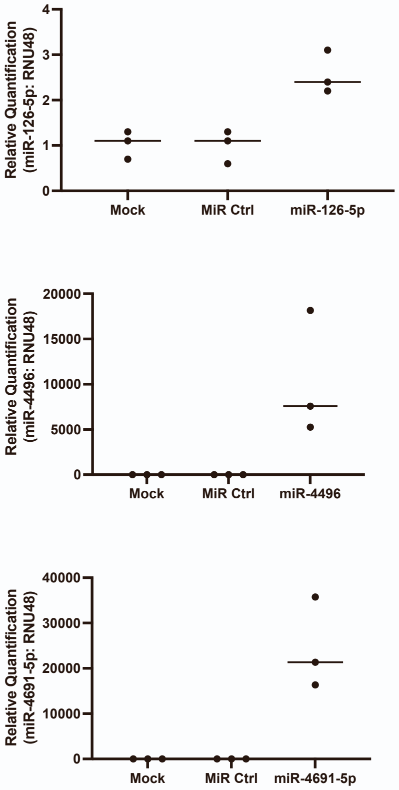

Figure S16. Endothelial cell transfection with miRNA mimics for miR-126-5p or miR-4496 or miR-4691-5p leads to miRNA overexpression. Results of qRT-PCR analysis showing significant upregulation of miR-126-5p or miR-4496 or miR-4691-5p expression in endothelial cells transfected with miRNA mimics compared to cells transfected with a miRNA control (miRC) or treated with lipofectamine RNAiMAX (mock) (n=3). RNU48 was used as a normalisation gene.

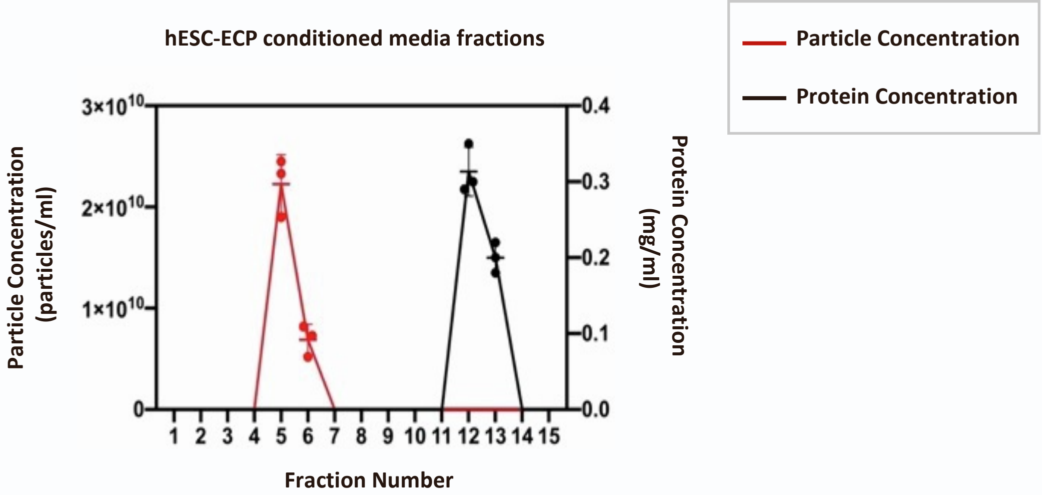

Figure S17. The combination of ultrafiltration with size exclusion chromatography (SEC) results in good separation of the particle and protein containing fractions. The graph depicts the particle and protein concentration of human embryonic stem cell-derived endothelial cell product (hESC-ECP) conditioned media fractions resulting after SEC, as determined by nanoparticle tracking analysis (NTA) and spectrophotometry respectively. Particles are eluted in fractions 5 and 6, while proteins are eluted in fractions 12 and 13.

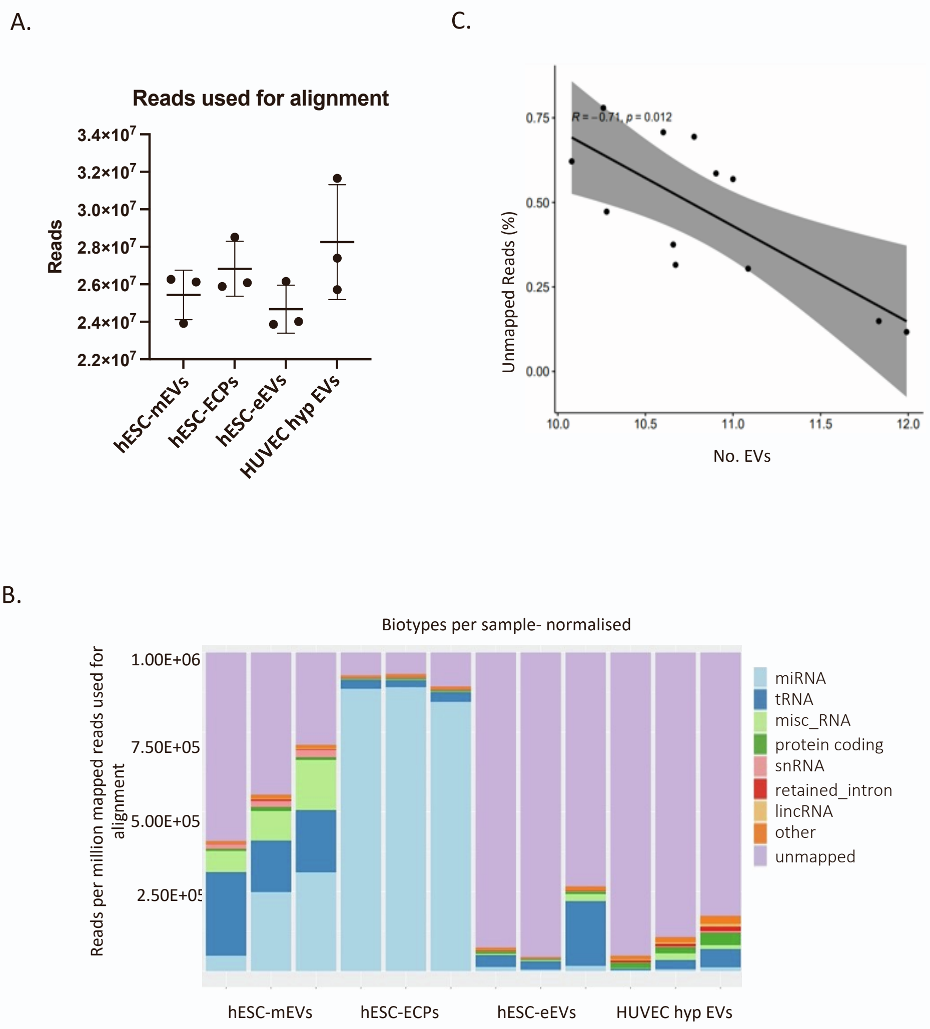

Figure S18. Small RNA sequencing read characteristics. A. RNA input reads used for alignment after filtering out UniVec/rRNA/low quality reads (n=3) **B.** Stacked bar plots representing read composition per sample. Reads are normalised to the total number of reads ($RPMM_{total}$) **C.** Correlation between the percentage of unmapped reads and the number of particles used in the small RNA sequencing of the extracellular vesicle (EV) samples.

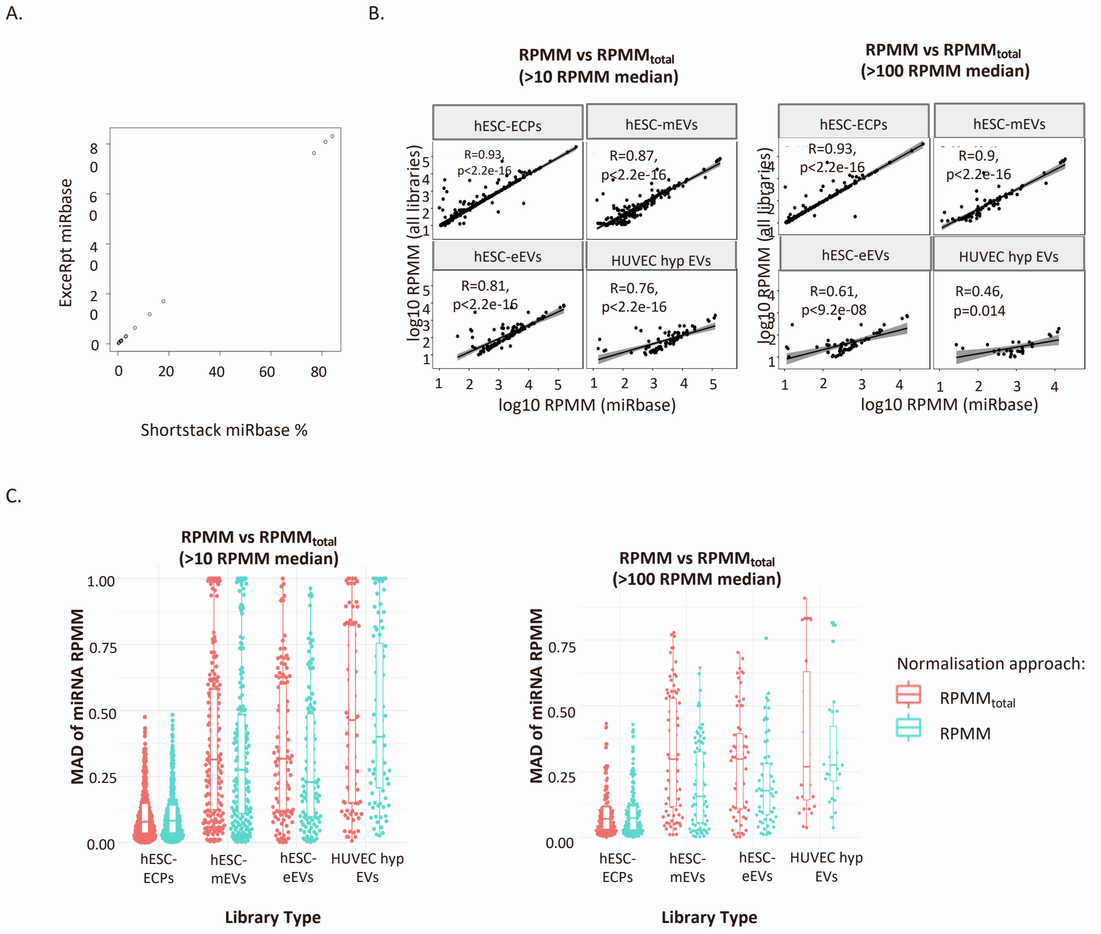

Figure S19. Normalisation to the total miRNA counts (RPMM method) results in lower variability to normalisation to normalisation to all small libraries (RPMM$_{total}$). A. Correlation between the results obtained by 2 normalisation approaches: 1) Normalisation of miRNA reads to the total small RNA reads (excerpt miRbase), 2) Normalisation of miRNA reads to the total RNA reads **B.** Graphs representing correlation between RPMM and RPMM$_{total}$ values in the different libraries **C.** Violin plots showing variability between replicates using RPMM and RPMM$_{total}$ normalisation approaches.

A.

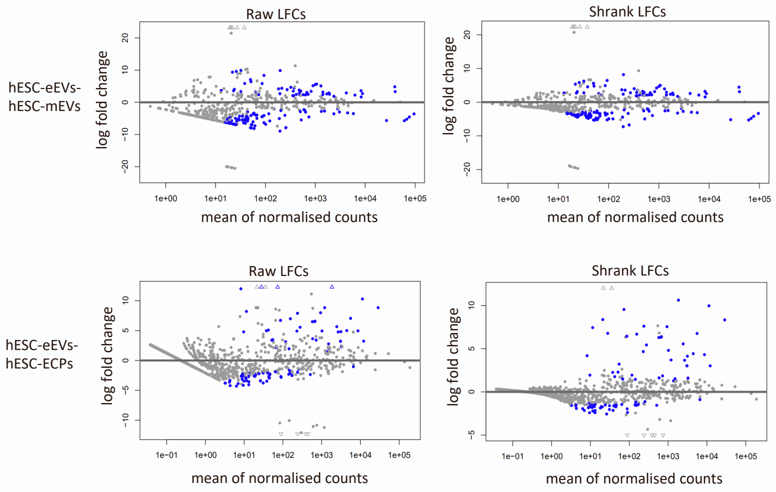

B.

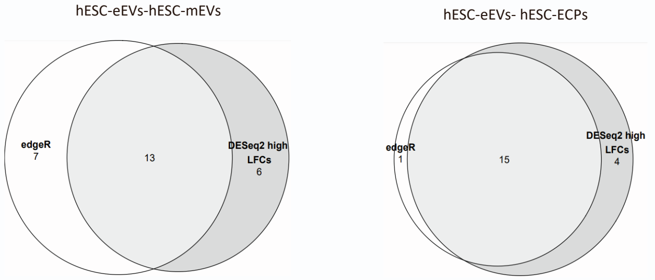

Figure S20. Differential expression analysis using the DESEq2 and edgeR tools yields similar results. A. Comparing log fold changes (LFCs) of miRNAs in the individual comparisons before (left panels) and after (right panels) applying fold change shrinkage via the ashr protocol in DESeq2. **B**. Comparison between DESeq2 and alternative normalisation and differential expression analysis via edgeR, high LFC refers to miRNAs with an LFC >5 or <-5 after shrinkage in DESeq2.

Movie S1. Visualisation of extracellular vesicles from human embryonic stem cell-derived endothelial cell products (hESC-eEVs) using Nanosight.
